# Volatile hiring: uncertainty in search and matching models^☆^

**DOI:** 10.1016/j.jmoneco.2021.07.008

**Published:** 2021-10

**Authors:** Wouter J. Den Haan, Lukas B. Freund, Pontus Rendahl

**Affiliations:** a London School of Economics and Political and Political Science, CEPR and CFM, Department of Economics, Houghton Street, London WC2A 2AE, United Kingdom; b University of Cambridge, Faculty of Economics, Sidgwick Avenue, Cambridge CB3 9DD, United Kingdom; c Copenhagen Business School, Department of Economics, CEPR and CFM Porcelaenshaven 16A, 2000 Frederiksberg, Denmark

**Keywords:** Uncertainty, Search frictions, Unemployment, Option value

## Abstract

In search-and-matching models, the nonlinear nature of search frictions increases average unemployment rates during periods with higher volatility. These frictions are not, however, by themselves sufficient to raise unemployment following an increase in *perceived* uncertainty; though they may do so in conjunction with the common assumption of wages being determined by Nash bargaining. Importantly, option-value considerations play no role in the standard model with free entry. In contrast, when the mass of entrepreneurs is finite and there is heterogeneity in firm-specific productivity, a rise in perceived uncertainty robustly increases the option value of waiting and reduces job creation.

## 1 Introduction

There is a large empirical literature which demonstrates that economic volatility is time-varying and that heightened uncertainty negatively affects labor markets and macroeconomic activity, even when the rise in uncertainty is merely perceived.^1^ Thus, increased uncertainty has been identified as one of the key contributors to historically significant increases in cyclical unemployment such as those occurring in during the COVID-19 pandemic (Baker, Bloom, Davis, Terry, 2020, Leduc, Liu, 2020) and the Global Financial Crisis (Baker et al., 2012).^2^ Yet, several theoretical models and mechanisms predict the opposite. Precautionary motives call forth a rise in savings, which would be associated with increased investment. Also, limited liability means that firm owners’ payoff function is convex, which implies that uncertainty increases firm equity value and makes investment more attractive. The famous “option-value-of-waiting” mechanism, however, does predict a negative relationship between elevated uncertainty and economic activity because higher (anticipated) uncertainty makes it more attractive to wait and postpone investment (cf. Bernanke (1983)).

The aim of this paper is to clarify the transmission mechanisms of uncertainty, and specifically the role of option-value considerations, in the canonical search-and-matching (SaM) model of the labor market. We build on Leduc and Liu (2016), who, in an important contribution, demonstrate that in a standard SaM model an increase in perceived uncertainty leads to an increase in the unemployment rate. In fact, they show that such a model can qualitatively match the empirical movements even under flexible prices and with prudent agents, a combination that, by itself, typically pushes economic activity in the opposite direction.^3^ Leduc and Liu (2016) do not bring to the surface what mechanism lies behind this result. But they conjecture that search frictions in the labor market produce an option-value channel that helps rationalize the adverse effects on economic activity of elevated uncertainty.

The first contribution of this paper is to provide an in-depth analysis of the effects of increased volatility in SaM models, examining in particular whether there is an option-value channel. Since job creation is very much like an irreversible investment, as the associated costs are not refundable, the option-value channel is a sensible candidate to consider. Nonetheless, the nonlinear aspects of the standard SaM model themselves do *not* lead to any option value of waiting. The reason is that the free-entry assumption implies that the expected value of vacancy posting is, and will always remain, equal to zero. Hence, there is no point in waiting.

We show that Leduc and Liu’s (2016) result, that an increase in perceived uncertainty leads to an increase in the unemployment rate in a standard SaM model with flexible prices, depends crucially on the assumption that wages are determined by Nash bargaining, an assumption that is often adopted in the SaM literature. However, many other types of wage setting assumptions are possible. Specifically, we show that changes in perceived uncertainty have *no* effect on job creation when wages are linear in productivity. Nonlinear matching frictions do imply that the *average* value of labor market tightness – the number of vacancies relative to the number of unemployed workers searching for a job – is elevated during periods of higher *realized* volatility. Under Nash bargaining, this improves workers’ bargaining position and raises the average wage. Even if higher volatility is not realized, the increase in wages is driven by what agents *expect* to happen when perceived uncertainty increases. As a result, the firm value falls, which reduces job creation.

The literature often focuses on the impact of an increase in *perceived* uncertainty; that is, the impact that is solely due to beliefs, not to an actual increase in volatility. Our second contribution is to highlight the importance of analyzing the impact of *realized* increases in volatility (measured as the impact averaged over all possible realizations). As mentioned above, how perceived uncertainty affects the economy depends on what agents *expect* to happen during the period of heightened volatility. Furthermore, both in the standard SaM framework and in the modifications we consider, we find that *even if* the anticipation of uncertainty itself does have a non-zero impact on the economy, these effects are small compared to those induced by realized volatility. In addition, the effects of realized and perceived volatility can differ in sign along the Impulse response function (IRF) over at least some horizons.

Our third contribution is to demonstrate how wait-and-see considerations can be introduced into the SaM framework. Our starting point is to observe that virtually all SaM models assume that there are always enough potential entrepreneurs available to drive the expected profits of job creation to zero. We first highlight that an option-value channel is in principle possible by relaxing the free-entry condition and assuming that the mass of entrepreneurs is finite.^4^ However, the resulting channel is only operative under restrictive assumptions. In particular, the free-entry condition must be binding in some states, such that firms make zero profits, but not in others, such that firms make positive profits. In a second step, we therefore add heterogeneity in idiosyncratic firm-productivity alongside the assumption of a finite mass of entrepreneurs. In the resulting SaM framework, there is a time-varying measure of entrepreneurs that expect to make strictly positive profits when they post a vacancy, namely those with a sufficiently high productivity draw. With this relatively simple modification, the model robustly predicts that perceived uncertainty leads to a postponement of job creation. The reason is that an expected increase in future volatility increases an unmatched entrepreneur’s (future) chance of having a productivity draw for which expected profits of vacancy-posting are strictly positive, whereas the downside risk is not affected since unmatched entrepreneurs can always choose to stay out of the market. Our proposed model remains tractable and can be solved by (higher-order) perturbation methods.

Our study is connected to two broad strands of the economic literature, namely, analyses considering frictional labor market models and the effects of uncertainty, respectively. While each of these literatures is vast in scope, we briefly comment on the most closely related studies. In particular, Bloom (2009) offers a seminal examination of the sort of real-options effects analyzed also in this paper. His model incorporates option-value considerations in hiring and (physical) investment due to non-convex adjustment costs. We consider a particularly prominent variant of adjustment costs, namely search frictions in the labor market, and identify the conditions under which an option-value effect materializes, and when it does not.

Considering studies that share this focus beyond Leduc and Liu (2016), the most closely related paper is Schaal (2017). That paper develops a model with multi-worker firms that are heterogeneous in productivity and which are subject to an endogenous linear hiring cost at the firm level.^5^ In similarity to our proposed model, the resulting irreversibility gives rise to an option value of waiting. In contrast to our approach, the free-entry condition binds in every state of the world. The reason that the value of vacancy posting nonetheless varies over time in Schaal’s (2017) setup is that firms operate a decreasing returns to scale technology, and the free-entry condition obtains at the level of the (multi-worker) firm rather than at the vacancy level. We, by contrast, stay as close as possible to the canonical Diamond-Mortensen-Pissarides assumptions of constant returns to scale in production and random search, while restoring an option-value channel.^6^

The next section lays out a standard SaM model and its calibration. Section 3.1 reports the effects on the economy both to an increase in perceived and realized uncertainty, respectively. Sections 3.2–3.4 analyze these results in detail. Section 4 discusses our modifed SaM model in which there is an option value of postponing job creation. The last section concludes.

## 2 Theoretical framework

We begin by summarizing a basic search-and-matching (SaM) framework. This is the same model as studied by Leduc and Liu (2016), except that we restrict ourselves to the flexible-price version and assume the representative household to be risk neutral. Both assumptions are common in the matching literature. For us they have the benefit of making the analysis more transparent. Specifically, as shown in Bernanke (1983) and our example in Section 3, the option value of waiting does not rely on risk aversion.^7^ Nor are sticky prices necessary.

*Model equations.* The model is characterized by four equations in four unknown variables, $J_{t}$, $w_{t}^{N}$, $\theta_{t}$, and $n_{t}$.

(1) $$J_{t}=\bar{x}z_{t}-w_{t}^{N}+\beta E_{t}\left[J_{t+1}\left(1-\delta \right)\right],$$

(2) $$w_{t}^{N}=\omega (\bar{x}z_{t}+\beta \left(1-\delta \right)\kappa E_{t}\left[\theta_{t+1}\right])+\left(1-\omega \right)\chi ,$$

(3) $$\kappa =h\left(\theta_{t}\right)J_{t},$$

(4) $$n_{t}=\left(1-n_{t-1}+\delta n_{t-1}\right)f\left(\theta_{t}\right)+\left(1-\delta \right)n_{t-1}.$$

Here, $J_{t}$ denotes firm value; $z_{t}$ the (exogenous) labor productivity; $w_{t}^{N}$ the wage rate based on Nash bargaining; $\theta_{t}$ labor market tightness; and $n_{t}$ the mass of productive relationships (or employment).^8^

The firm value, $J_{t}$, is simply the present-discounted value of firm profits, using the household’s discount factor, β, and taking into account that firms separate at the exogenous rate δ. The parameter χ in the wage expression represents the benefit that accrues to workers if they are not employed. If the bargaining weight of the worker, ω, is equal to zero, wages are simply equal to χ, which therefore serves as a floor on wages. If the bargaining weight of the worker, ω, exceeds zero, however, the wage rate increases with the firm’s marginal revenues, $\bar{x}z_{t}$, as well as with the expected value of future market tightness.^9^

The job finding rate, $f(\theta_{t})$, and the hiring rate, $h(\theta_{t})$, are determined in the matching market. The number of matches in period t is determined by the matching function $m_{t}=\psi v_{t}^{1-\alpha }{\left(u_{t}^{s}\right)}^{\alpha }$, where $v_{t}$ denotes the number of vacancies and $u_{t}^{s}$ the measure of workers searching for a job; that is, $u_{t}^{s}=1-n_{t-1}+\delta n_{t-1}$. This functional form implies that $h_{t}=\frac{m_{t}}{v_{t}}=\psi \theta_{t}^{-\alpha }$ and $f_{t}=\frac{m_{t}}{u_{t}^{s}}=\psi \theta_{t}^{1-\alpha }$, where $\theta_{t}$ indicates labor market tightness, $\theta_{t}=\frac{v_{t}}{u_{t}^{s}}$.

The cost of posting a vacancy is equal to κ and its expected benefit is equal to the product of the hiring rate, $h_{t}$, and the value that accrues to a successful entrepreneur, $J_{t}$. The standard assumption of SaM models is that there is a potentially infinite number of entrepreneurs. This means that κ has to equal $h_{t}J_{t}$, as indicated by the free-entry condition in equation (3). The free-entry condition implies that an increase in $J_{t}$ leads to a lower value of $h_{t}$, which is accomplished by an inflow of additional entrepreneurs into the matching market, i.e., an increase in $v_{t}$ and a reduction in $\theta_{t}$.

Finally, the value of the exogenous variable $z_{t}$ is determined by the following process

(5) $$z_{t}=\left(1-\rho_{z}\right)\bar{z}+\rho_{z}z_{t-1}+\sigma_{t-1}\varepsilon_{z,t},$$

(6) $$\ln(\sigma_{t})=\left(1-\rho_{\sigma }\right)\ln\left(\sigma \right)+\rho_{\sigma }\ln\left(\sigma_{t-1}\right)+\sigma_{\sigma }\varepsilon_{\sigma ,t},$$

where $\varepsilon_{z,t}$ and $\varepsilon_{\sigma ,t}$ are *iid* standard Normal processes. The steady-state value of productivity, $\bar{z}$, is normalized to unity. Uncertainty shocks are associated with changes in $\varepsilon_{\sigma ,t}$. This specification of the stochastic processes is common in the literature but deviates from Leduc and Liu (2016) in two respects. First, the process for $z_{t}$ is in levels rather than in logarithms to prevent the expected value of productivity to differ from its steady-state through a Jensen’s inequality effect. Second, we use the timing assumption common in the uncertainty literature according to which volatility shocks have a delayed impact on the distribution of productivity shocks (e.g., Bloom (2009)). We do so to underscore that real options effects are absent even under a timing assumption that is, in principle, favorable to wait-and-see effects (cf. Schaal (2017, footnote 12)). As in Leduc and Liu (2016), we specify the process for $\sigma_{t}$ in logs to ensure that the standard deviation remains positive.

*Calibration and solution method.* The calibration follows Leduc and Liu (2016) as closely as possible. The calibrated parameter values and the associated targets/outcomes are reported in Table 1. We also use the same solution method, that is, third-order pruned perturbation.

## 3 Volatility in the standard search-and-matching model

The main objective of this section is to present and analyze the effect of volatility shocks in the standard search-and-matching (SaM) model. We proceed in four steps. First, we illustrate IRFs of the baseline model, and outline a distinction between the total volatility effects and those that arise purely from anticipation. As we will see, increased uncertainty generally leads to a decline in firm value and a rise in unemployment. However, the results are more complex with respect to variables such as labor market tightness and wages. Next, we provide a simple two-period version of the model to illustrate when an option-value channel may emerge, and show that these conditions are not met in the SaM framework. Thus, the decline in economic activity revealed by the IRFs is not due to an option-value effect. Third, we show that *all* anticipation effects disappear once we replace Nash bargaining with a wage rule that is linear in productivity. Lastly, we explain why Nash bargaining can have non-trivial implications for the transmission of uncertainty shocks.

### 3.1 Impulse response functions

**Fig. 1:**
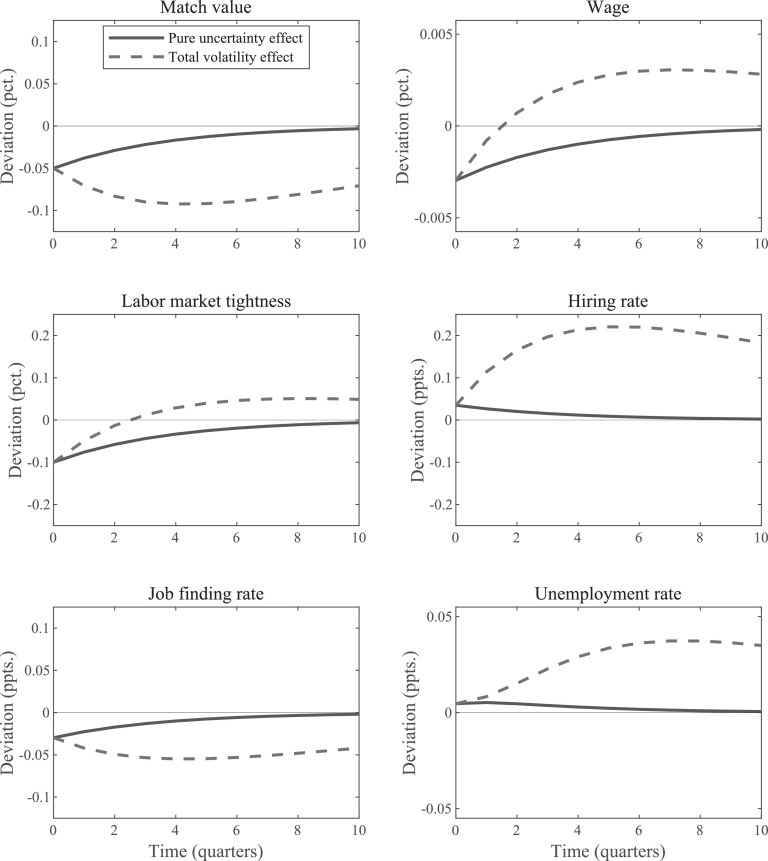
IRFs for uncertainty shock in standard SaM model under Nash Bargaining. *Notes:* The “total volatility” IRFs plot the change in the period-0 expected values of the indicated variables in response to a unit-increase in $\varepsilon_{\sigma ,t}$. The “pure uncertainty” IRFs display how the economy responds when agents think volatility will increase, but the higher volatility actually never materializes.

Fig. 1 plots the IRFs for an uncertainty shock, that is, an increase in $\varepsilon_{\sigma ,t}$. We plot two different types of IRFs. The first, the *total volatility* IRF, of variable $x_{t}$ is the standard IRF that plots $E_{\tau }\left[x_{\tau +j}\right]$, where τ is the period the shock occurs and j=0,1,⋯. These IRFs describe what happens on average (or, equivalently, in *expectation*) during periods of heightened volatility. Whereas in linear models the impact of a period-τ shock on variables in subsequent periods does *not* depend on realizations of future shocks, in nonlinear models it does. This means that one has to integrate over all possible future realizations to calculate this *expected* impact of a period-τ shock.^10^

The second type of IRFs, the *pure uncertainty* IRF, plots the response of the economy when agents perceive an increase in future volatility, but this increase never materializes. For an IRF that uses the stochastic steady state as the starting point, this means that agents think $\sigma_{t}$ is higher than normal during the periods following the shock and act accordingly, but productivity, $z_{t}$, remains at its steady-state value. Thus, the pure uncertainty IRF measures the effects of an increase in *purely anticipated* uncertainty, as examined also by Leduc and Liu (2016). These effects arise solely due to agents’ responses to changed expectations about the future as described by the total volatility IRF. Thus, the latter type of IRFs is essential to understand the first kind.^11^

The key observations about Fig. 1 are as follows. First, the value of a firm falls and the unemployment rate increases. This is true for both types of IRFs. Second, there are important qualitative differences between the two types of IRFs. Whereas the pure uncertainty IRFs follow the usual monotone pattern, the total volatility IRFs display an inverted u-shape. Moreover, for the wage rate and tightness variable, the two types of IRFs even have different signs at some horizons.^12^ Both types of IRFs take on negative values initially for these two variables, but total volatility IRFs turns positive soon after the shock occurs whereas this is not the case for the pure uncertainty IRFs. As explained in the next section, this observation is important to understand why the firm value drops in the matching model with Nash bargaining when volatility increases or is anticipated to do so.

### 3.2 The evasive option value

To understand the IRFs presented in the last section, we make use of a simple example to illustrate why and when an increase in uncertainty increases the option value of postponing workforce investment. As we will see, there cannot be an option-value channel operating within the standard SaM framework, and the example developed here is useful to make this clear.

*Option value of postponing investment.* The option value to wait is most transparent under risk neutrality, as risk aversion will add additional aspects to the analysis, such as precautionary savings and changes in risk premia. Hence, we consider a risk neutral agent. This agent can choose between the following two investment paths. The first possibility consists of investing immediately and earning a known return $R_{1}$ in the first period and a stochastic return $R_{2}$ in the second period. The latter return will only become known in period 2. Alternatively, the agent can postpone making a decision. In this case, she would instead bring the money to the bank in the first period and earn a return equal to $R^{*}<R_{1}$. In the second period, the agent will invest in the project only if $R_{2}>R^{*}\geq 0$. The expected values of the two strategies – *commit* and *wait* – are given by

(7) $$J_{\text{commit}}=R_{1}+\beta E\left[R_{2}\right],$$

(8) $$J_{\text{wait}}=R^{*}+\beta E\left[\max\left\{R_{2},R^{*}\right\}\right].$$

How does increased volatility, i.e., an increase in the variance of $R_{2}$, affect the entrepreneur’s choice when we keep the *expected value* of $R_{2}$ the same? It does not affect the value of $J_{\text{commit}}$. However, it does increase the value of $J_{\text{wait}}$. The reason is that by waiting the entrepreneur is ensured of a minimum return, $R^{*}$, but she benefits from the higher upward potential of the investment project.

We want to highlight two features that are important. First, the decision is *irreversible*. That is, if the entrepreneur starts the project in period 1, then she cannot unwind the project in period 2 and get a refund. Second, the projects are *mutually exclusive*. That is, the entrepreneur has to adopt either the commit or the wait strategy.

*Option value of waiting in search-and-matching models.* For comparison purposes, consider a two-period version of the standard SaM model.^13^ An entrepreneur who invests by creating a vacancy in period 1 faces the cash flow

(9) $$-\kappa +h_{1}\underset{J_{1}}{\underset{︸}{\left(R_{1}+\beta E\left[R_{2}\right]\right)}},$$

where $R_{t}$ is now equal to profits net of wages. An entrepreneur who waits has no income in period 1 and we get

(10) $$0+\beta E[\max\left\{-\kappa +h_{2}\underset{J_{2}}{\underset{︸}{R_{2}}},0\right\}].$$

Investments are irreversible in the SaM model, since κ is paid upfront. Does this mean that individual entrepreneurs in SaM models have a benefit of waiting when the expected volatility of period-2 profits increases keeping their expected value constant? The answer is no. The free-entry condition implies that expected profits are equal to zero in every time period and in every state of the world; that is, $-\kappa +h_{t}J_{t}=0$, t=1,2. Since profits from vacancy-posting are expected to always be equal to zero, the upward potential that increased the value of waiting in the example discussed above does not exist here. That is, with free entry the last two equations can be written as

(11) $$-\kappa +h_{1}J_{1}=0,$$

(12) $$\beta E\left[\max\left\{-\kappa +h_{2}J_{2},0\right\}\right]=\beta E\left[\max\left\{0,0\right\}\right]=0.$$

Thus, although job creation is irreversible, it is not sufficient to generate an option-value channel.^14^

Key for the result that the expected value of vacancy posting is zero is that investing now and waiting are *not* mutually exclusive. That is, posting a vacancy this period does not prevent vacancies from being posted next period. It would not make a difference if these choices were mutually exclusive for the entrepreneur herself, that is, if one assumed that each entrepreneur can be involved in one project only. The reason is that there are always *other* entrepreneurs who can pursue the alternative choices, exhausting all positive profits. Thus, mutual exclusivity applies – respectively, does not apply – to the economy *as a whole*, and not to individual agents.

In Section 4, we will show that it is possible for the SaM model to have an option-value mechanism if one assumes that each matched entrepreneur cannot be involved in more than one project *and* the mass of entrepreneurs is finite. This is sufficient to create an environment in which projects are mutually exclusive and expected profits are potentially positive.

Fig. 1 demonstrates that there is one aspect of the properties of the SaM model developed in Section 2 that is quite different from the analysis based on the simple two-period setup. Specifically, Fig. 1 documents that the value of a match, $J_{t}$, declines in response to an anticipated uncertainty shock, whereas the value of investing early in the two-period model, $J_{1}$, remains unaffected.

One might conjecture that the reason behind this decline in $J_{t}$ is an increase in the option value of waiting (Leduc and Liu, 2016, p. 21). But $J_{t}$ in the matching model corresponds to $J_{\text{commit}}$ in the simple model; that is, to the value of investing *now*. In contrast, the idea of the option value to wait is that the value of waiting and potentially investing later increases. In the terminology of our stylized setup, an increase in uncertainty leads to an increase in $J_{\text{wait}}$, not to a decrease in $J_{\text{commit}}$.

### 3.3 If it is not an option value, what is it?

Our discussion above made clear that the environment of the standard SaM model does not satisfy the conditions that generate an option value of postponing job creation. But it is still the case that volatility shocks lower the match value and increase the unemployment rate. The question is why does this happen, and can the reason still be given some option-value interpretation?

Note that in the two-period model, we assumed that $E[R_{2}]$, i.e., expected profits, remain the same when we increased the expected volatility. The same is true for expected values of future productivity in the full dynamic models. Thus, it must be the case that the behavior of wages is essential for understanding the results in Fig. 1.

The Nash bargaining assumption adopted in Leduc and Liu (2016) is just one of many possibilities and it is not an essential characteristic of the matching mechanism. Those essentials are, firstly, that neither workers nor entrepreneurs find a match with probability one. And secondly, that both sides face congestion effects, so that the probability of finding a match decreases if more of your ypre are searching; the matching function is concave in both arguments. To better understand the role of uncertainty in SaM models, we first consider the case in which not only the expected value of productivity but also the expected value of profits remains unchanged when volatility increases. This can be easily accomplished if one assumes that wages are a linear function of current productivity, $z_{t}$, only.^15^ Specifically,

(13) $$w_{t}=\omega \bar{x}z_{t}+\left(1-\omega \right)\chi .$$

*Matching frictions and anticipated volatility changes*Under this wage rule one can derive a useful, analytical expression for $J_{t}$.

Proposition 1 *Suppose that wages are set by the linear wage rule given in equation*(13)*, then*

(14) $$J_{t}=\frac{\left(1-\omega \right)\bar{x}}{1-\beta \left(1-\delta \right)\rho_{z}}z_{t}-\frac{(1-\omega )\chi }{1-\beta (1-\delta )}+\frac{\beta \left(1-\delta \right)\left(1-\omega \right)\left(1-\rho_{z}\right)\bar{x}}{\left(1-\beta \left(1-\delta \right)\right)\left(1-\beta \left(1-\delta \right)\rho_{z}\right)}.$$

Proof See online appendix OA. □

**Fig. 2:**
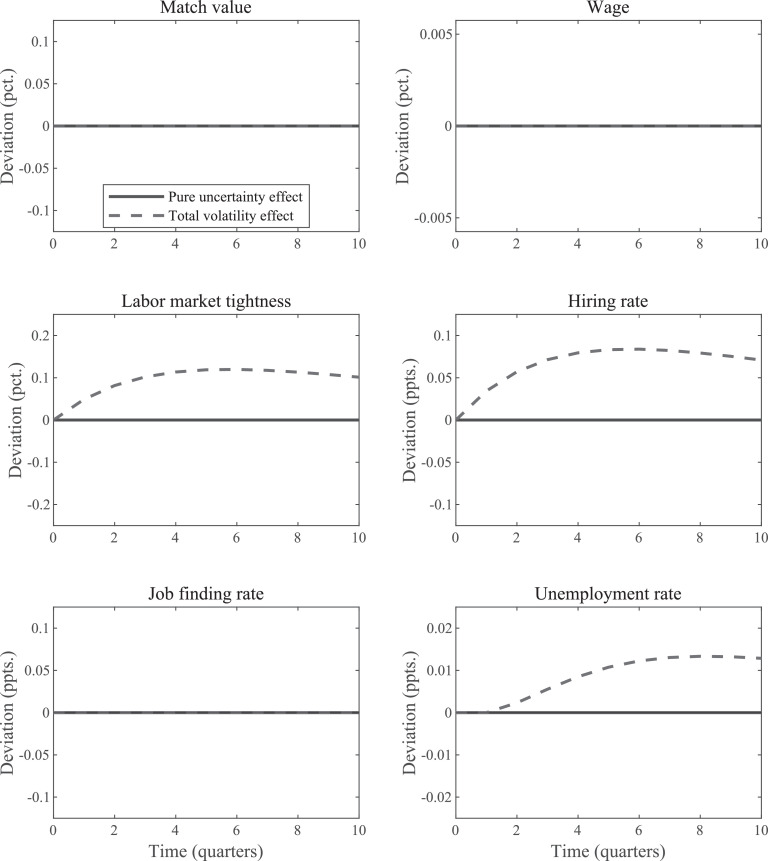
IRFs for uncertainty shock in standard SaM model with linear wage rule. *Notes:* The “total volatility” IRFs plot the change in the period-0 expected values of the indicated variables in response to a unit-increase in $\varepsilon_{\sigma ,t}$. The “pure uncertainty” IRFs display how the economy responds when agents think volatility increases, but the higher volatility actually never materializes.

Thus, $J_{t}$ is a linear function of $z_{t}$. The formula immediately makes clear that an increase in *anticipated* volatility has no effect on $J_{t}$. If the anticipated increase in volatility does not materialize, then $J_{t}$ will not change in subsequent periods either even when agents continue to anticipate higher uncertainty in the future. Consequently, none of the other variables will be affected either, as is documented in Fig. 2 which plots the two types of IRFs for an increase in uncertainty under the linear wage rule.^16^

The fact that the IRFs associated with an anticipated increase in volatility are zero in every period allows us to draw a strong conclusion. That is, the nonlinearity of the matching function by itself does *not* generate an employment effect in response to an increase in anticipated uncertainty. Consequently, there is also no option-value channel associated with the pure anticipation effect of an increase in uncertainty.

Still, increased volatility in productivity will make $J_{t}$ more volatile, which in turn renders matching probabilities more volatile as well. We therefore explore next whether the nonlinearities of the matching function could be such that increases in volatility affect the expected values of employment during the period of elevated volatility.

*Matching frictions and realized volatility changes.* How can we expect an increase in the standard deviation of productivity shocks to affect values of key variables in the model during the period of higher volatility? Given the linearity of $J_{t}$, the total volatility IRF of $J_{t}$ will also be zero. However, as demonstrated by Fig. 2, there are increases in the expected values of market tightness, $\theta_{t}$, the hiring rate, $h_{t}$, and the unemployment rate, $u_{t}$. But the expected values of the job finding rate, $f_{t}$, are unaffected. To understand these results recall the expressions for $\theta_{t}$, $h_{t}$, and $f_{t}$,

$$h_{t}=\frac{\kappa }{J_{t}},\;\theta_{t}={\left(\frac{\psi }{\kappa }J_{t}\right)}^{\frac{1}{1-\alpha }},\text{and}\;f_{t}=\psi {\left(\frac{\psi }{\kappa }J_{t}\right)}^{\frac{1-\alpha }{\alpha }},$$

where α is the curvature parameter in the matching function. The hiring rate, $h_{t}$, is a convex function of $J_{t}$, for any value of α∈(0,1). For our linear wage function this means that it is also convex in $z_{t}$. Consequently, a rise in volatility then leads to an increase in expected values.

Tightness, $\theta_{t}$, is also a convex function of $z_{t}$ for any value of α∈(0,1). When $J_{t}$ is small, for instance, an increase in $J_{t}$ leads to small increases in vacancies. The reason is that small values of $J_{t}$ are associated with low values of $v_{t}$. This implies a high marginal “productivity” of the matching function, so that small changes in the level of $v_{t}$ are sufficient to restore the equilibrium conditions.

By contrast, $f_{t}$ can either be a convex or a concave function of $z_{t}$ depending on the value of α. Our results are based on α=1/2 in which case the job finding rate is linear in $J_{t}$ and, thus, in $z_{t}$. This explains why the total volatility IRF for $f_{t}$ is zero at all forecast horizons. The reason for the ambiguity and the dependence on the value of α is that the hiring rate is inversely related to $J_{t}$ yet the job finding rate is inversely related to the hiring rate.

We now turn our attention to the effect of uncertainty on the employment rate, $n_{t}$. We repeat its law of motion for convenience.

$$n_{t}=\left(1-\delta \right)n_{t-1}+\left(1-\left(1-\delta \right)n_{t-1}\right)f_{t}.$$

Although $f_{t}$ always becomes more volatile, its expected value remains the same when α=1/2. But the total volatility IRFs indicate that this higher volatility is associated with a higher unemployment rate and, thus, lower employment rates. Why does an increase in the volatility of $f_{t}$ reduce the expected future values of $n_{t}$? The reason is that the higher values of the job finding rate are expected to occur during expansions when fewer workers are searching for a job. Consequently, the impact on the employment rate will be smaller. By contrast, the lower values of the job finding rate will have a bigger impact because they are expected to occur during recessions when lots of workers are searching for a job.^17^In the period of the shock, the mass of searching workers, $1-\left(1-\delta \right)n_{t-1}$, is fixed and, hence, a higher volatility of $f_{t}$ has no effect on expected employment. In the next few periods, this mass is still close to its steady-state value. But as time goes on, the asymmetric effect becomes more important when $z_{t}$ shocks push unemployment either up or down. This explains the gradual increase for the unemployment IRF.

**Fig. 3:**
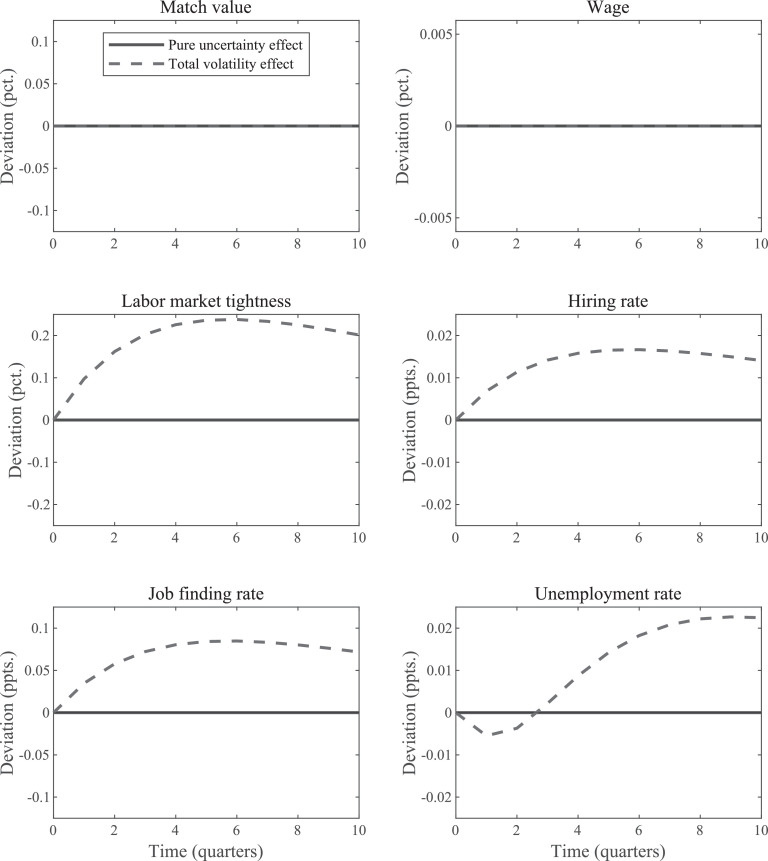
IRFs for uncertainty shock in standard SaM model with linear wage rule & low α. *Notes:* The “total volatility” IRFs plot the change in the period-0 expected values of the indicated variables in response to a unit-increase in $\varepsilon_{\sigma ,t}$. The “pure uncertainty” IRFs display how the economy responds when agents think volatility increases, but the higher volatility actually never materializes. The value of α is equal to 0.2.

*Why increased uncertainty might reduce unemployment due to matching frictions.* When α=1/2, then the increased volatily has no effect on the average value of $f_{t}$. However, when α<1/2, then $f_{t}$ is a convex function of $z_{t}$, which implies that the expected values of the job finding rate increases. Fig. 3 plots the results when α=0.2. Since $f_{t}$ is now a convex function of $z_{t}$, the period of higher volatility correspond to higher average job finding rates. Initially – as the unemployment rate is still close to its steady-state value – this pushes the unemployment rate down. This result illustrates that matching frictions by themselves can even lead to *decreases* in the unemployment rate during periods of heightened volatility, although the value of α has to be substantially lower than values typically assumed in the literature (cf. Petrongolo and Pissarides (2001)). Of course, an anticipated increase in volatility will still have no effect when α<1/2. This is another example that illustrates how the two types of higher volatility experiments generate quite different outcomes.

### 3.4 The non-trivial implications of Nash bargaining

As shown in Section 3.1, an anticipated increase in uncertainty does lead to a reduction in the match value and a recession with Nash bargaining. So why are the results with Nash bargaining different from those with a linear wage rule? The answer actually follows quite directly from these results for the linear wage rule and the expression of the Nash-bargained wage rate, which we repeat here for convenience.

$$w_{t}^{N}=\omega \bar{x}z_{t}+\left(1-\omega \right)\chi +\omega \beta \left(1-\delta \right)E_{t}\left[\kappa \theta_{t+1}\right]$$

This expression makes clear that the wage does not only increase with the period-t benefits of not working, χ, and with current-period firm revenues, $\bar{x}z_{t}$. A higher expected value of future tightness likewise implies a higher wage rate this period.^18^

As discussed above, search frictions, and specifically the convexity of tightness, mean that the higher volatility in $J_{t}$ increases the expected values of future tightness. With Nash bargaining, this expectation translates into higher current wage rates.^19^ Higher current wages lead to a reduction in match value. The following proposition proves more formally that the match value J is concave in productivity under Nash bargaining.

Proposition 2 *Suppose that productivity is constant,*$z_{t}=z_{t+1}=\cdots =z$*, and wages are set by Nash bargaining, then*J(z)*is a strictly concave function, and*θ(z)*is a strictly convex function.*

Proof See online appendix OA. □

Intuitively, the free-entry condition together with the nonlinearity of the matching function ensures that θ is a convex function of J. Moreover, as the Nash bargained wage depends positively and linearly on tightness, the wage function is also convex in J. The concavity of J then follows from the convexity of the wage function.^20^

The concavity of $J(z_{t})$ implies that its expected value should decrease if $z_{t}$ becomes more volatile. But the story does not end here. The reduction in $J_{t}$ leads to an immediate reduction in vacancy posting, which in turn puts an immediate *downward* effect on tightness and a reduction in the job finding rate. If one considers a period with an anticipated increase in volatility that never materializes, then the expected increase in tightness due to higher volatility of $J_{t}$ will never materialize either. Consequently, there is just downward pressure on firm value, tightness, and the wage rate, consistent with the IRFs given in Fig. 1. There will be an instantaneous jump down in these variables and a gradual return towards the (stochastic) steady state. What about the total volatility effect? For tightness we have the effect that works through the wage rate, that is, tightness drops because of the expected increase in the wage rate. This channel is strongest just after the shock and then leads to a monotonically declining effect. But we also have the effect arising from the nonlinearity of the matching function, which implies that tightness is a convex function. This latter feature gives rise to an upward effect on average tightness during periods of heightened volatility. The result is a non-monotone effect that is small at first.^21^ Initially, the negative effect must dominate, but expected tightness becomes positive after two periods when it is overturned by the effect working through the nonlinearity of the matching function. The wage rate IRF leads the change in the expected value for tightness which follows directly from equation (2). The firm value is simply the mirror image of the wage rate since expected productivity actually does not change. Note that it must be the case that the total volatility IRF for tightness turns positive at some point. If it never did, then the wage response would not turn positive either, which means that firm value would not have dropped; but then tightness should not have fallen in the first place.

## 4 A search-and-matching model with option value

Section 3.2 showed that even though job creation is an irreversible investment, the standard search-and-matching (SaM) model does not have the other ingredient needed to generate an option-value channel – the mutual exclusivity of investment projects – since the choice to create a job this period does not restrict job creation in the future. In this section we propose an amended SaM model according to which elevated uncertainty does raise the value of waiting. Before specifying that model, we briefly discuss a simple experiment which demonstrates that an option-value channel is possible, in principle, simply by assuming that the mass of available entrepreneurs is *finite*. This example clarifies the crucial role of the free-entry condition in eliminating the option-value channel. It also serves as a stepping stone to understanding our proposed model.

Consider the model of Section 2 with a linear wage rule. Productivity is constant and the economy starts out in steady state. We assume that the mass of entrepreneurs, while finite, is large enough for the steady state to be unaffected. In period t, the economy encounters the following increase in anticipated volatility. Aggregate productivity in some state of period t+1 is sufficiently great for the profits associated with posting a vacancy in that state to be strictly positive. In particular, there are simply not enough unmatched entrepreneurs available in the entire economy for these profits to be exhausted due to entry. That is, the free-entry condition no longer holds in that state, as

$$h_{t+1}J_{t+1}-\kappa >0,$$

whereas it holds with equality in all other states.^22^

In period t, an idle entrepreneur is now faced with the choice of either posting a vacancy immediately, or waiting in the hope of entering when profits are strictly positive. Waiting is obviously a dominant strategy as long as profits in period t fall short of the expected profits in period t+1. Vacancies in period t therefore decline, and the hiring rate increases. This remains true until profits in period t are exactly equal to the expected profits of entry in period t+1. That is, until the arbitrage condition

$$h_{t}J_{t}-\kappa =E_{t}\left[h_{t+1}J_{t+1}-\kappa \right]>0,$$

is satisfied. Thus, the (expectation of) positive profits available in period t+1, caused by a shortage of available entrepreneurs, gives rise to positive profits in period t; profits that are generated by a rise in the hiring rate, $h_{t}$, which is accomplished through a decline in vacancies. In short, a perceived increase in future uncertainty can give rise to an option value of waiting and a decline in economic activity in the present, if that increased volatility means that the constraint on the number of available entrepreneurs is expected to be binding in some future state of the world.

While this simple extension of the baseline model is sufficient to give rise to an option-value channel, it suffers from several disadvantages. Firstly, for an option-value mechanism to operate in this environment, one had to postulate the existence of states of the world in which there is literally nobody left to create jobs, regardless of how great the associated profits are. That seems implausible. More broadly, the distribution of shocks must be such that the free-entry condition is binding in some states but not in others. That is, the presence of option-value effects is sensitive to assumptions about the size of shocks. Finally, the requirement that the constraint on the number of entrepreneurs be occasionally binding complicates the numerical analysis.

### 4.1 A model with firm heterogeneity

So what can be done? Clearly, we have to maintain the assumption of a finite mass of entrepreneurs, lest free entry drive expected profits to zero in all states of the world, eliminating the possibility of an option-value channel. At the same time, it is desirable to have an internal solution. This can be accomplished by having heterogeneity in productivity among idle entrepreneurs. The simple modification gives rise to a framework in which there are *always* idle entrepreneurs, but only *some* that find it profitable to enter the matching market. The measure that finds it profitable to do so is endogenous and time-varying. At the same time, the option value of waiting remains operative, since higher uncertainty gives entrepreneurs upward potential, whereas they are shielded from downward risk, since they can always choose not to post vacancies.^23^

#### 4.1.1 Setup

We assume that there is a finite, and constant, mass of potential entrepreneurs, Υ.^24^ We adopt the standard convention in the SaM literature according to which each entrepreneur can potentially create a one-worker firm. In every period, each unmatched entrepreneur receives an *iid* productivity draw, a, from the cumulative distribution function, F(a), with mean zero. The distribution is uniform on the interval $A=[-\bar{a},\bar{a}]$, with $\bar{a}=\sqrt{3}\sigma_{a}$, where $\sigma_{a}$ is the standard deviation of a.^25^

If an entrepreneur is successful in creating a new firm, the idiosyncratic productivity draw, a, is realized and lasts permanently throughout the match. As in the baseline model, the firm is then only dissolved by exogenous separation, which occurs at a rate δ. Entrepreneurs have finite lives, in that following separation the entrepreneur “dies” and gets replaced.^26^ If, on the other hand, the entrepreneur is unsuccessful in creating a firm, she receives a new productivity draw in the subsequent period. As a consequence, only entrepreneurs with a high enough value for a will find it worthwhile to pay the cost of posting a vacancy. Others may instead find it more beneficial to wait for the opportunity of receiving a better draw in the future. That is, there is scope for an option value of waiting, without having to rely on there being states of the world in which there are no entrepreneurs left who conceivably could post further vacancies.^27^

With idiosyncratic productivity shocks the firm value is given by^28^

(15) $$J_{t}\left(a\right)=\left(1-\omega \right)\left(\bar{x}\left(z_{t}+a\right)-\chi \right)+\beta \left(1-\delta \right)E_{t}\left[J_{t+1}\left(a\right)\right].$$

In the baseline model, the free-entry condition ensured that the value of an idle entrepreneur is always zero. In the current case, by contrast, the measure of entrepreneurs, Υ, is finite, and the value of being an idle entrepreneur prior to the revelation of the idiosyncratic draw, $J_{t}^{U}$, is non-negative and given by

(16) $$J_{t}^{U}=\int_{A}\max\left\{\beta E_{t}\left[J_{t+1}^{U}\right],h_{t}J_{t}\left(a\right)+\left(1-h_{t}\right)\beta E_{t}\left[J_{t+1}^{U}\right]-\kappa \right\}dF\left(a\right)$$

(17) $$=\int_{A}\max\left\{0,h_{t}\left(J_{t}\left(a\right)-\beta E_{t}\left[J_{t+1}^{U}\right]\right)-\kappa \right\}dF\left(a\right)+\beta E_{t}\left[J_{t+1}^{U}\right].$$

Define ${\hat{a}}_{t}$ as the productivity cut-off that renders an entrepreneur indifferent between entering or not; that is,

(18) $$h_{t}\left(J_{t}\left({\hat{a}}_{t}\right)-\beta E_{t}\left[J_{t+1}^{U}\right]\right)-\kappa =0.$$

Moreover, denote $a_{t}^{*}$ as the expected value of a conditional on a being above the cutoff level, and $p_{t}$ as the probability of such a draw. That is,

(19) $$p_{t}=1-F\left({\hat{a}}_{t}\right),a_{t}^{*}=\frac{1}{p_{t}}\int_{{\hat{a}}_{t}}^{\bar{a}}adF\left(a\right)=E_{t}\left[a|a\geq {\hat{a}}_{t}\right].$$

Then the value of an idle entrepreneur can be written as

(20) $$J_{t}^{U}=p_{t}(h_{t}\left(J_{t}\left(a_{t}^{*}\right)-\beta E_{t}\left[J_{t+1}^{U}\right]\right)-\kappa )+\beta E_{t}\left[J_{t+1}^{U}\right].$$

Lastly, the number of vacancies is given by

(21) $$v_{t}=p_{t}\left(\Upsilon -\left(1-\delta \right)n_{t-1}\right).$$

Thus, in contrast to the previous framework, the firm value is now provided by equation (15), and the free-entry condition is replaced by equation (18); the equations for $h_{t}$, $f_{t}$, $n_{t}$, as well as the exogenous processes remain the same.

Before providing a qualitative analysis of the option-value channel it is necessary to touch upon some aspects of the calibration (see Section 4.2 for additional details). In particular, our ambition is to keep the heterogenous-firm version as close as possible to the baseline, and for both frameworks to coincide – at least with respect to the key variables – at the steady state. In the baseline framework the cut-off level $\hat{a}$ is, by construction, zero. Thus, we calibrate the model such that the steady-state value of $\hat{a}$ remains at zero for any value of $\sigma_{a}$. Given the symmetry of the distribution this implies that p=0.5.^29^ Moreover, following equation (21), and imposing the steady-state values of vacancies, v, and employment, n, from the baseline model, one finds that Υ must be set as Υ=2v+(1−δ)n. Another salient implication of this choice of $\hat{a}$ is that at the steady state, the measure of idle entrepreneurs posting a vacancies is equally large as that of idle entrepreneurs that are not. Thus, the constraint on the number of entrepreneurs is unlikely to be binding even for fairly large shocks and we verified that it indeed never is for any of the numerical exercises discussed.

#### 4.1.2 An option value of waiting

The emergence of an option-value channel in this framework is intuitive and visible even in the absence of aggregate risk. We first explain how the channel emerges only due to idiosyncratic risk, and then discuss how a similar effect arises from aggregate volatility. In online appendix OC, we furthermore develop a two-period version of this model with heterogeneous productivity levels which is helpful in providing some graphical intuition as well as some analytical results.

*Idiosyncratic risk.* Suppose that there is no aggregate risk and that the cross sectional dispersion in productivity is zero; that is, $\sigma_{a}=0$. Provided that there is a sufficient mass of available entrepreneurs to exhaust all (excess) profits of entry, the first term in equation (17) must equal zero. That is,

(22) $$\max\left\{0,h(J\left(0\right)-\beta J^{U})-\kappa \right\}=0,$$

where we dropped time subscripts given the absence of aggregate uncertainty. Consequently, $J^{U}=0$, and the above equation simply replicates the free-entry condition in the standard SaM model. Thus, with $\sigma_{a}=0$, the heterogeneous-firm model nests the baseline.

Suppose instead that $\sigma_{a}>0$. If ${\hat{a}}_{t}$ were unaffected by this alteration (remaining at zero), so would the hiring rate, $h_{t}$. By contrast, the presence of cross-sectional dispersion in productivity implies that $a^{*}$ – i.e., the expected value of the idiosyncratic component conditional upon entry – must rise above zero. This means that the value of waiting, $\beta E_{t}\left[J_{t+1}^{U}\right]$, is positive as well. Consequently, an entrepreneur with a=0 now prefers to wait in the hope of getting a better draw next period. Consequently, ${\hat{a}}_{t}$ will increase until the hiring rate has dropped sufficiently so that the expected profits of vacancy posting at the new cut-off level equals the value of waiting.^30^

**Fig. 4:**
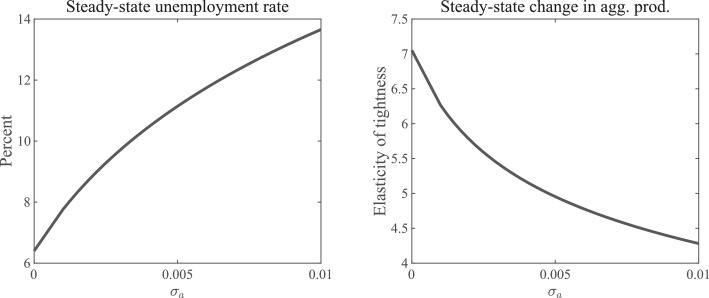
Cross-sectional dispersion and steady-state properties. *Notes:* The panels display key moment properties as a function of the amount of cross-sectional dispersion. The left panel indicates the steady-state unemployment rate, while the right panel plots the steady-state elasticity of labor market tightness with respect to aggregate productivity. All other model parameters are kept fixed and are equal to the ones given in Table 1 for the case with the linear wage rule and no cross-sectional dispersion.

Would it not be possible for changes in the hiring rate to drive the expected value of waiting to zero? No. If that were true, then the expected profits of vacancy-posting would be equal to zero for an entrepreneur with $a={\hat{a}}_{t}$. With idiosyncratic dispersion, however, this agent has some probability of receiving a draw for a in the future that exceeds ${\hat{a}}_{t}$ in which case the expected profits must be strictly positive.^31^ The more cross-sectional dispersion, as indicated by $\sigma_{a}$, the larger the difference between $\hat{a}$ and $a^{*}$; that is, the stronger the option value of waiting due to idiosyncratic risk, the lower $p_{t}$, and the higher the unemployment rate. The leftmost graph in Fig. 4 illustrates this relationship between $\sigma_{a}$ and the steady state level of the unemployment rate. As can be seen, the mechanism is powerful; an increase in the standard deviation of a from zero to 0.01 (that is, one percent of the output level without idiosyncratic dispersion) increases the steady-state unemployment rate from 6.4% to almost 14%.

**Table 1:** Calibrated parameters.

				Wage			
Parameter	Interpretation	Source/target		Nash		Linear	
β	Discount factor	Ann. interest rate of 4%		0.99		0.99	
ψ	Efficiency of matching	Unemployment rate of 6.4%		0.645		0.645	
$\bar{x}$	Markup	Markup of 11%		0.9		0.9	
δ	Separation rate	JOLTS database		0.1		0.1	
ω	Workers barg. power	Steady-state wage relation		0.5		0.915	
α	Elasticity of matching	Petrongolo and Pissarides (2001)		0.5		0.5	
κ	Vacancy posting cost	2% of steady-state output		0.14		0.14	
χ	Disutility of working	$\eta_{\theta ,z}$ equal to 7.051		0.751		0.645	
$\rho_{z}$	Persist. of agg. product.	Leduc and Liu (2016)		0.95		0.95	
$\rho_{\sigma }$	Persist. of uncertainty	Leduc and Liu (2016)		0.76		0.76	
$\sigma_{z}$	Std. agg. product. shock	Leduc and Liu (2016)		0.01		0.01	
$\sigma_{\sigma }$	Std. uncertainty shock	Leduc and Liu (2016)		0.392		0.392	

*Notes.* This table lists the parameter values of the baseline SaM model with both types of wage setting. One period in the model corresponds to one quarter. There are some slight unavoidable differences with the calibration procedure of Leduc and Liu (2016). For instance, with risk neutrality, the calibrated value of the disutility of labor parameter, χ, is slightly different than with log utility. Also, with utility linear in consumption there is no difference between disutility of labor and unemployment benefits and our χ parameter captures both. The targeted value of $\eta_{\theta ,z}$, the steady-state elasticity of labor market tightness with respect to aggregate productivity, is implied by Leduc and Liu’s (2016) calibration for the model with Nash bargaining and linear utility. Parameter values are rounded to three decimal places.

*Aggregate risk.* The presence of aggregate risk also gives rise to an option value of waiting mechanism, which operates similarly, but not identically nor independently, to the above mechanism. To understand the nuance, notice that a higher value for $z_{t+1}$ would increase the value of $J_{t+1}\left(a\right)$, while a lower value for $z_{t+1}$ would result in a decline. When wages are linear in productivity and entrepreneurs die after an exogenous separation, which is the case in this framework, the increase and decrease in $J_{t+1}$ exactly offset each other. Nevertheless there still is an option value of waiting. The reason is as follows. The increase in $z_{t+1}$ generally leads to a reduction in the cutoff value ${\hat{a}}_{t+1}$ (and, hence, in $a_{t+1}^{*}$), since *total* productivity, $z_{t+1}+{\hat{a}}_{t+1}$, will anyway increase. Similarly, the decrease in $z_{t+1}$ generally leads to an increase in ${\hat{a}}_{t+1}$ (and $a_{t+1}^{*}$). Consequently, the probability of entering and thereby benefiting from an increase in $J_{t+1}$ is higher than that of the decrease.^32^ Therefore, an anticipated increase in future aggregate volatility increases the conditional expected value of a match, $J_{t+1}\left(a_{t+1}^{*}\right)$, which thereby raises the value of waiting, $J_{t+1}^{U}$; the option-value channel materializes.

It ought to be noted that the presence of cross-section dispersion - alongside, of course, the finite measure of entrepreneurs – is necessary for this mechanism to operate *at all*.^33^ Indeed, entrepreneurs can only “benefit” from a higher match value *if* the profits of entry can be positive. Absent cross-sectional dispersion (and with a potentially infinite measure of entrepreneurs), the hiring rate would otherwise adjust to ensure that the expected profits of entry are zero in all time periods and in all states of the world, and the option-value channel would close down. The rightmost graph in Fig. 4 shows the relationship between the amount of cross-sectional dispersion, $\sigma_{a}$, and the steady-state elasticity of labor market tightness with respect to aggregate productivity. Less dispersion implies a higher elasticity, which reflects the fact that dispersion dampens the movements in the hiring rate.

### 4.2 Recalibration scheme

An insight from the previous section is that the amount of cross-sectional dispersion, $\sigma_{a}$, alters some of the key properties of the model. In particular, a higher value of $\sigma_{a}$ is associated with a higher steady-state unemployment rate for a given value of aggregate productivity. Yet, a key element of the calibration strategy of Leduc and Liu (2016) and adopted here is that the theoretical steady-state unemployment rate matches its empirical counterpart. In addition, the volatility of the hiring rate is declining in $\sigma_{a}$. In view of this, we pursue a recalibration strategy which ensures that *irrespective* of the chosen value of $\sigma_{a}$, the model economy matches key empirical targets and features a comparable degree of aggregate volatility to the baseline model of Section 2. Specifically, (i) the steady-state values of all endogenous variables are unchanged; and (ii) the steady-state elasticity of labor market tightness with respect to aggregate productivity equals the baseline.

The key parameter to obtain the latter target is χ, which controls the value of the worker’s outside option during bargaining. By choosing larger values for χ when $\sigma_{a}$ is higher, we reduce the contemporaneous surplus, $\bar{x}\left(z_{t}+a-\chi \right)$, which renders the model variables more volatile – offsetting the lower volatility implied by a wider cross-sectional distribution. Next, for the steady-state rate of unemployment to be the same as in the baseline model, the steady-state value of the cutoff level $\hat{a}$ must be equal to zero for the different values of $\sigma_{a}$ considered.^34^ The key parameter to accomplish this is the worker bargaining power, ω. When χ is increased, the share that accrues to the entrepreneur, i.e., 1−ω, must increase to ensure the same level of steady-state vacancy posting.

Lastly, the steady-state total productivity of the *average* firm is given by $\bar{z}+a^{*}$. Since $a^{*}$ increases with $\sigma_{a}$, we adjust the value of $\bar{z}$ downward to compensate for this effect. A benefit of this approach is that the steady-state value of $\left(J\left(\hat{a}\right)-\beta J^{U}\right)$, i.e., the difference in the value of a match at the cutoff relative to the value of an unmatched entrepreneur, is the same across economies.^35^ Since that term plays a key role in driving the dynamics of the model, this aspect of the recalibration procedure assists with the interpretation of the results.

Our recalibration scheme imposes a natural range for the values of $\sigma_{a}$. As $\sigma_{a}$ increases, we need to increase χ and lower ω. Above a value of $\sigma_{a}=0.003$, ω quickly approaches its natural lower bound of 0. As this is a fairly low value, we adopt it as a benchmark.^36^ Following our recalibration procedure, when $\sigma_{a}=0.003$, we set χ=0.757, ω=0.636, and $\bar{z}=0.997$. The remaining parameters are unchanged and available in Table 1, while the mass of entrepreneurs Υ given p=0.5 is equal to 1.11.

### 4.3 Numerical results

**Fig. 5:**
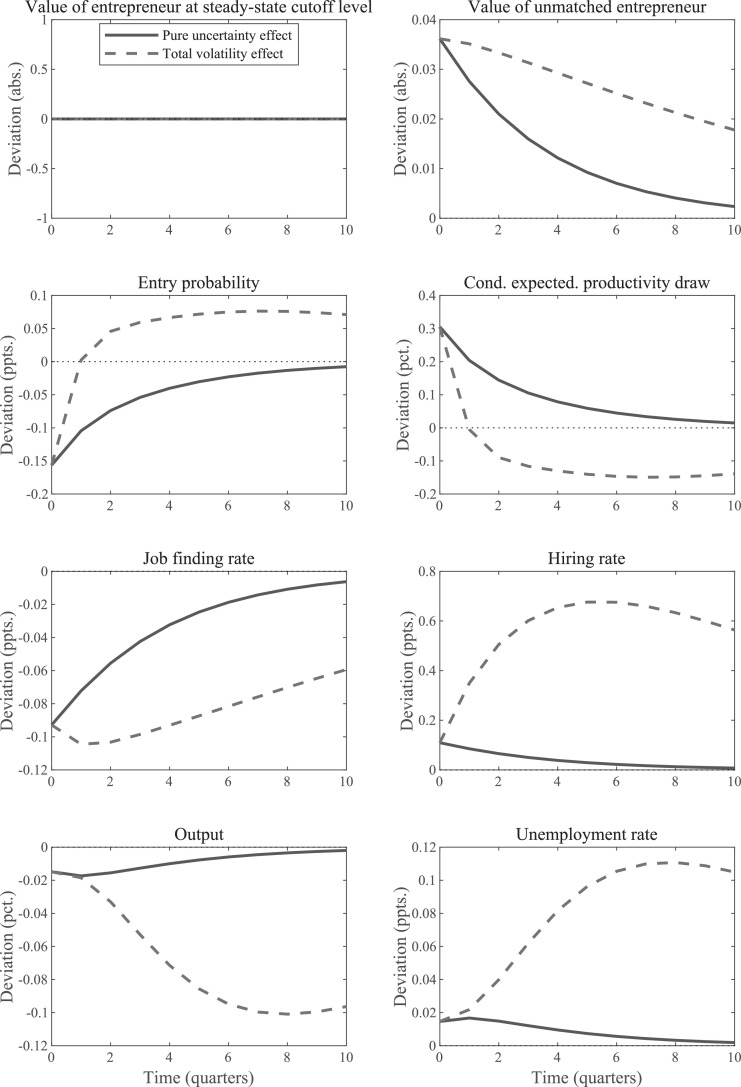
IRFs for uncertainty shock in SaM model with cross-sectional dispersion; $\sigma_{a}=0.003$. *Notes:* The “total volatility” IRFs plot the change in the period-0 expected values of the indicated variables in response to a unit-increase in $\varepsilon_{\sigma ,t}$. The “pure uncertainty” IRFs display how the economy responds when agents think volatility will increase, but the higher volatility actually never materializes.

Fig. 5 plots the IRFs for a volatility shock given $\sigma_{a}=0.003$.^37^ The following observations stand out. First, and consistent with the preceding qualitative discussion, an increase in anticipated aggregate uncertainty causes a recession even though wages are linear in productivity. The anticipation of heightened future volatility increases the value of waiting, which in turn reduces entry and vacancy-posting, lowering the job finding rate and, ultimately, pushing up the unemployment rate. Second, the total volatility effects are much larger than the pure uncertainty effects (consistent with the empirical findings of Berger et al. (2020)). This result strengthens our recommendation to consider both types of IRFs when studying the implications of time-varying volatility. Indeed, the total volatility IRFs strongly resemble those obtained in the absence of firm heterogeneity, and for similar reasons; the nonlinearities of the matching function generate a persistent rise in both the unemployment rate and the hiring rate, as discussed in Section 3.

A few subtleties are worth pointing out. For one, in the presence of idiosyncratic dispersion, aggregate output is no longer proportional to $z_{t}n_{t}$. The composition of the sample of producing firms matters, as they vary in their individual productivity levels. Specifically, changes in the number of vacancies posted occur through changes in the cutoff level, which in turn affects the average productivity of producing firms. Following a volatility shock, the value of waiting rises on impact due to anticipation effects. The associated *increase* in the average productivity level of those firms that do enter dampens, but does not overturn, the reduction in output due to the fall in employment – an effect that is absent in the model with homogeneous entrepreneurs.^38^ However, in the case of total volatility effects, this dampening effect is short-lived. The sharp rise in the unemployment rate and the associated increase in vacancy posting (through an increased entry probability) takes hold, whereupon the average productivity of entrants declines. Consequently, output not only falls because of the decline in employment, but also due to composition effects.

Moreover, uncertainty shocks have non-zero effects on the job finding rate. This result stands in contrast to the baseline model (with linear wages), according to which both the pure and the total volatility effects on the job finding rate are equal to zero in expectation when the matching elasticity, α, is equal to 0.5. Here, instead, the presence of a wait-and-see mechanism – specifically the associated reduction in the entry probability – causes the job-finding rate to decline when perceived uncertainty rises. The total volatility effect on the job-finding rate is likewise negative, larger, and more persistent. To see why, recall from the discussion in Section 3, that the hiring rate is a convex function of $z_{t}$. Equation (18) makes clear that the observed increase in the value of an unmatched entrepreneur introduces an additional positive effect on the hiring rate, and thus a negative effect on the average job finding rate.

*Quantitative comparison.* To evaluate the quantitative impact of uncertainty shocks in the current framework, we compare our results with those described in Section 3.1 for the standard SaM model with free entry and Nash bargaining (recall that this wage-setting assumption is the key reason why pure uncertainty shocks have non-zero effects in that model).

**Fig. 6:**
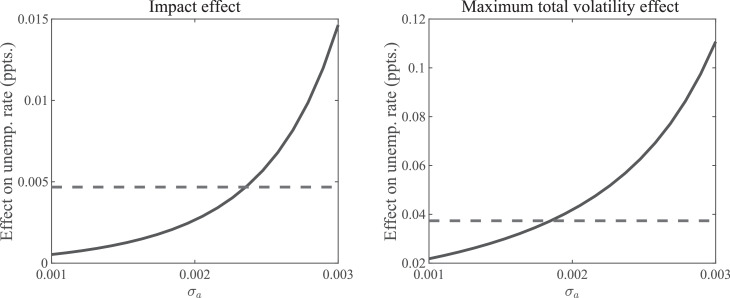
Cross-sectional dispersion and aggregate uncertainty effects. *Notes:* The panels display the initial impact and the maximum total volatility impact of a unit-increase in $\varepsilon_{\sigma ,t}$ as a function of the amount of cross-sectional dispersion, $\sigma_{a}$. Other model parameters are recalibrated to make the economies with different values of $\sigma_{a}$ comparable.

Fig. 6 illustrates the total volatility effect of an uncertainty shock on the unemployment rate, both on impact (left graph) and at the maximum along the IRF (right graph). The effect on impact is entirely due to anticipation, and the maximum total volatility effect occurs after roughly eight quarters. The horizontal lines in the two graphs indicate, for comparison purposes, the same statistics obtained in the baseline model with Nash bargaining. Recall that in the model with heterogeneity we adopted the linear wage rule and deliberately chose the mass of entrepreneurs, Υ, such that the constraint on their number is never binding. Hence, with barely any cross-sectional dispersion, there should be no quantitatively significant anticipation effects due to a volatility shock. The figure reveals that, indeed, for very small values of $\sigma_{a}$, we are essentially back to the model of Section 2 with linear wages.

Nonetheless, even with still relatively little cross-sectional dispersion, volatility shocks can generate a substantial effect on unemployment. Thus, a value of $\sigma_{a}$ equal to 0.003 implies that the entrepreneur with the most productive draw for a is just one percent more productive than the entrepreneur with the least productive draw. In spite of that, both the initial pure uncertainty effect as well as the maximum total volatility effect are more than double than what is generated in the baseline model with Nash bargaining.

*Robustness checks.* In online appendix OF, we discuss the results of several robustness exercises. Most importantly, our baseline specification of the model assumes that an entrepreneur can post only one vacancy and then creates a job with probability $h_{t}$ (“stochastic hiring”). An alternative is to suppose that the entrepreneur posts $1/h_{t}$ vacancies and then creates one job with certainty (“non-stochastic hiring”). In the standard SaM model with risk-neutral entrepreneurs, these two options generate the exact same model properties. In our modified framework, entrepreneurs are also risk neutral and the two different specifications imply the same qualitative properties. Quantitatively, however, when there is both aggregate and idiosyncratic uncertainty, a model with non-stochastic hiring generates a substantially stronger option-value effect due to elevated volatility than implied by our baseline specification.

## 5 Concluding remarks

The option value of waiting to invest in the presence of uncertainty strikes many as a plausible mechanism to rationalize the empirical finding that elevated uncertainty negatively impacts economic activity. Moreover, the popularity of the search-and-matching (SaM) literature underscores the usefulness of modeling job creation as an investment. Yet, we showed that the usual assumption in that literature of there being a “potentially infinite number” of entrepreneurs to take advantage of opportunities in the matching market eliminates any grounds for wait-and-see behavior. The standard SaM model, therefore, cannot be used to rationalize the effects of uncertainty shocks in terms of an option-value channel. If, on the other hand, there is a limit on the number of potential entrepreneurs and they vary in their idiosyncratic productivity levels – two modifications that are both plausible and can be introduced into the model in a tractable manner – the model properties completely change. In particular, an increase in perceived volatility then does indeed robustly increase the option value of waiting, causing a reduction in job creation and higher unemployment.

## Declaration of Competing Interest

None.

## Appendix A Supplementary materials

Supplementary Data S1 Supplementary Raw Research Data. This is open data under the CC BY license http://creativecommons.org/licenses/by/4.0/ Supplementary Data S1

## Footnotes

1 For excellent surveys of the literature, see Bloom (2014) and Fernández-Villaverde and Guerrón-Quintana (2020). Also see, however, Berger et al. (2020) for an argument that it is realized volatility, rather than the perception of a more uncertain future, that matters for adverse economic consequences.

2 Key references on the empirical effects of uncertainty are Jurado et al. (2015) and Baker et al. (2016). Regarding the effects of uncertainty on (un-)employment specifically, see Caggiano et al. (2014) and Leduc and Liu (2016). Freund and Rendahl (2020, Appendix A) document that elevated uncertainty causes not only the unemployment rate but also vacancies to respond in a manner that is consistent with the predictions of the search-and-matching frameworks explored in this paper.

3 The decline in demand for consumption goods induced by elevated uncertainty leads to reduced economic activity when prices are sticky (cf. Basu and Bundick (2017)). In fact, an additional contribution of Leduc and Liu (2016) is to show the importance of embedding their SaM model into a New Keynesian framework with price rigidities. Together with nonlinear household utility, these give rise to an aggregate demand channel which ensures that the model can generate quantitatively substantial effects following uncertainty shocks. To make our analysis more transparent, we abstract from price stickiness and associated demand effects.

4 We share with Coles and Kelishomi (2018) and Leduc and Liu (2020b) an emphasis on the restrictive nature of the standard free-entry condition in the SaM model. We differ from these papers in that we concentrate on the implications of free entry for the effects of uncertainty shocks and, in particular, the presence or absence of option-value effects.

5 For a related approach, see Riegler (2019).

6 While in the main text we interpret our model as describing one-worker firms, in online appendix OE we show that there exists an equivalent representative multi-worker firm representation – just as it does for the standard SaM model.

7 Freund and Rendahl (2020) explain how risk aversion can lead to a larger impact of uncertainty shocks through changes in the required risk premium. We further discuss the role of risk aversion in online appendix OB.

8 In the model of Leduc and Liu (2016), final good producers earn a markup, which means that the relative price of intermediate goods, $\bar{x}$, is less than one. In this version with flexible prices, the level of the markup is not relevant, but we retain the parameter $\bar{x}$, so that our calibration is as close as possible to Leduc and Liu’s (2016).

9 See online appendix OA.1 for a derivation.

10 The starting point does potentially matter in a nonlinear model. Here we follow the literature and suppose that the shock occurs after a long period during which no shocks have materialized at all (cf. Born and Pfeifer (2014)). The total volatility IRFs are calculated using the technique of Andreasen et al. (2018).

11 This relationship is also important to understand first-order moment “news” shocks, that is, how the economy responds in period t to news that productivity will be higher in the future requires understanding how the economy is expected to respond to such an increase in productivity.

12 By construction, the two IRFs take on the same value in the period when the shock occurs, since our timing assumption implies that uncertainty shocks have no effect in that period. Thus, the impact-period responses for both types of IRFs are purely based on expectations on what will happen and those expectations are the same.

13 What matters for economic activity in a standard SaM model with exogenous separation are the expected profits from posting a vacancy, not those of an existing match. Thus, we consider the decision of an unmatched entrepreneur.

14 Irreversibility refers to the posting costs. Here, we have assumed that the entrepreneur who invests in period 1 will have some positive cash flow in period 2 for sure ($R_{2}\geq 0$). That is, there is no incentive to end the relationship early endogenously. But $R_{2}$ could be negative, for example, with sticky wages. Allowing for endogenous discontinuation means that the net present value of the surplus accruing to the entrepreneur investing in period 1 is given by

$$-\kappa +h_{1}R_{1}+\beta E\left[\max\left\{R_{2},0\right\}\right]=-\kappa +h_{1}J_{1}=0.$$

The convexity introduced by endogenous job destruction implies that an increase in anticipated uncertainty would *raise* the value of $J_{1}$, which in turn would lead to an increase in vacancies in period 1. That is, the outcome is the opposite of that predicted by an option value of waiting mechanism.

15 This linear specification can be motivated by an alternating-offers game (Freund, Rendahl, 2020, Hall, Milgrom, 2008). A key aspect of this game is that separation is not a credible threat. Consequently, agreement is reached within the period and market tightness does not affect the outcome. As long as agreement has not been reached, the worker is not working. The parameter χ captures the utility of not working during the negotiations. This wage coincides exactly with that of Jung and Kuester (2011), in which the Nash product, ${\left(w_{t}-\chi \right)}^{\omega }{\left(\bar{x}z_{t}-w_{t}\right)}^{1-\omega }$, is maximized.

16 The parameters of the version with linear wages are chosen to make it comparable to that with Nash bargaining. Specifically, the outside option χ is set such that the elasticity of labor market tightness with respect to productivity is unchanged relative to Nash bargaining. To this end, we exploit the close relationship between that elasticity and the fundamental surplus, $\bar{x}\bar{z}-\chi$, as defined by Ljungqvist and Sargent (2017). Given the other parameters, the bargaining weight ω is then pinned down by the steady-state version of equation (13). Parameter values are given in Table 1.

17 See Hairault et al. (2010) and Jung and Kuester (2011).

18 To gain intuition for why this is the case, notice that using the free-entry condition, we can write

$$\kappa \theta_{t+1}=\kappa \frac{v_{t+1}}{u_{t+1}^{s}}=\kappa \frac{f_{t+1}}{h_{t+1}}=f_{t+1}J_{t+1}.$$

That is, what matters for wage setting in terms of forward-looking behavior is the expected value of the product of next period’s job finding rate and next period’s firm value.

19 In online appendix OB we show that if households are risk averse, then additional interaction effects come into play. In particular, whenever the marginal utility of consumption is elevated, this lowers the Nash-bargained wage rate.

20 The above reasoning relies on the properties of J rather than productivity z. To complete the argument, notice that tightness will always be a convex function of z*unless*J is sufficiently concave. However, if this is the case, labor market tightness as well as wages are concave functions of z. Since w enters the match value negatively, this would imply that J must be convex, which is a contradiction. Thus J is concave in z. See Section 3.3 for a discussion of a similar relationship between θ and z even when J is a linear function of z.

21 If the shock occurs in period τ, then $z_{\tau +1}$ will be more volatile because $\varepsilon_{z,\tau +1}$ is more volatile. But $z_{\tau +2}$ will be more volatile because $\varepsilon_{z,\tau +2}$*and*$z_{\tau +1}$ will be more volatile.

22 Note, in particular, that the prospect of a large *fall* in aggregate productivity leaves expected profits unaffected, since such a shock lead to an increase in the slack of the constraint on the available number of entrepreneurs.

23 The models of Coles and Kelishomi (2018) and Leduc and Liu (2020b), referred to already in footnote 4, likewise feature firm heterogeneity and an imperfectly elastic entry margin. Nonetheless, these models rule out an option-value channel by assumption. The reason is that in their setup, each one of a fixed mass of entrepreneurs can run several, independent business opportunities, and entering today does not preclude entering again next period. That is, mutual exclusivity does not obtain.

24 Section OD.2 in the online appendix considers a more general version in which Υ is allowed to be time-varying. It nests both the model discussed here and the standard SaM model with free entry.

25 Results with a Normal distribution are similar and are reported in online appendix OF.2.

26 The match value does not feature a term related to the value of waiting as entrepreneurs die upon separation. This assumption is made to allow for a transparent analysis. When entrepreneurs live for ever, they would receive a new draw of a after separation and – just like entrepreneurs who wait – benefit from increased uncertainty although only at some future date. Online appendix OD.1 provides an alternative setup with infinitely-lived entrepreneurs.

27 For the standard SaM model, there exists an equivalent representation with a representative n-worker firm. Online appendix OE derives such a representation for a generalized version of our model with idiosyncratic productivity dispersion. Therefore, both the standard and our alternative model can be interpreted as explaining changes in employment either through the extensive margin (changes in firm creation), the intensive margin (changes in firm size), or both.

28 The notation is somewhat simplified in that it does not specify that a is the draw that the entrepreneur received in the period the match was created. Over time, firm level productivity, $z_{t}+a$, only varies with aggregate productivity, $z_{t}$. We also do not add a subscript to indicate that the level of a is firm-specific.

29 Online appendix OF.4 provides the results when this fraction is equal to 0.2 instead.

30 It is of course essential that entrepreneurs have the option to *not* post a vacancy in the future. That is, expected profits are always bounded below at zero. This leads to a convex payoff function and Jensen’s inequality then implies that uncertainty raises expected values.

31 With idiosyncratic dispersion, $a_{t}^{*}$ could be equal to ${\hat{a}}_{t}$, but only if ${\hat{a}}_{t}$ is at the upper bound of the distribution, that is, when nobody would want to post vacancies. This does not happen for any of our parameterizations, because changes in matching probabilities always ensure an interior solution for ${\hat{a}}_{t}$.

32 Phrased in another way, the higher $J_{t+1}$ is multiplied by a higher value of $p_{t+1}$ than the lower $J_{t+1}$. See online appendix OC for an intuitive and graphical exposition using a 2-period version of the model.

33 That is, unless we rely on a shock distribution that render the free-entry condition occasionally binding.

34 Recall that Υ is set such that the fraction of entrepreneurs that enters the matching market is 1/2 in the economy without cross-sectional dispersion.

35 The calibration strategy involves setting the fraction of output spent on vacancy posting costs in steady-state, $\kappa v/(\bar{z}a^{*}n)$, equal to 2%. The adjustment of $\bar{z}$ ensures that κ is the same across economies, which together with the fact that h is calibrated to be the same across economies means that $J\left(\hat{a}\right)-\beta J^{U}$ is the same across economies.

36 This amount of idiosyncratic dispersion is small relative to the degree of cross-sectional productivity dispersion observed in the real world. See, for example, Sterk et al. (2020). It is not surprising that our framework with ex-ante identical entrepreneurs cannot generate the observed differences which are likely to arise from numerous factors besides those present in a simplified model as we consider here.

37 See online appendix OF.1 for the same set of IRFs given $\sigma_{a}=0.001$. Additionally, Fig. 6 plots the impact and maximum total volatility effect on the unemployment rate, specifically, as a function of $\sigma_{a}$.

38 Figure OF.3 in the online appendix illustrates that this effect can, in principle, lead to a small initial increase in output when $\sigma_{a}=0.001$.
