# Supplementary material for: Volatile hiring: uncertainty in search and matching models
Source: J Monet Econ. 2021 Oct;123:1–18. doi: 10.1016/j.jmoneco.2021.07.008 (PMC8547261; doi:10.1016/j.jmoneco.2021.07.008)
Supplement: Supplementary Data S1 — Supplementary Raw Research Data. This is open data under the CC BY license http://creativecommons.org/licenses/by/4.0/ [file mmc1.pdf]

Online Appendix for:
"Volatile Hiring: Uncertainty in Search and Matching Models"*

Wouter Den Haan
London School of Economics, CEPR and CFM

Lukas B. Freund
University of Cambridge

Pontus Rendahl
Copenhagen Business School, CEPR and CFM

July 2021

This appendix contains supplemental material for the article "Volatile Hiring: Uncertainty in Search and Matching Models."

Any references to equations, figures, tables or sections that are not preceded by a capital letter refer to the main article.

**E-mail*: Den Haan: w.denhaan@lse.ac.uk; Freund: lukas.beat.freund@gmail.com; Rendahl: pontus.rendahl@gmail.com.

Appendix OA Derivations and proofs

OA.1 Derivation of Nash-bargained wage

Let V_t and U_t denote the value of an employed and an unemployed worker, respectively. That is,

$$V_t = w_t + \beta E_t \left[(1-\delta+\delta f_{t+1}) V_{t+1} + \delta(1-f_{t+1}) U_{t+1} \right],$$
$$U_t = \chi + \beta E_t \left[f_{t+1} V_{t+1} + (1-f_{t+1}) U_{t+1} \right].$$

Thus, the surplus to the household of accepting a job is given by

$$S_t = V_t - U_t = w_t - \chi + \beta E_t \left[(1-\delta)(1-f_{t+1}) S_{t+1} \right]. \tag{OA.1}$$

Similarly, the surplus to the firm of hiring a worker is simply the firm value, J_t, (which is repeated for convenience)

$$J_t = \bar{x} z_t - w_t + \beta(1-\delta) E_t [J_{t+1}]. \tag{OA.2}$$

Nash bargaining sets the wage, w_t, to maximize the Nash product such that

$$w_t = \arg\max \{ J_t^{1-\omega} S_t^{\omega} \},$$

where ω represents the bargaining power of the worker. The first order condition is given by

$$(1-\omega) S_t = J_t \omega. \tag{OA.3}$$

Using the first order condition in equation (OA.3) together with equations (OA.1) and (OA.2)

gives

$$(1-\omega)(w_t-\chi)+\beta(1-\delta)\omega E_t[(1-f_{t+1})J_{t+1}] = \omega(\bar{x}z_t-w_t)+\beta(1-\delta)\omega E_t[J_{t+1}].$$

Solving this equation for w_t gives the following expression for the Nash-bargained wage rate:

$$w_t^N = \omega\bar{x}z_t+(1-\omega)\chi+\omega\beta(1-\delta)E_t[f_{t+1}J_{t+1}].$$

Using the free-entry condition, $\kappa = h_t J_t$, and the relationship between the hiring rate, the job finding rate, and tightness, $f_t = h_t\theta_t$, we get the expression which relates the wage rate to expected tightness.

$$w_t^N = \omega\bar{x}z_t+(1-\omega)\chi+\omega\beta(1-\delta)\kappa E_t[\theta_{t+1}].$$

OA.2 Proof of Proposition 1

To derive Proposition 1, we substitute the linear wage rule given in equation (13) into the firm value equation in (1) and iterate forward.[OA.1] Thus,

$$\begin{aligned} J_t &= \bar{x}z_t - w_t + \beta(1-\delta)E_t J_{t+1} \\ &= E_t\Sigma_{j=0}^{\infty}\beta^j(1-\delta)^j(1-\omega)(\bar{x}z_{t+j}-\chi). \end{aligned}$$

[OA.1] We rule out exploding paths, such that

$$\lim_{j\to\infty}[\beta(1-\delta)]^j E_t[J_{t+j}] = 0, \quad t = 0,1,\ldots$$

Next, use the law of motion for productivity (5).

$$J_t = -\frac{(1-\omega)\chi}{1-\beta(1-\delta)} + (1-\omega)\bar{x}z_t + \beta(1-\delta)(1-\omega)[\bar{x}((1-\rho_z)+\rho_z z_t)]$$
$$+\beta^2(1-\delta)^2(1-\omega)[\bar{x}((1-\rho_z)+\rho_z(1-\rho_z)+\rho_z^2 z_t)]$$
$$+\beta^3(1-\delta)^3(1-\omega)[\bar{x}((1-\rho_z)+\rho_z(1-\rho_z)+\rho_z^2(1-\rho_z)+\rho_z^3 z_t)]+\ldots$$

Simplifying,

$$J_t = -\frac{(1-\omega)\chi}{1-\beta(1-\delta)} + \frac{(1-\omega)\bar{x}z_t}{1-\beta(1-\delta)\rho_z} + \frac{\frac{\beta(1-\delta)(1-\omega)(1-\rho_z)\bar{x}}{1-\beta(1-\delta)}}{1-\beta(1-\delta)\rho_z},$$

and collect terms,

$$J_t = -\frac{(1-\omega)\chi}{1-\beta(1-\delta)} + \frac{(1-\omega)\bar{x}z_t}{1-\beta(1-\delta)\rho_z} + \frac{\beta(1-\delta)(1-\omega)(1-\rho_z)\bar{x}}{(1-\beta(1-\delta))(1-\beta(1-\delta)\rho_z)}.$$

This final line corresponds to equation (14). □

OA.3 Proof of Proposition 2

The firm value is in this case given by

$$J(z) = \frac{(1-\omega)(xz-\zeta)}{1-\beta(1-\delta)} - \frac{\beta\omega\kappa\theta(z)}{1-\beta(1-\delta)}.$$

Suppose that $J(z)$ is (weakly) convex in the vicinity of some $z > 0$. That is

$$tJ(z_1) + (1-t)J(z_2) \geq J(z),$$

for some $z_1 > 0$ and $z_2 > 0$ and any $t \in (0,1)$ such that $z = tz_1 + (1-t)z_2$. Then by definition

$$\frac{(1-\omega)(xz-\zeta)}{1-\beta(1-\delta)} - \frac{\beta\omega\kappa(t\theta(z_1)+(1-t)\theta(z_2))}{1-\beta(1-\delta)} \geq \frac{(1-\omega)(xz-\zeta)}{1-\beta(1-\delta)} - \frac{\beta\omega\kappa\theta(z)}{1-\beta(1-\delta)},$$

or simply

$$(t\theta(z_1) + (1-t)\theta(z_2)) \leq \theta(z).$$

That is, $\theta(z)$ must be weakly concave in the vicinity of z.

The free-entry condition implies that

$$\theta(z) = \left(\frac{\psi}{\kappa} J(z)\right)^{\frac{1}{\alpha}},$$

which implies that $\theta(z)$ is a strictly convex function in the vicinity of z. As this is a contradiction, $J(z)$ must be strictly concave for all $z > 0$, which implies that $\theta(z)$ must be strictly convex for all $z > 0$. □

Appendix OB Risk aversion in the standard model

Our main analysis assumes that the representative household is risk neutral. This assumption carried the benefit of isolating the analysis to option-value considerations. Allowing for risk aversion introduces a number of complexities in the form of additional transmission channels and interaction effects. In this section, we give an indication of what direction they go. We highlight, in particular, that risk aversion alters the predictions of the canonical search-and-matching (SaM) model for the effects of uncertainty shocks on economic activity in two primary ways. First, the interaction of investor risk aversion and search-frictions in the pricing of firm equity gives rise to non-zero, adverse, pure uncertainty effects; even when the wage function is linear. This stands in marked contrast to the risk-neutral benchmark. Second, if households are risk averse, regular Nash bargaining over wages may dampen this uncertainty-induced recession.

To derive these results, we proceed in two steps. We start by assuming that wages are a linear function of current productivity, as in equation (13). And later we will add Nash bargaining to the analysis. In the main text, which assumed risk neutrality, we emphasized that under this specification, the stream of expected dividends from a match is unaffected by an increase in uncertainty (cf. Proposition 1). But risk aversion makes the representative household value any given dividend stream differently when uncertainty increases. The equation pinning down the period-t firm value incorporates stochastic discounting of the continuation value in the expectation term is

$$J_t = \bar{x}z_t - w_t + (1-\delta)E_t\left[\frac{u'(c_{t+1})}{u'(c_t)}J_{t+1}\right]. \tag{OB.1}$$

Moreover, as in Leduc and Liu (2016), households can save not only in form of equity, but also risk-free government bonds that pay a gross real interest rate R_t. As such, the system of equations is augmented by a standard bond Euler equation.[OB.1]

Freund and Rendahl (2020) explore this model with linear wages in depth, considering also

[OB.1] However, as prices are flexible, this augmentation merely prices bonds, and does not alter any economic quantities.

the role of nominal rigidities and shedding light on the simultaneous operation of supply and demand channels through which uncertainty shocks then affect economic activity. Here we briefly summarize their results for the transmission of uncertainty shocks in a setting with flexible prices.

Figure OB.1: Pure uncertainty IRFs in SaM model with risk aversion and linear wage rule

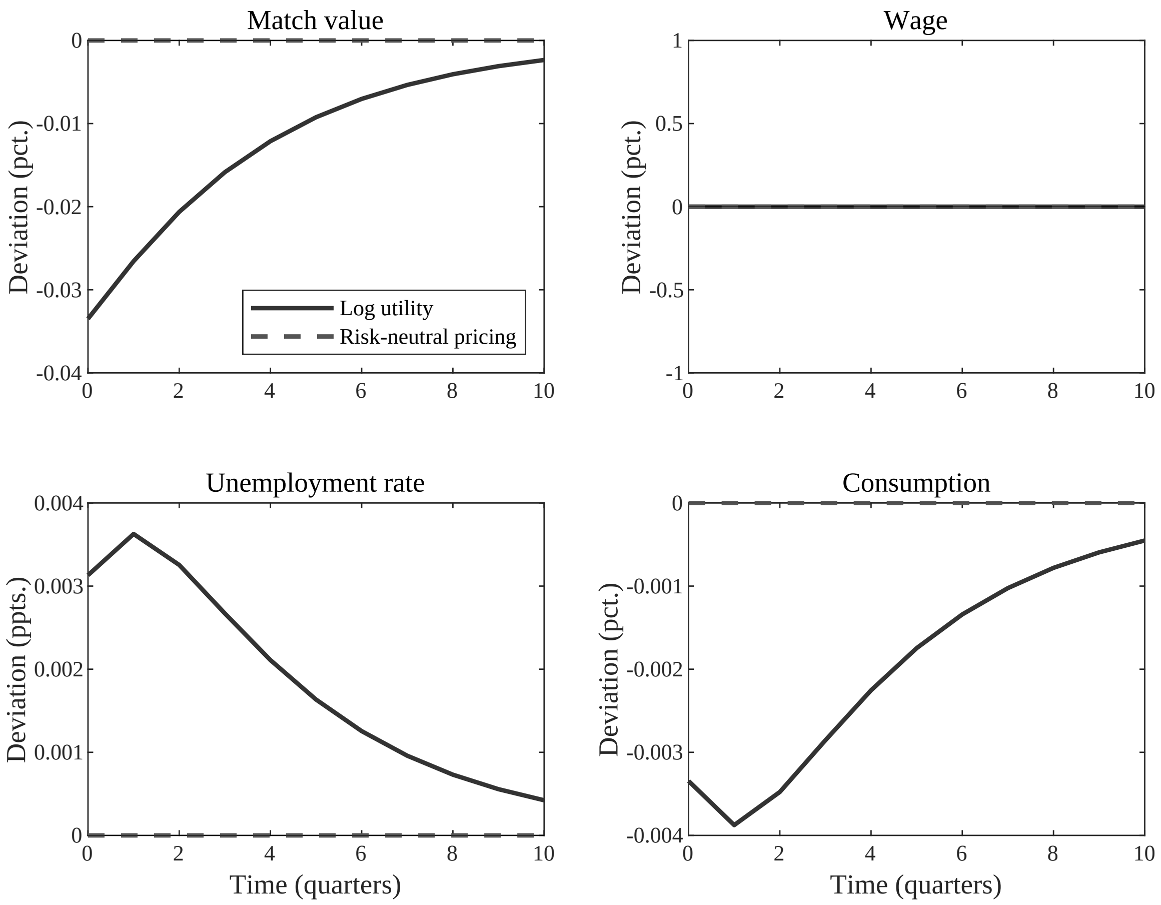

Notes: The figure shows the "pure uncertainty" IRFs to a unit-increase in $\varepsilon_{\sigma,t}$ for the SaM model with a linear wage rule and allowing for risk aversion. These IRFs display how the economy responds when agents think volatility will increase, but the higher volatility actually never materializes.

The solid line in figure OB.1 indicates the pure uncertainty IRF for the firm value, wages, unemployment, and consumption, assuming a logarithmic utility function. As can be seen, the increase in uncertainty causes the firm value to fall. A lower firm value means that the incentives to post vacancies are weakened, and the job-finding rate declines. Unemployment consequently rises, while output and consumption contract. Thus, while search frictions *by themselves* are insufficient

to raise the unemployment in response to an increase in perceived uncertainty – as visualized by the the dashed line, which describes the pure uncertainty effect in the risk-neutral case – their *interaction with risk aversion* means that a rise in uncertainty lowers economic activity even when prices are flexible and the wage function is linear.

Three distinct mechanisms underpin this result. Two of them are expansionary, but they are dominated by a contractionary force which operates through the risk premium.[OB.2] On the one hand, two mechanisms trigger a rise in households' desire to save that leads them to value all assets, including the risky equity of intermediate goods firms, more highly when faced with a more uncertain future. The first mechanism is linked to the usual prudence motive associated with the marginal utility of consumption being convex; by Jensen's inequality, the perception of greater future volatility pushes up $E_t[u'(c_{t+1})]$. The starting point for the second expansionary mechanism is that, as described in section 3.3, search frictions mean that average unemployment is higher in periods of heightened volatility. Households *anticipating* the future to be more volatile, therefore, also *expect* average unemployment to be higher. If households aim to smooth consumption over time, this expectation reinforces their desire to save in form of both bonds and equity rather than consume in the present. At the same time, however, increased uncertainty about the future generates a stronger negative comovement between the marginal utility of consumption and the equity value; low payoffs are expected for precisely those periods where consumption will be low and, hence, when dividend income would be more valuable (and vice versa). This negative comovement is captured by a rise in the required risk premium, causing a fall in the firm value. In summary, therefore, a rise in uncertainty lowers economic activity when households are risk averse and when the wage is unresponsive to expected movements in either labor market tightness or marginal utility. The reason is that households anticipating greater future volatility require a larger risk premium to compensate them for holding the equity of firms with long-term employment relationships. More costly equity acts to suppress hiring activity.

[OB.2]In principle, the net effect of these mechanisms is *ex ante* ambiguous. Freund and Rendahl (2020) underscore that the risk premium can be large and volatile in the SaM setting, the reason being that hiring a worker is akin to investing in risky assets with long-duration payoffs.

Next, suppose that wages are not a linear function of productivity but, instead, are determined by Nash bargaining. As such, the theoretical environment we consider here corresponds to the flexible-price version of Leduc and Liu (2016, see their section 4.2.1).[OB.3] We follow Leduc and Liu (2016) in supposing that the worker's reservation value consists of a combination of unemployment benefits, ϕ, and disutility of supplying labor, χ (or, equivalently, a linear utility parameter for leisure).[OB.4] Extending equation (2), the wage is pinned down as

$$w_t^N = (1-\omega)\left(\phi + \frac{\chi}{u'(c_t)}\right) + \omega\left(\bar{x}z_t + \beta(1-\delta)\kappa E_t\left[\frac{u'(c_{t+1})}{u'(c_t)}\frac{v_{t+1}}{u_{t+1}^s}\right]\right), \tag{OB.2}$$

Relative to the risk-neutral Nash wage, household risk aversion shows up in two ways in equation (OB.2). First, the utility of leisure accrues to the worker's surplus when bargaining, but in consumption units (which is then suitably shared between the firm and the worker according to their respective bargaining power). Thus, the parameter appears in the wage equation divided by the marginal utility of consumption. Second, next-period labor market tightness is discounted with the marginal rate of substitution.

Figure OB.2 reports the effects of an increase in anticipated volatility under four wage-setting specifications: (a.) the benchmark case with linear wages (solid line); (b.) "regular" Nash bargaining (following equation (OB.2), dashed line); (c.) the wage materializing under Nash bargaining with risk neutrality (following equation (2); dashed-dotted line); and (d.) the wage materializing under Nash bargaining when the money-metric value of the utility of leisure is held at its steady-state value (dotted line). For (c.), we only assume risk neutrality in deriving the wage equation.

The figure makes clear that Nash bargaining can either dampen ("regular") or magnify (risk-neutral Nash wages) the IRFs. The difference between (a.) and (c.) is essentially the "Nash-wage

[OB.3] Different from Leduc and Liu (2016), we abstract from extrinsic wage rigidity to preserve greater transparency. On the same grounds, we also continue to assume that resources expended on vacancy posting are rebated to the household. Quantitatively, the numbers we report for the case of Nash bargaining are therefore most comparable, but do not exactly coincide with, the results reported in their online appendix, specifically the dotted line in their Figure A6.

[OB.4] As far as the calibration of the model is concerned, we also stay as close as possible to Leduc and Liu (2016). As such, under Nash bargaining we take the flow benefits of unemployment, ϕ, to be equal to 0.25, while the disutility of working, χ, is set equal to 0.5348. Given a steady-state value of consumption $\bar{c} = \bar{n} = 0.9360$, the monetary value of $(\phi + \chi/u'(c_t))$ in steady-state monetary terms is therefore 0.751, just as in the risk-neutral setting. The steady-state elasticity of labor market tightness with respect to productivity thus remains unchanged.

Figure OB.2: Pure uncertainty IRFs in SaM model with risk aversion

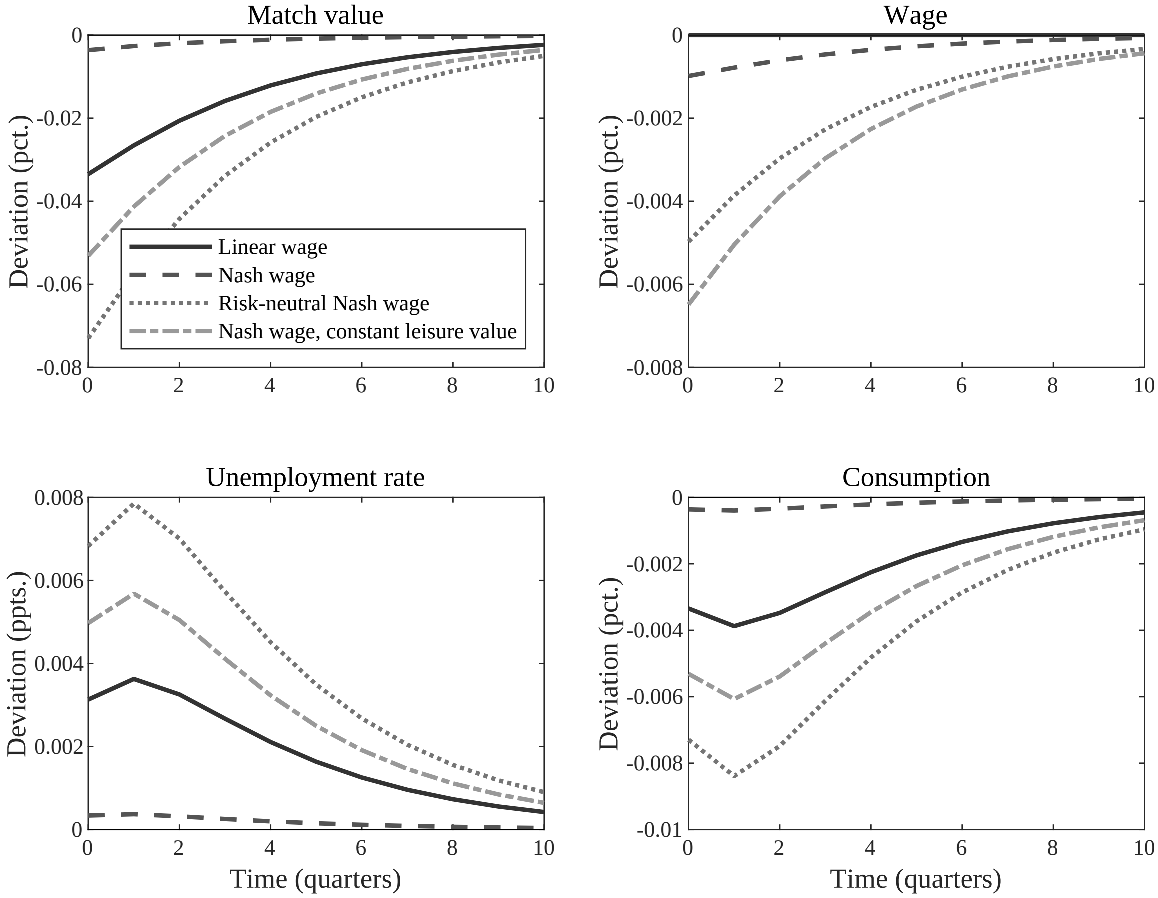

Notes: The figure shows the "pure uncertainty" IRFs to a unit-increase in $\varepsilon_{\sigma,t}$ for the SaM model, allowing for risk aversion and considering alternative wage specifications.

channel" explored in the main text, that is, the interaction of higher expected future labor market tightness and Nash bargaining adds to the recessionary effect of a rise in the risk premium.

The possibility of a dampening effect, on the other hand, is due to the fact that whenever the household's marginal utility of consumption is elevated relative to the steady state, this lowers the wage rate. For when a recession materializes, consumption falls and the marginal utility of consumption increases. Thus, the monetary value of leisure declines, which, ceteris paribus, means that the wage is permitted to fall more than under risk neutrality. With more wage flexibility, there are smaller movements in asset prices, and the contraction is less pronounced (which, seemingly contradictory, leads to a smaller decline in wages). What is more, when uncertainty is *expected* to be

high, the same channels that give rise to a precautionary motive raise the expected marginal utility of consumption. By the preceding logic, this mechanism lowers the expected wage, and, hence, raises expected future dividends. It therefore exerts upward pressure on the firm value, J_t, which, after all, is simply the present discounted value of dividends. Because J_t is elevated relative to the risk-neutral benchmark, vacancy creation and employment are boosted. As a result, consumption is actually higher and the marginal utility consequently lower, which raises the realized wage, other things equal.[OB.5] The dotted line in Figure OB.2 (case d.) shows this by adding a Nash bargaining solution which holds the utility component of leisure fixed in money terms over the cycle. Compared to the baseline with Nash wages, the effect of a rise in uncertainty is worse than under regular Nash bargaining or, indeed, the linear wage specification.

A final nuance arises from the fact that under regular Nash bargaining with risk aversion, the stochastic discount factor is involved in determining the worker's surplus. In terms of equation (OB.2), uncertainty shocks also propagate through the product term inside the conditional expectation involving the ratio of marginal utilities and next-period labor market tightness. In Figure OB.2, comparing the dashed-dotted line – corresponding to them model with wages determined by Nash bargaining under risk neutrality – and the dotted line – where only the money-metric value of leisure is held constant – reveals that the presence of this term exerts a positive effect on the response of the firm value to a rise in perceived future volatility. The opposite is true for the wage response. To explain why this is the case, note that similar to the precautionary savings effects discussed above, a first consequence of agents anticipating persistently higher future volatility is to put upward pressure on the expected future marginal utility, which in this instance exerts *upward* pressure on the expected future wage. Intuitively, any benefit workers derive from a tight labor market in the future counts for more. This effect lowers expected future dividends and hence the current match value, setting off the by now familiar chain of events that ends up lowering realized labor market tightness and higher unemployment. On the other hand, though, business cycle fluctuations are associated with a negative covariance between the stochastic discount factor and labor market tightness. As

[OB.5] When real wages are assumed to be extrinsically rigid, as in Leduc and Liu (2016), these effects are attenuated.

greater volatility strengthens this negative co-movement, anticipation thereof acts to *lower* the wage in both present and future, which incentivizes job creation. In principle, the net effect of these two channels is ambiguous, but the figure reveals that under the benchmark calibration it, too, serves to dampen the adverse impact of the uncertainty shock on unemployment relative to the case of risk-neutral Nash bargaining.

In summary, when households are risk averse, a rise in uncertainty may push the economy into a recession even when wages, and therefore also dividends, are linear in productivity. Nash bargaining over wages, on the other hand, may exert a dampening effect under risk aversion, provided the workers' total flow benefits from unemployment are sensitive to variations in marginal utility.

Appendix OC Two-period model with heterogeneity

This section presents and examines a two-period variant of the search-and-matching model developed in section 4.1, which features an option-value effect of waiting due to uncertainty about productivity at both the firm-specific and the aggregate levels. This two-period model affords an intuitive, graphical exposition of the key mechanisms as well as analytical results. We first suppose that the probability of an entrepreneur successfully hiring a worker upon posting a vacancy is fixed at some level $\bar{h}$. This case reveals the conditions under which an option value of waiting exists, and the underlying intuition, in a particularly transparent way. Thereafter, we consider the case in which congestion on the matching market renders the hiring rate endogenous, as in the full model.

OC.1 Constant hiring rate

Environment. There are two periods and no discounting. Entrepreneurs can produce either in period 1 or in period 2, but not both.[OC.1] That is, the separation rate is equal to 1. Production is given by $z_t + a_{i,t}$, where a_i is distributed over the interval $[-\bar{a}, +\bar{a}]$ according to the distribution F. Aggregate productivity in the first period is $z_1 = 1$, while z_2 is equal to either $1+\Delta$ ('expansion') or $1-\Delta$ ('recession'), with equal probabilities. The parameter Δ is, thus, a measure of aggregate uncertainty. Workers have no bargaining power and their outside option is zero, such that the profits of a matched entrepreneur are equal to the full value of output net of hiring costs. With the price of such output normalized to unity, and without loss of generality, let the cost of starting a firm, κ, be equal to the fixed hiring probability $\bar{h}$, so that an entrepreneur with draw $a_{i,1} = 0$ in period 1 makes zero profits.

No aggregate uncertainty. Suppose first that there is no aggregate uncertainty, that is, $z_1 = z_2 = 1$. Then the condition pinning down the cutoff productivity of the marginal entrepreneur who

[OC.1] This setup, where there are no opportunities after a production spell, resembles our assumption in the full model that entrepreneurs 'die' – in the sense of having zero value – upon separation.

is indifferent between either entering in the first period or waiting is

$$-\kappa + \bar{h}(1+\widehat{a}_{1,\Delta=0}) = \int_{-\bar{a}}^{\bar{a}} \max\{-\kappa + \bar{h}(1+a), 0\}\, dF(a), \tag{OC.1}$$

where $\widehat{a}_{1,\Delta=0}$ denotes the cutoff productivity level in the absence of aggregate uncertainty. Cancelling terms and rewriting the integral, this equation can be written as[OC.2]

$$\begin{aligned} \widehat{a}_{1,\Delta=0} &= \int_{0}^{\bar{a}} af(a)da, \\ &= \underbrace{E[a|a \geq 0]}_{\equiv a_2^*}\underbrace{\text{prob}(a \geq 0)}_{p_2} > 0, \end{aligned} \tag{OC.2}$$

where a_2^* is the expected value of a_2 conditional on being above the period-2 cutoff level, and p_2 denotes the probability of such a draw.

The fact that $\widehat{a}_{1,\Delta=0} > 0$ means that there is a standard option value of waiting due to idiosyncratic uncertainty in this model. Other things equal, the presence of idiosyncratic uncertainty encourages unmatched entrepreneurs to wait, because doing so preserves the optionality of obtaining a better draw in the future and entering; they can always not enter given a poor draw (as indicated by the max-operator), which eliminates downside risk. This mechanism affects the steady state of the main model. [OC.3] Next we will prove that this option value of waiting is amplified if the anticipated variance of z_2, Δ, is positive.

Aggregate uncertainty. The intuitive logic behind the option-value effect due to aggregate uncertainty is that in a time of high overall productivity, the level of idiosyncratic productivity needed to cover vacancy posting costs is lower, so that the probability of having such a draw is higher; at the same time, the expected value of producing conditional upon entry is higher. The opposite is true in times of low productivity, but the decrease in expected profits is lower because

[OC.2] To derive the following expressions, we use two facts: first, an entrepreneur with a draw $a_i < 0$ prefers to not enter in period 2 and, instead, makes zero profits (reflecting the max operator); second, given the fixed hiring rate we can use that $\kappa/\bar{h} = z_1 = z_2 = 1$ to simplify the expression.

[OC.3] One objective of the recalibration procedure described in section 4.2 is, then, to ensure that this option-value effect does not lower vacancy posting (and, hence, raise unemployment) in steady state – "steady state" in the sense of there being no aggregate uncertainty – below the calibration target.

entrepreneurs with low draws avoid entering in the first place and, instead, make zero profits.

To capture this intuition, we can use the same type of expression for the cutoff when there is no aggregate risk. Note first that the cutoff in period 2 satisfies $-\kappa+\bar{h}(z_2+\widehat{a}_2)=0$, so that $\widehat{a}_2=-\Delta$ in good times, and $\widehat{a}_2=+\Delta$ in bad times. Then denoting by $\widehat{a}_{1,\Delta>0}$ the cutoff productivity level in the presence of aggregate uncertainty, the cutoff equation becomes[OC.4]

$$\widehat{a}_{1,\Delta>0}=\frac{1}{2}\int_{-\Delta}^{\bar{a}}(a+\Delta)f(a)da+\frac{1}{2}\int_{+\Delta}^{\bar{a}}(a-\Delta)f(a)da.$$

The different lower limits of integration indicate that when $z_2=1+\Delta$, entrepreneurs with draws above $-\Delta$ will make positive profits, whereas when $z_2=1-\Delta$, only those with draws above $+\Delta$ will do so. Subtracting equation (OC.2) from equation (OC.3), we obtain

$$\widehat{a}_{1,\Delta>0}-\widehat{a}_{1,\Delta=0}=\frac{1}{2}\left(\int_{-\Delta}^{\bar{a}}(a+\Delta)f(a)da-\int_{0}^{\bar{a}}af(a)da\right)+\frac{1}{2}\left(\int_{+\Delta}^{\bar{a}}(a-\Delta)f(a)da-\int_{0}^{\bar{a}}af(a)da\right).$$

The first term in brackets is positive and the second is negative. However, the first term is larger in absolute value. Figure OC.1a illustrates this point. The dark grey area corresponds to the gain in profits when $z_2=1+\Delta$ rather than $z_2=1$; this is the first term. The lighter grey area describes the loss in profits when $z_2=1-\Delta$ instead; the second term. Clearly, the former area is greater than the latter. The reason is that the entrepreneur has a greater chance of making a draw that yields strictly positive profits in good times. The top-right panel OC.1b illustrates how, when deciding whether to enter or wait in the first period, the greater expected profits in the presence of aggregate risk pushes up the option value of waiting. Consequently, the cutoff level above which entrepreneurs are willing to enter in the first period is higher when $\Delta>0$ than when $\Delta=0$.

Analytical characterization given uniform distribution. To conclude this section, we analytically characterize the cutoff levels $\widehat{a}_{1,\Delta=0}$ and $\widehat{a}_{1,\Delta>0}$ under a particular functional form assumption

[OC.4] Deriving this expressions involves the same steps noted in Footnote OC.2, adjusted for the fact that the period-2 cutoff now is a function of the stochastic aggregate state, z_2.

Figure OC.1: Two-period model

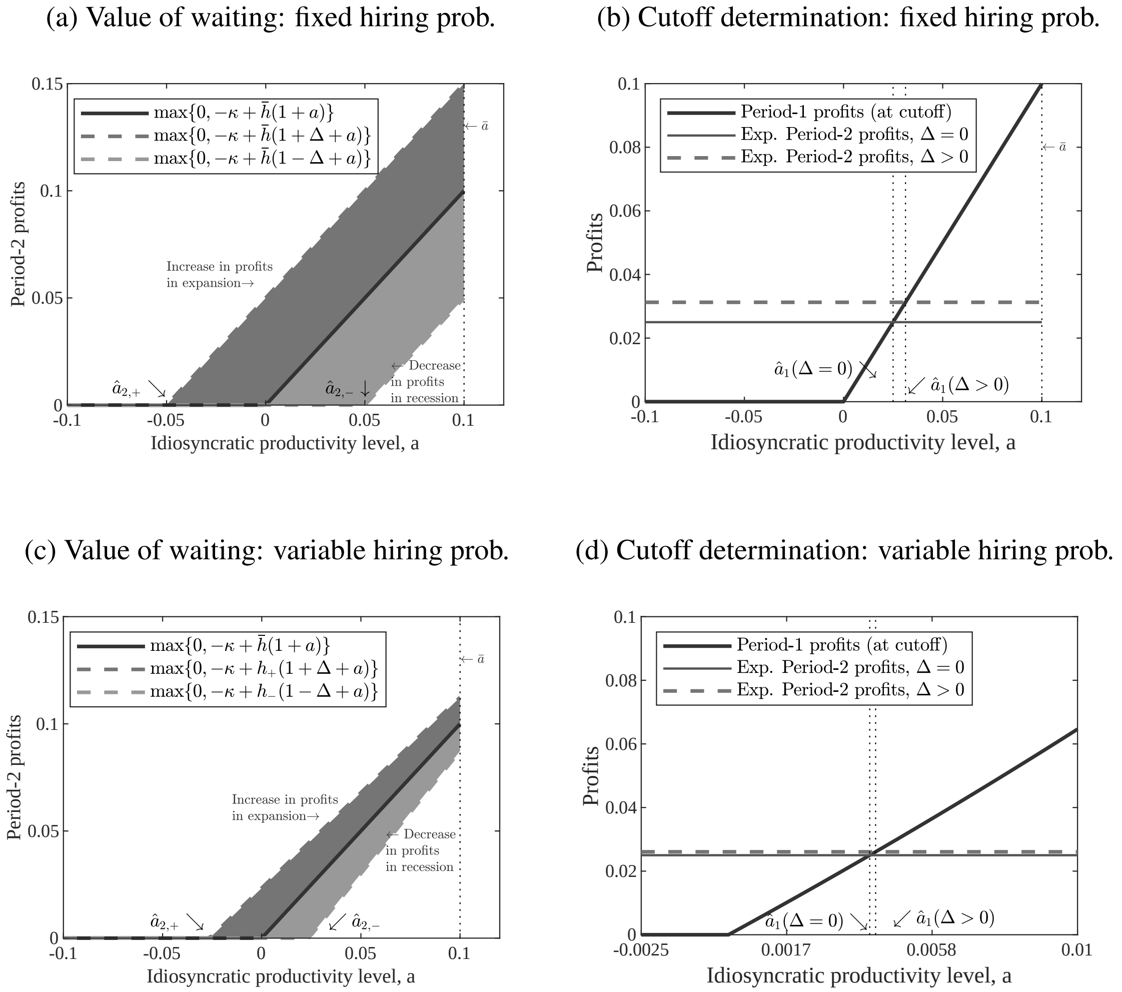

Notes: The figure shows how idiosyncratic and aggregate uncertainty both give rise to an option value of waiting in the two-period model. In the upper row, the hiring rate is fixed at $\bar{h}$ (corresponding to $\alpha = 0$), whereas the lower row allows that rate to vary according to a standard matching function ($\alpha = 0.5$). Throughout, idiosyncratic productivity is distributed uniformly over $[-0.1, +0.1]$. To ease visual comparison, the panels with fixed hiring rate assume that $\Delta = 0.05$, whereas in the case of a variable hiring rate, $\Delta = 0.15$. Finally, notice that in Panel OC.1d, period-1 profits are computed as the left-hand side of equation (OC.6), so that the slope incorporates endogenous changes in the hiring rate. To facilitate a straightforward comparison of alternative vacancy posting cost interpretations in section OF.1, the steady-state hiring rate is set equal to one. As a consequence, $h_- > 1$, which is, strictly speaking, inconsistent with h describing a probability, but intuitively just means that one entrepreneur could employ more than one worker.

about the distribution F, namely, that a_i is uniformly distributed over $[-\bar{a}, +\bar{a}]$. Then

$$p_t = \text{prob}(a \geq \widehat{a}_t) = \frac{\bar{a} - \widehat{a}_t}{2\bar{a}}, \quad \text{(OC.3)}$$

$$a_t^* = E[a | a \geq \widehat{a}_t] = \frac{\bar{a} + \widehat{a}_t}{2}. \quad \text{(OC.4)}$$

To make the connection between the two-period model and the full model very explicit, write the cutoff condition as follows:

$$-\kappa + \bar{h}\underbrace{(1 + \widehat{a}_1)}_{J_1(\widehat{a}_1)} = 0 + E_1\left[p_2(-\kappa + \bar{h}\underbrace{(z_2 + a_2^*)}_{J_2(a_2^*)}) + (1 - p_2)0\right].$$

The expectations operator E_1 conditions on the information available in period $t = 1$. The entire right-hand side describes the value of waiting, J^U. It proves instructive to re-write it as follows:

$$\begin{aligned} J^U &= -\kappa E_1[p_2] + \bar{h} E_1[p_2 J_2(a_2^*)] \\ &= -\kappa E_1[p_2] + \bar{h}\Big(E_1[p_2]E_1[J_2(a_2^*)] + \text{Cov}_1[p_2, J_2(a_2^*)]\Big). \end{aligned}$$

To characterize the expectation terms on the right-hand side, use the fact that $\widehat{a}_2 = -\Delta$ in good times, and $\widehat{a}_2 = +\Delta$ in bad times. Thus,

$$\begin{aligned} E_1[p_2] &= \frac{1}{2}\left(\frac{\bar{a} - \Delta}{2\bar{a}}\right) + \frac{1}{2}\left(\frac{\bar{a} + \Delta}{2\bar{a}}\right) = \frac{1}{2}, \\ E_1[J_2(a_2^*)] &= \frac{1}{2}\left(1 + \Delta + \frac{\bar{a} - \Delta}{2}\right) + \frac{1}{2}\left(1 - \Delta + \frac{\bar{a} + \Delta}{2}\right) = 1 + \frac{\bar{a}}{2}, \\ \text{Cov}_1[p_2, J_2(a_2^*)] &= E_1\{(p_2 - E_1[p_2])\,(J(a_2^*) - E_1[J(a_2^*)])\} = \frac{\Delta^2}{4\bar{a}} \geq 0. \end{aligned}$$

Substituting these expressions into equation (OC.4) and cancelling terms, we find that the cutoff-level of productivity in period $t = 1$ above which entrepreneurs enter is

$$\widehat{a}_1 = \frac{\bar{a}}{4} + \frac{\Delta^2}{4\bar{a}}. \quad \text{(OC.5)}$$

The cutoff level is positive even in the absence of aggregate uncertainty, $\widehat{a}_{1,\Delta=0} = \frac{\bar{a}}{4}$, but the option value of waiting is greater when Δ is positive due to the covariance term, with $\frac{\partial \widehat{a}_1}{\partial \Delta} = \frac{\Delta}{2\bar{a}}$. Intuitively, what this covariance term captures is the following. When the aggregate state of the economy is high, even entrepreneurs with relatively low idiosyncratic productivity can recover the costs of posting a vacancy through production, so that the probability of having a draw that is sufficiently good to incentivize entry is greater. At the same time, the expected value of producing conditional upon entry is higher. The opposite is true in times of low productivity – when only few entrepreneurs draw sufficiently high idiosyncratic productivity values – but the decrease in expected profits is lower, because entrepreneurs with low draws avoid entering in the first place and, instead, make zero profits. An increase in aggregate volatility makes the covariance term correspondingly larger. To illustrate, Figures OC.1a and OC.1b are drawn for $\bar{a} = 0.1$ and $\Delta = 0.05$, such that $\widehat{a}_{1,\Delta=0} = 0.025$ and $\widehat{a}_{1,\Delta=0} = 0.03125$.

It is interesting to note that the effect on the cutoff from a change in the period-2 variance of aggregate productivity is larger for smaller values of $\bar{a}$ and becomes ill-defined as $\bar{a}$ approaches zero. The intuitive explanation is that when idiosyncratic dispersion is small, firms are clustered around the cutoff.[OC.5] In particular, when $\bar{a}$ is close to zero, tiny changes in Δ would imply hitting the bounds of the distribution. This is not taken into account by the derivative. For this reason, it is instructive to consider the case without idiosyncratic risk.

Aggregate uncertainty but no idiosyncratic uncertainty. Suppose that $a_{i,1} = a_{i,2} = 0$ for all firms, and that there is a unit mass of firms. Then in the absence of aggregate uncertainty, all entrepreneurs are indifferent between creating a firm and not doing so. Now suppose that a fraction $\frac{1}{2}$ of all firms does create a firm in the first period. Even a tiny introduction of aggregate uncertainty regarding z_2 will induce a discontinuous jump from $\frac{1}{2}$ to 1. This is effectively what the derivative $\frac{\partial \widehat{a}_1}{\partial \Delta} = \frac{\Delta}{2\bar{a}}$ tells us.

[OC.5] A second objective of our recalibration procedure is to counteract the fact that for greater degrees of dispersion changes in the cutoff level induce smaller changes in the probability of entry and, hence, the number of jobs created. To this end, we adjust the sensitivity of entrepreneurial profits to changes in aggregate productivity upward when idiosyncratic dispersion is greater. (This is achieved by making the entrepreneurial profit share in output smaller, an aspect we abstract from in the two-period model.)

OC.2 Variable hiring rate

Instead of the hiring rate being invariant to economic conditions, suppose now that there is congestion in the labor market.

Environment. Matches are determined according to a Cobb-Douglas function, as in the full model. As the separation rate is one and 'dead' entrepreneurs are replaced by new ones, the number of vacancies posted is simply $v_t = p_t \Upsilon$, so that the hiring rate is $h_t = \psi(\Upsilon p_t)^{-\alpha}$, where Υ is the mass of entrepreneurs, ψ is the matching efficiency, and $(1-\alpha)$ the elasticity of matches with respect to vacancies. As the goal here is analytical clarity rather than targeting a particular unemployment rate, we set Υ equal to one. To ensure that in the absence of uncertainty we have that $h = \bar{h}$ and $p = 1/2 \Leftrightarrow \widehat{a} = 0$, set $\psi = \bar{h}\left(\frac{1}{2}\right)^{\alpha}$ and $\kappa/\bar{h} = 1$.[OC.6]

Graphical analysis. Three key implications of the amended description of the model environment can be understood immediately from the graphical exposition in Figure OC.1. In particular, the bottom row of panels parallels the top row in terms of their construction, but now the hiring rate is endogenous.

Firstly, as before, there exist option-value effects due to idiosyncratic uncertainty ($\widehat{a}_{1,\Delta=0} > 0$) and due to aggregate uncertainty ($\widehat{a}_{1,\Delta>0} > \widehat{a}_{1,\Delta=0}$). In particular, the figure underscores that greater anticipated uncertainty over period-2 aggregate productivity renders expected period-2 profits higher, strengthening any motive for entrepreneurs to wait due to idiosyncratic productivity dispersion. As such, endogenous matching does not alter the central, qualitative message of section OC.1. There are, however, two forces that dampen the magnitude of such option-value effects.

For one, the sensitivity of the value of waiting to aggregate uncertainty is lessened. As Panel OC.1c shows, both the *increase* in profits in an expansion and the *decrease* in profits in a recession are muted relative to the case of a fixed hiring rate. Intuitively, in an expansion, congestion on the entrepreneurs' side of the matching market means that the hiring rate is lower than in steady state or when the hiring rate is fixed (as reflected in the flatter slope of the dashed profit line). Hence, the increase in profits is muted, as more vacancies posted remain unfilled. The opposite is true in a

[OC.6] In a model with positive wages, we would set $\kappa/\bar{h} = Q$, where Q denotes steady-state profits.

recession, when the probability of a successful match conditional on posting a vacancy is greater. Because the lower hiring rate in an expansion applies to a wider range of idiosyncratic productivity values, the net effect is to limit the rise in expected profits due to aggregate uncertainty. Therefore, endogenous matching dampens the rise in the option value of waiting when uncertainty over future aggregate productivity is elevated.

More importantly still, for any given change in the value of waiting, the increase in the period-1 cutoff and, thus, the impact of period-2 uncertainty on period-1 economic activity, is much smaller than when the hiring rate is fixed. For any given increase in expected period-2 profits due to either idiosyncratic or aggregate uncertainty, the equilibrium change in the period-1 cutoff and, hence, vacancy posting, is much smaller (Panel OC.1d). The reason is that any increase in the cutoff lowers the share of entrepreneurs entering and, hence, the amount of congestion. The endogenous rise in the hiring rate makes entering more attractive, leading to a partial offsetting of the increase in the cutoff that pushed up the hiring rate in the first place. The converse holds for a fall in the cutoff. This general equilibrium dampening force is suppressed when the hiring rate is constant.

While the graphical analysis communicates the central implications of allowing for matching frictions, for the interested reader we next consider again in detail the different configurations of idiosyncratic and aggregate uncertainty. We start with the case where idiosyncratic dispersion is zero but aggregate uncertainty is positive, as in the benchmark search-and-matching model.

Aggregate uncertainty but no idiosyncratic uncertainty. Suppose first that $a_{i,1} = a_{i,2} = 0$ for all firms. As in the case of a fixed hiring rate, in the absence of aggregate uncertainty all entrepreneurs are indifferent between creating a firm and not doing so. Suppose that initially a fraction $\frac{1}{2}$ does. In a marked difference from the previous case, now the introduction of a tiny amount of aggregate uncertainty does *not* lead to a discontinuous jump from $\frac{1}{2}$ to 1. To the contrary, when Δ is positive (but small), the entry probability p_2 and, hence, the hiring probability h_2 adjusts such that expected profits are equal to zero both when $z_2 = 1+\Delta$ and when $z_2 = 1-\Delta$.[OC.7] As such,

[OC.7] As emphasized in section 4, this argument presumes that the constraint on the available number of entrepreneurs is sufficiently slack – respectively, the shocks sufficiently small –so that movements in the hiring rate can render expected profits equal to zero.

there is no option value of waiting and p_1 is still equal to $1/2$. Thus, the endogenous adjustment of the hiring probability plays a key role in ensuring that the free-entry condition holds in every state of the world.

Idiosyncratic uncertainty but no aggregate uncertainty. Next, consider the option value of waiting due to firm-level dispersion but absent aggregate uncertainty. With a variable hiring rate, the cutoff equation becomes

$$-\kappa + \underbrace{\psi p_1^{-\alpha}}_{h_1}(1+\widehat{a}_1) = p_2 \left(-\kappa + \underbrace{\psi p_2^{-\alpha}}_{h_2}(1+\widehat{a}_2) \right) > 0,$$

and it implies that the value of waiting in period 1 still is positive. For notice that our calibration ensures that when $\Delta = 0$, the period-2 cutoff is always equal zero, with half of all entrepreneurs entering, so that $h_2 = \bar{h}$. But the conditional expected value is positive provided $\bar{a} > 0$, that is, $a_2^* > \widehat{a}_2 = 0$.[OC.8] Thus, an increase in idiosyncratic dispersion, that is in $\bar{a}$, unambiguously raises the right-hand side by raising a_2^*. For the left-hand side to increase also, it must be that $\widehat{a}_1$ increases, as p_1 is strictly decreasing in $\bar{a}$ when $\widehat{a}_1 > 0$.

In contrast to the case with fixed matching probabilities, however, $\widehat{a}_1$ increases more gradually as the degree of idiosyncratic dispersion increases. The reason is that a variable hiring rate introduces a natural dampening effect: when the cutoff is higher and more entrepreneurs choose to wait, the hiring probability increases, which makes entering more attractive again. Specifically, whereas in the case of a fixed hiring rate, $\frac{\partial J_1(\widehat{a}_1)}{\partial \widehat{a}_1} = \bar{h}$, we now have

$$\frac{\partial J_1(\widehat{a}_1)}{\partial \widehat{a}_1} = h_1 + (1+\widehat{a}_1)\frac{\partial h_1}{\partial \widehat{a}_1}, \qquad \text{where}$$
$$\frac{\partial h_1}{\partial \widehat{a}_1} = \frac{\alpha}{2\bar{a}} \left(\frac{\bar{a} - \widehat{a}_1}{2\bar{a}} \right)^{-(\alpha+1)}.$$

As such, a small increase in $\widehat{a}_1$ following a rise in idiosyncratic uncertainty is sufficient for the

[OC.8] The period-2 cutoff level, $\widehat{a}_2$, satisfies $-\kappa + \psi p_2^{-\alpha}(1+\widehat{a}_2) = 0$ and given the uniform distribution we have that $p_2 = \frac{\bar{a} - \widehat{a}_2}{2\bar{a}}$.

left-hand side to be equal to the – now greater – right-hand side.[OC.9]

Idiosyncratic and aggregate uncertainty. Finally, when both $\bar{a} > 0$ and $\Delta > 0$, the cutoff equation with endogenous matching is

$$-\kappa + h_1(1 + \widehat{a}_1) = E_1\left[p_2(-\kappa + h_2(z_2 + a_2^*))\right]. \tag{OC.6}$$

As in the case without aggregate uncertainty that we just considered, for any given change in $\widehat{a}_1$, the fact that h_1 is an increasing function of $\widehat{a}_1$ introduces a first dampening force relative to the model with fixed hiring rate. What about the magnitude of the value of waiting?

Consider the right-hand side of equation (OC.6), that is, J^U. In period 2, we have the following two expressions that implicitly define the cutoff levels (recall that p_2 is a function of the cutoff itself).

$$-\kappa + h_{2,+}(1 + \Delta + \widehat{a}_{2,+}) = 0 \Leftrightarrow \widehat{a}_{2,+} = \frac{\kappa}{\psi}\left(\frac{\bar{a} - \widehat{a}_{2,+}}{2\bar{a}}\right)^{\alpha} - 1 - \Delta,$$
$$-\kappa + h_{2,-}(1 - \Delta + \widehat{a}_{2,+}) = 0 \Leftrightarrow \widehat{a}_{2,-} = \frac{\kappa}{\psi}\left(\frac{\bar{a} - \widehat{a}_{2,-}}{2\bar{a}}\right)^{\alpha} - 1 + \Delta.$$

Thus, relative to the case with fixed hiring rate, the cutoff in an expansion, $\widehat{a}_{2,+}$, is higher, while the cutoff in a recession, $\widehat{a}_{2,-}$, is lower. The reason is that the effective hiring costs to be covered by revenue over the duration of a match are greater (smaller) in an expansion (recession), when the hiring probability is lower (higher).

[OC.9] This relationship is non-linear, insofar as a change in the cutoff of a given size leads to a more modest change in the entry probability and, hence, hiring rate for a more dispersed distribution.

We can rewrite the value of waiting as a a function of these cutoffs.

$$
\begin{aligned}
J^U &= \frac{1}{2}E\left[p_{2,+}\left(-\kappa+\psi(p_{2,+})^{-\alpha}(1+\Delta+a)\right)|a\geq\widehat{a}_{2,+}\right] \\
&+\frac{1}{2}E\left[p_{2,-}\left(-\kappa+\psi(p_{2,-})^{-\alpha}(1-\Delta+a)\right)|a\geq\widehat{a}_{2,-}\right], \\
&=\frac{1}{2}\left[-\kappa\left(\frac{\bar{a}-\widehat{a}_{2,+}}{2\bar{a}}\right)+\left(\psi\left(\frac{\bar{a}-\widehat{a}_{2,+}}{2\bar{a}}\right)^{1-\alpha}\left(1+\Delta+\frac{\bar{a}+\widehat{a}_{2,+}}{2}\right)\right)\right] \\
&+\frac{1}{2}\left[-\kappa\left(\frac{\bar{a}-\widehat{a}_{2,-}}{2\bar{a}}\right)+\left(\psi\left(\frac{\bar{a}-\widehat{a}_{2,-}}{2\bar{a}}\right)^{1-\alpha}\left(1-\Delta+\frac{\bar{a}+\widehat{a}_{2,-}}{2}\right)\right)\right].
\end{aligned}
$$

While no explicit solution is possible, the expressions nevertheless reveal very intuitive implications of making the hiring rate endogenous.[OC.10] Recall that in the case of a fixed hiring rate (nested here for $\alpha = 0$), the option value of waiting was increasing in the anticipated variance of period-2 productivity, Δ, because the entry probability and the conditional expected value of a match co-varied positively, that is, $\mathrm{Cov}_1[p_2, J_2(a_2^*)] > 0$. This same effect is still operative here, but its quantitative magnitude is dampened, because even though in an expansion the probability of entry is higher, congestion externalities mean that the hiring probability is lower, which limits the conditional expected value of entering. The converse holds true in a recession. That is, $\mathrm{Cov}_1[h_2, J_2(a_2^*)] < 0$. The expressions also underscore that since the hiring rate only ever moves due to changes in the entry probability, this mechanism can only ever dampen but not completely eliminate the option value of waiting due to aggregate uncertainty, provided that $\bar{a} > 0$.

In summary, the two-period model illustrates the existence of option-value considerations in a model with a finite mass of entrepreneurs and firm-specific productivity. Allowing for congestion in the matching market is crucial because it ensures that introducing even the tiniest amount of aggregate uncertainty does not lead to a discontinuous rise in the value of waiting. At the other end of the extreme, in the absence of heterogeneity in firm-specific productivity (and presuming a sufficiently large number of potential entrepreneurs), such endogenous variations in the hiring probability entirely eliminate any option-value effects. The model with a variable hiring rate, as in

[OC.10] An additional mechanism is that the rise in the hiring rate in a recession is greater than its decline in a boom – the matching function being concave – so that aggregate uncertainty raises the expected hiring rate.

the standard search-and-matching environment, and a finite mass of entrepreneurs that vary in terms of their productivity draws represents a potentially attractive middle way.

Appendix OD Variations on the heterogeneous-firm model

The heterogenous-firm version of the search-and-matching (SaM) model developed and analyzed in the main text supposes that entrepreneurs "die" upon separation; and that the mass of of potential entrepreneurs is both finite and constant. This section shows how the model may be modified or extended to allow for a relaxation of these two assumptions, respectively, and with what implications. These examinations shed further light on, and underscore the importance of, the mutual exclusivity property that needs to obtain in order for an option-value channel of waiting to exist through which uncertainty shocks may affect macroeconomic activity.

OD.1 Infinitely-lived entrepreneurs

In the baseline model, entrepreneurs have finite lives; specifically, following a separation shock, the entrepreneur "dies" and gets replaced. Accordingly, the match value given idiosyncratic productivity a is

$$J_t(a) = (1-\omega)(\bar{x}(z_t + a) - \chi) + \beta(1-\delta)E_t[J_{t+1}(a)]. \qquad \text{(OD.1)}$$

This specification has the benefit of rendering the transmission of uncertainty shocks very transparent, since holding constant a and the sequence of aggregate productivity levels, $\{z_t\}_{t=0}^{\infty}$, the match value, $J_t(a)$, is invariant to an increase in perceived volatility. Consequently, any macroeconomic effects triggered by such an increases can be traced back solely to variations in the (expected) value of waiting, $E_t[J_{t+1}^U]$, consistent with the standard option-value mechanism for postponing investment. Lastly, it ought to be noted that equation (OD.1) closely mimics the firm value in the baseline model with homogenous firms.

An alternative model supposes, instead, that entrepreneurs are infinitely lived. Specifically, suppose that following separation the entrepreneur "survives" and with probability $(1-\gamma)$ she can immediately draw a new idiosyncratic value, a, but that she remains idle for one period with the complementary probability (and in the next period this process repeats itself). Thus, $1-\gamma$ is the

Poisson arrival rate of new "business projects", and $1/(1-\gamma)$ is the expected duration of "idleness" following separation.

In this setup, the value of a matched entrepreneur given idiosyncratic productivity a is as follows:[OD.1]

$$J_t(a) = (1-\omega)(\bar{x}(z_t + a) - \chi) + \beta(1-\delta)E_t[J_{t+1}(a)] + \beta\delta E_t[\hat{J}^U_{t+1}], \tag{OD.2}$$

where

$$\hat{J}^U_t = (1-\gamma)J^U_t + \beta\gamma E_t[\hat{J}^U_{t+1}]. \tag{OD.3}$$

denotes the value of a newly separated, idle entrepreneur. Here, J^U_t continues to be the value of an unmatched entrepreneur prior to the revelation of her a in period t.

Moreover, the equation giving the number of vacancies posted needs to be adjusted to account for the fact that some of the unmatched entrepreneurs are unavailable because they have not yet had the chance to draw a new productivity value. Equation (21) is, thus, replaced by

$$v_t = p_t\left(\Upsilon - (1-\delta)n_{t-1} - \delta\gamma s_{t-1}\right), \tag{OD.4}$$

$$s_t = n_t + \gamma s_{t-1}. \tag{OD.5}$$

All remaining equations are unchanged. In particular, the cutoff-level of idiosyncratic productivity continues to be pinned down by

$$h_t(J_t(\hat{a}_t) - \beta E_t[J^U_{t+1}]) - \kappa = 0. \tag{OD.6}$$

To identify how this model variation affects the transmission of uncertainty shocks relative to

[OD.1] See section OE.2 of the online appendix for a derivation. There we show that this model may be interpreted as describing the vacancy-posting decisions of a representative firm with multiple business projects, some of which are matched with a work and, hence, productive, while others are idle.

the baseline, both qualitatively and quantitatively, it is instructive to initially consider the special case where γ is set to zero. This means that upon separation any entrepreneur simply switches to the state of being an unmatched entrepreneur who, in each subsequent period in which she remains unmatched, can draw a new a. In that case, relative to the baseline model with finitely-lived entrepreneurs the only equation that is changed is that for the match value. Namely, for $\gamma = 0$, equation (OD.2) simplifies to

$$J_t(a) = (1-\omega)(\bar{x}(z_t + a) - \chi) + \beta E_t \left[(1-\delta) J_{t+1}(a) + \delta J^U_{t+1} \right]. \tag{OD.7}$$

According to this specification, an entrepreneur that commits and creates a job in the current period still benefits from increased uncertainty, although only at some point in the future, namely after the created relationship ends. The option-value of waiting effect considered in the main text is still present in this version, however, its effect is dampened since increased uncertainty now affects both the option of waiting and the commitment to investment immediately.

Figure OD.1 provides a quantitative examination, whereby the solid lines indicate pure uncertainty effects in the baseline model with finitely lived entrepreneurs and the dashed version corresponds to the alternative version with infinitely-lived entrepreneurs and γ set to zero.[OD.2] This numerical analysis features certain intricacies that warrant explanation. For one thing, preserving comparability requires that the two models are individually calibrated according to the procedure described in 4.2, that is, taking into account the adjusted form of the match value equation (OD.7). Else, if we imposed the same parameter values on both models, in one case we would miss the calibration targets. Specifically, notice that for a given set of parameter values and σ_a, vacancy-posting is generally, and other things equal, more attractive relative to staying idle in the model with infinitely lived entrepreneurs.[OD.3] As such, for a given value of σ_a, the model without entrepreneur death and $\gamma = 0$ is closer to the homogeneous-firm environment than the baseline. As a consequence,

[OD.2]To aid orientation, observe that the solid lines in Figure OD.1 match the solid lines in Figure 5.

[OD.3]For instance, given the parameter values used for the homogeneous-firm model, the steady-state unemployment rate in the heterogeneous-firm model with infinitely-lived entrepreneurs and $\gamma = 0$, given $\sigma_a = 0.003$, is 6.58 percent (versus a targeted value of 6.4 percent). For comparison, the equivalent value in the model with entrepreneur death is 9.71 percent (see Figure 4).

Figure OD.1: Entrepreneur death (baseline) vs. infinitely-lived entrepreneurs

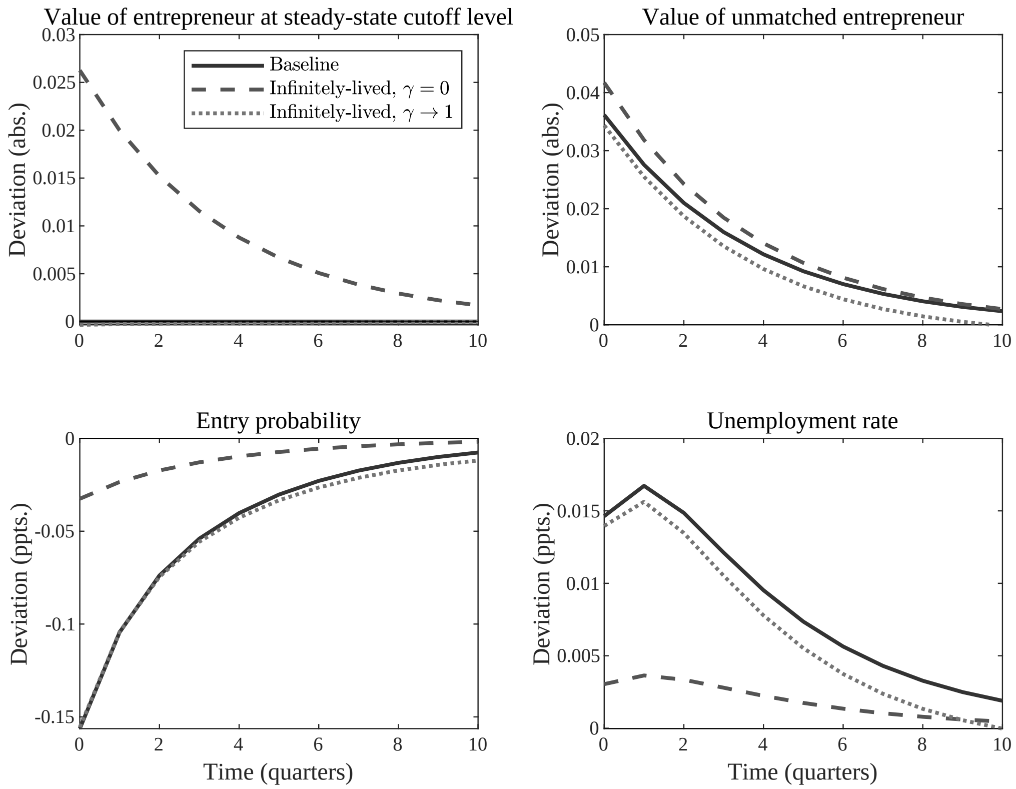

Notes: The figure reports pure uncertainty IRFs to a unit-increase in $\varepsilon_{\sigma,t}$. In the models corresponding to the solid and dashed lines, the model parameters are recalibrated following the procedure described in section 4.2 and using the respectively applicable model equations, i.e., equation (OD.1) for the baseline model with death ($\sigma_a = 0.003$) and equation (OD.7) for the model with infinitely-lived entrepreneurs and $\gamma = 0$ ($\sigma_a = 0.025$). For $\gamma \to 1$ (approximated by setting $\gamma = 0.999$) we impose the same parameters as in the baseline setting.

the upper bound on σ_a in the recalibrated, alternative model is greater than in the model with death, that is, around 0.025 instead of 0.003. To maximize comparability, the IRFs for each model are therefore computed by imposing these respective (approximate) maximum values for σ_a.

Turning to the analysis, the respective pure uncertainty effects on the value functions are intuitive. Whereas in the baseline model, the value of a matched entrepreneur for a given level of idiosyncratic productivity – in this instance, the steady-state cutoff level – does not respond to an increase in uncertainty (solid line), in the alternative specification with $\gamma = 0$ the value of entrepreneurs who are already matched increases as well (dashed line). It is because of this difference that, even though the rise in the value of remaining unmatched is roughly similar in both specifications, the ultimate effects on entrepreneurial entry probabilities and worker unemployment are dampened in the model with infinitely-lived entrepreneurs relative to the baseline. Indeed, the recalibrated model

with infinitely-lived entrepreneurs and $\gamma = 0$ implies an increase in unemployment following an uncertainty shock that is similar in magnitude to the effect obtained in the homogeneous-firm model with Nash bargaining (cf. Figure 1), which is substantially smaller than the baseline.

It is tempting to infer from this two-way comparison that what matters for the magnitude of the option-value effect is whether entrepreneurs are finitely or infinitely lived. That is not quite correct. To see this, consider now the model with infinitely-lived entrepreneurs but setting γ greater than zero. In particular, let $\gamma \to 1$ and adjust the measure of potential entrepreneurs to reflect that a fraction of unmatched entrepreneurs that is increasing in γ are not in a position to post vacancies. With $\Upsilon = \Upsilon^{\text{baseline}} + n\delta\frac{\gamma}{1-\gamma}$, in the limit the steady states of the models with finitely-lived and infinitely-lived entrepreneurs coincide. Moreover, turning to the dynamic effects of uncertainty shocks, the dotted line in Figure OD.1, which is based on the model with infinitely-lived entrepreneurs and $\gamma = 0.999$, almost coincides with the baseline model.[OD.4] This third point of comparison thus underlines that the key question concerns the extent to which uncertainty differentially raises the expected value of waiting as opposed to committing to investing in the present. Even when entrepreneurs are infinitely lived, if committing to a hire now and investing in a different project in the future are mutually exclusive – with certainty in the baseline setup and stochastically in the model presented here – then an increase in aggregate uncertainty makes waiting relatively more attractive, with adverse consequences for hiring activity.

We conclude this section by noting that the model with infinitely-lived entrepreneurs considered in this section still remains unrealistic in that, even while the entrepreneur lives on following a separation, the entrepreneur's firm (or business project) does not. That is, the SaM structure implies that expected firm life coincides with the time we expect the worker to stay with one firm, which is a short span of time (the expected firm life implied by our calibration is two and a half years whereas the uncertainty shock is quite persistent). Clearly, in reality firms are not destroyed and their idiosyncratic productivity independently re-drawn – sooner or later, on average, depending on the

[OD.4] The reason why the two models do not entirely coincide is that in the model with infinitely-lived entrepreneurs, those have to (stochastically) wait for projects to be available again. Hence, past separations affect the likelihood of finding new workers, as is reflected in the state variable s_t.

value of γ, when a worker leaves a new one is hired. An appropriate description of empirical reality suitable for quantitative analysis ought to instead feature idiosyncratic firm productivity that is highly persistent but not permanent. Even under a parameterization like $\gamma = 0$, such a model would imply less dampening relative to the baseline model. However, analyzing such a model requires keeping track of the full distribution of existing firm productivities and is beyond the scope of the current paper. What this section has demonstrated, leveraging the transparent cutoff formulation our framework affords, is that an option-value of waiting effect can propagate uncertainty shocks irrespective of whether entrepreneurs have finite or infinite lives.

OD.2 Endogenous measure of entrepreneurs

The baseline version of the heterogeneous-firm model supposes that the mass of potential entrepreneurs, denoted by Υ, is finite and constant. This stands in sharp contrast with the standard SaM framework, in which there is a potentially infinite mass of entrepreneurs. In this appendix, we discuss an extension of our model which endogenizes the mass of potential entrepreneurs, Υ, who, when idle, receive an idiosyncratic shock, a, enter the matching market when a is sufficiently high, and – if matching is successful – start producing.[OD.5] This model extension further clarifies the conditions under which an option-value of waiting mechanism is operative in SaM environments.

Suppose, then, that at the very beginning of a given period, any one of a potentially infinite number of homogeneous individuals can pay a fixed cost μ to obtain the chance to become such an entrepreneur. As in the standard SaM framework, the outside option for these individuals is equal to zero. Upon paying μ this individual becomes an (initially unmatched) entrepreneur with a time-varying probability $g(\Upsilon_t)$, where g is weakly decreasing in Υ_t. That is, if the number of entrepreneurs is already large, then it is less likely that the new-joiner finds a potential business opportunity, or project, that could be matched to a worker.[OD.6]

[OD.5] We thank an anonymous referee for encouraging us to explicitly discuss an extension of this type.

[OD.6] Observe that g is decreasing in the *stock* of already existing projects rather than the measure of potential entrepreneurs looking to enter. In that respect, the formulation here differs from the use of a standard, matching function involving vacancies and unemployed job seekers. Intuitively, the latter centers on congestion externalities that each side imposes on competing members of their own side. By contrast, here a new-joiner's chances are not only negatively

Accordingly, for $\sigma_a > 0$ the now potentially time-varying total mass of entrepreneurs, Υ_t, satisifies the free-entry condition

$$\mu = g(\Upsilon_t) J_t^U, \tag{OD.8}$$

where J_t^U is the value of an idle entrepreneur (prior to the revelation of this period's idiosyncratic draw) given by equation (20).

It warrants emphasizing that equation (OD.8) is meaningful only because in our modified SaM model with cross-sectional productivity dispersion, and in marked contrast to the standard setup, the value of an unmatched entrepreneur, J_t^U, is no longer zero by construction. It is precisely because being an idle entrepreneur carries an option value that it may be worth paying the fixed cost μ. For that idle entrepreneur has some probability of receiving an idiosyncratic productivity, in the present period or in the future, which is such that the expected profits of posting a vacancy, net of the cost κ of doing so, are strictly positive. In the standard model, on the other hand, there is no value to entering as an entrepreneur.

We take g to have a simple, isoelastic form with constant elasticity $\iota \geq 0$, i.e., $g(\Upsilon) = \Upsilon_t^{-\iota}$. Then the mass of entrepreneurs is simply $\Upsilon_t = \left(\frac{J_t^U}{\mu}\right)^{\frac{1}{\iota}}$, with $\frac{1}{\iota}$ measuring the elasticity of entrepreneurial entry with respect to the (scaled) value of being an unmatched entrepreneur.[OD.7] All remaining parameters are unchanged from the baseline calibration described in the main text.

Figure OD.2 indicates the pure uncertainty effects on selected variables of a volatility shock in this economy, conditional upon different entry elasticities. Consider the solid line first. When ι is very large, we are effectively in the world of our modified SaM model with a fixed mass of entrepreneurs as described in Section 4. An increase in aggregate uncertainty pushes up the value of waiting; the mass of entrepreneurs, Υ_t, remains almost constant because even a tiny increase in Υ_t

affected by other new-joiners but also by established entrepreneurs. The idea is that if established entrepreneurs have already picked the low-hanging fruits, this makes it harder for others to come up with viable projects.

[OD.7] This functional form assumption means that g cannot strictly speaking be interpreted as a probability, as the range of g is not restricted to the interval $[0,1]$. So a better interpretation for g is the number or fraction of projects created when the individual pays μ, which could be a fraction of a project when $g < 1$ and more than one project when $g > 1$. Moreover, this choice of functional form maximizes the comparability to the standard SaM model described in Section 2, where the hiring probability h takes an analogous form.

Figure OD.2: Endogenous measure of potential entrepreneurs

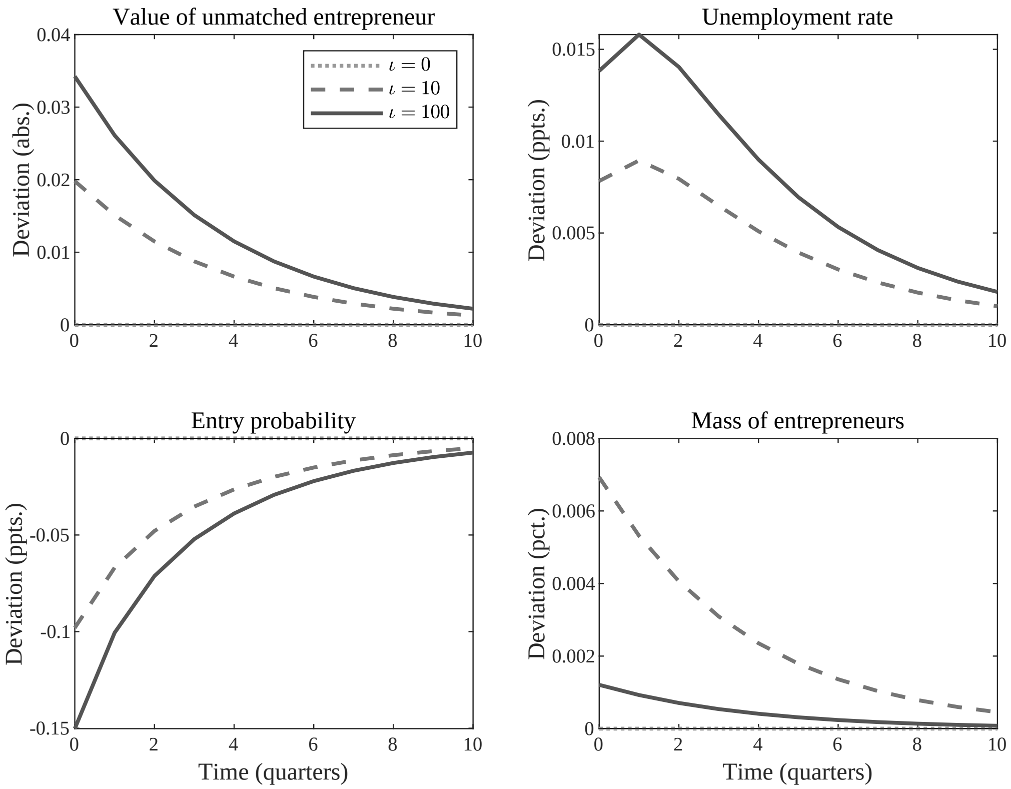

Notes: The figure reports pure uncertainty IRFs to a unit-increase in $\varepsilon_{\sigma,t}$. The model parameters are recalibrated following the procedure described in section 4.2.

sharply raises the cost of entrance; and idle entrepreneurs require a higher value for a to warrant vacancy posting due to the increase in J_t^U. Consequently, unemployment rises.

At the other extreme, when $\iota = 0$, the pure uncertainty effects on the unemployment rate are uniformly zero. They thus coincide with the results documented for the standard SaM model in Section 3 given a linear wage rule – despite the fact that there is dispersion in idiosyncratic productivity. When $\iota = 0$, g_t is constant which in turn implies that J_t^U is constant. And although J_t^U is positive rather than zero as in the standard SaM model, it being constant implies that there cannot be an increase in the option value of waiting when uncertainty increases. As shown in the figure, there is no change in the unemployment rate. Thus, just as a perfectly elastic entry-margin at the level of vacancy-posting eliminated any pure uncertainty effects in a standard SaM model, so does perfectly elastic entry at the level of entrepreneurs in this modified model with heterogeneous firms when $\iota = 0$. Lastly, the dashed line in Figure OD.2 captures the intermediate case where an increase in the number of entrepreneurs following a rise in uncertainty dampens, without eliminating, the

adverse consequences of uncertainty for vacancy posting and, ultimately, for unemployment.

Appendix OE Representative-firm formulations

The main analysis adopts the common approach used in the search-and-matching (SaM) literature in which there are n_t basic production units and each consists of an entrepreneur who has successfully hired a single worker by investing in a vacancy. We refer to this as the "baseline" formulation in the following. It is well known that this baseline formulation is equivalent to a representative-firm formulation with n_t workers for the standard SaM model (see, e.g., Petrosky-Nadeau *et al.* (2018)). This appendix shows that there also exists a representative-firm formulation with multiple workers for our proposed alternative SaM model with heterogeneity in idiosyncratic productivity. Thus, the framework is silent with regards to if movements in the unemployment rate are driven by existing firms changing their stock of employees, or if there is variations in the creation of new firms. Both interpretations are equally consistent within the framework.

OE.1 Standard model

We first briefly remind the reader of the equivalence between the two environments for the standard SaM model. Recursively written, the Bellman equation for a firm with n_{t-1} employees is

$$v(n_{t-1}, z_t) = \max_{n_t, v_t} \{\bar{x} z_t n_t - w_t n_t - \kappa v_t + \beta(1-\delta) E_t V(n_t, z_{t+1})\}. \tag{OE.1}$$

and its perceived law of motion reads

$$n_t = (1-\delta) n_{t-1} + h(\theta_t) v_t. \tag{OE.2}$$

The firm takes as given the wage w_t and the hiring probability $h(\theta_t)$.

Denoting by J_t the Lagrange multiplier on equation (OE.2), the first-order condition for vacancies

then reads

$$0 = -\kappa + h(\theta_t)J_t, \tag{OE.3}$$

where from differentiating equation (OE.1) with respect to n_t, that multiplier satisfies the envelope condition

$$J_t = \bar{x}z_t - w_t + \beta(1-\delta)E_t J_{t+1}. \tag{OE.4}$$

Clearly, the last two equations coincide with the free-entry condition and the value of a match, respectively, as they were written in the main text (cf. equations (3) and (1)).

OE.2 Heterogeneous-firm model

Next, suppose there is a representative firm with the capacity to manage a finite, and constant, mass of projects, Υ.[OE.1] In analogy to the setups described in sections 4.1 and OD.1, projects can be either productive, when matched with a worker, or idle. To be precise, we show the representative-firm representation exists for the model with infinitely-lived entrepreneurs discussed in appendix OD.1. The reason is that the assumption analogous to entrepreneur "death" for projects would seem less appealing; it would imply that the firm shrinks to zero over time.[OE.2] Thus, suppose that projects that were previously matched with a worker become again unmatched projects following an exogenous separation shock, which occurs at a rate δ. The probability that such an idle project becomes potentially productive again, so that the firm draws a new idiosyncratic productivity value for it and can potentially match it with a worker, is $1-\gamma$.

[OE.1] That is, one may think of there being a unit continuum of identical firms that each have the same, finite and constant mass of projects Υ, of which a fraction n_t is matched with a worker in a given period, and which take the wage and hiring probability as given.

[OE.2] The proof of an equivalence proposition for the model with "death" involves somewhat lengthy and tedious algebra; it is available on request.

Given this setup, the value function for a firm with n_{t-1} employees then is

$$v(n_{t-1},A_{t-1},z_t,s_{t-1}) = \max_{\hat{a}_t,A_t,n_t} \{(1-\omega)(\bar{x}(n_t z_t + A_t) - n_t\chi) - \kappa[\Gamma_t(1-F(\hat{a}_t))] + \beta E_t[v(n_t,A_t,z_{t+1},s_t))]\},$$

subject to

$$n_t = h_t\Gamma_t(1-F(\hat{a}_t)) + (1-\delta)n_{t-1},$$
$$A_t = (1-\delta)A_{t-1} + h_t\Gamma_t(1-F(\hat{a}_t))a_t^*,$$

with

$$a_t^* = E[a|a \geq \hat{a}] = \frac{1}{1-F(\hat{a}_t)}\int_{\hat{a}_t}^{\bar{a}} adF(a),$$

and

$$\Gamma_t = \Upsilon - (1-\delta)n_{t-1} - \delta\gamma s_{t-1},$$

and

$$s_t = n_t + \gamma s_{t-1},$$

Notice that, as in the main text, we imposed the linear wage rule. Further, A_t is equal to the total contribution of the idiosyncratic components to firm output. In other words, it is equal to n_t times the average of the idiosyncratic values of all the projects the firm operates.

The first order condition for the cutoff level of project-specific productivity, $\hat{a}_t$, is

$$[(1-\omega)(\bar{x}z_t-\chi)+\beta E_t[v_n(n_t,A_t,z_{t+1},s_t)]+\beta E_t[v_s(n_t,A_t,z_{t+1},s_t)]]\frac{\partial n_t}{\partial \hat{a}_t}$$
$$+[(1-\omega)\bar{x}+\beta E_t[v_A(n_t,A_t,z_{t+1},s_t))]]\frac{\partial A_t}{\partial \hat{a}_t}+\kappa\Gamma_t f(\hat{a}_t)=0,$$

with

$$\frac{\partial n_t}{\partial \hat{a}_t}=-h_t\Gamma_t f(\hat{a}_t),\quad \frac{\partial A_t}{\partial \hat{a}_t}=-h_t\Gamma_t f(\hat{a}_t)a_t^*+h_t\Gamma_t(1-F(\hat{a}_t))\frac{\partial a_t^*}{\partial \hat{a}_t},$$

and

$$\frac{\partial a_t^*}{\partial \hat{a}_t}=\frac{f(\hat{a}_t)}{1-F(\hat{a}_t)}(a_t^*-\hat{a}_t).$$

That is,

$$\begin{aligned}\frac{\partial A_t}{\partial \hat{a}_t}&=-h_t\Gamma_t f(\hat{a}_t)a_t^*+h_t\Gamma_t f(\hat{a}_t)(a_t^*-\hat{a}_t)\\&=-h_t\Gamma_t f(\hat{a}_t)\hat{a}_t.\end{aligned}$$

The first order condition can thus be rewritten as

$$-[(1-\omega)(\bar{x}z_t-\chi)+\beta E_t[v_n(\hat{n}_t,A_t,z_{t+1},s_t)]+\beta E_t[v_s(n_t,A_t,z_{t+1},s_t)]]h_t$$
$$-[(1-\omega)\bar{x}\hat{a}_t+\beta E_t[v_A(n_t,A_t,z_{t+1},s_t)]\hat{a}_t]h_t+\kappa=0. \quad \text{(OE.5)}$$

The envelope condition with respect to A_{t-1} is

$$v_A(n_{t-1},A_{t-1},z_t,s_{t-1})=(1-\omega)\bar{x}(1-\delta)+\beta(1-\delta)E_t[v_A(n_t,A_t,z_{t+1},s_t)]. \quad \text{(OE.6)}$$

The envelope condition with respect to n_{t-1} is

$$v_n(n_{t-1},A_{t-1},z_t,s_{t-1}) = (1-\omega)\left((\bar{x}z_t-\chi)\frac{\partial n_t}{\partial n_{t-1}}+\bar{x}\frac{\partial A_t}{\partial n_{t-1}}\right)+\kappa(1-F(\hat{a}_t))(1-\delta)$$
$$+\beta E_t[v_A(n_t,A_t,z_{t+1},s_t)]\frac{\partial A_t}{\partial n_{t-1}}+\beta E_t[v_n(n_t,A_t,z_{t+1},s_t)]\frac{\partial n_t}{\partial n_{t-1}}$$
$$+\beta E_t[v_s(n_t,A_t,z_{t+1},s_t)]\frac{\partial s_t}{\partial n_{t-1}}, \quad \text{(OE.7)}$$

with

$$\frac{\partial n_t}{\partial n_{t-1}} = (1-\delta)(1-h_t(1-F(\hat{a}_t))), \quad \text{and} \quad \frac{\partial A_t}{\partial n_{t-1}} = -(1-\delta)h_t(1-F(\hat{a}_t))a_t^*, \quad \text{(OE.8)}$$

as well as

$$\frac{\partial s_t}{\partial \hat{n}_{t-1}} = (1-\delta)(1-h_t(1-F(\hat{a}_t))). \quad \text{(OE.9)}$$

Lastly, the envelope condition with respect to s_{t-1} is

$$v_s(n_{t-1},A_{t-1},z_t,s_{t-1}) = (-\delta\gamma)(1-F(\hat{a}_t)\Big(h_t\Big[(1-\omega)(\bar{x}(z_t+a_t^*)-\chi)$$
$$+\beta E_t[v_n(n_t,A_t,z_{t+1},s_t)]+\beta E_t[v_s(n_t,A_t,z_{t+1},s_t)]+\beta E_t[v_A(n_t,A_t,z_{t+1},s_t)]a_t^*\Big]-\kappa\Big)$$
$$+\beta E_t[v_s(n_t,A_t,z_{t+1},s_t)]\gamma \quad \text{(OE.10)}$$

Inserting the expressions in equations (OE.8) and (OE.9) into equation (OE.7) gives

$$v_n(n_{t-1},A_{t-1},z_t,s_{t-1}) = (1-\delta)\Big\{(1-\omega)\left((\bar{x}z_t-\chi)(1-h_t(1-F(\hat{a}_t))-h_t(1-F(\hat{a}_t))\bar{x}a_t^*\right)$$
$$+\kappa(1-F(\hat{a}_t))-\beta E_t[v_A(n_t,A_t,z_{t+1},s_t)]h_t(1-F(\hat{a}_t))a_t^*+\beta E_t[v_n(n_t,A_t,z_{t+1},s_t)]$$
$$\times(1-h_t(1-F(\hat{a}_t)))+\beta E_t[v_s(n_t,A_t,z_{t+1},s_t)](1-h_t(1-F(\hat{a}_t)))\Big\}. \quad \text{(OE.11)}$$

Rearranging

$$v_n(n_{t-1},A_{t-1},z_t,s_{t-1}) = (1-\delta)\Big\{(1-\omega)(\bar{x}z_t-\chi) - (1-F(\hat{a}_t))\Big[h_t\Big((1-\omega)(\bar{x}(z_t+a_t^*)-\chi)$$
$$+\beta E_t[v_A(n_t,A_t,z_{t+1},s_t)]a_t^* + \beta E_t[v_n(n_t,A_t,z_{t+1},s_t)] + \beta E_t[v_s(n_t,A_t,z_{t+1},s_t)]\Big) - \kappa\Big]$$
$$+\beta E_t[v_n(n_t,A_t,z_{t+1},s_t)] + \beta E_t[v_s(n_t,A_t,z_{t+1},s_t)]\Big\}. \quad \text{(OE.12)}$$

Conjecture that $v_n(n_{t-1},A_{t-1},z_t,\Upsilon_t)$ only depends on z_t and a_t, and that $v_A(n_{t-1},A_{t-1},z_t,\Upsilon_t)$ is a constant. That is $v_n(z_t,a_t)$, and v_A. Then define $W(z,a,a')$ as

$$(1-\delta)W(z,a,a') = v_n(z,a') + v_A a.$$

Using this definition we can rewrite equation (OE.12) as

$$W(z_t,a_t^*,a_t^*) = (1-\omega)(\bar{x}(z_t+a_t^*)-\chi) - (1-F(\hat{a}_t))\Big[h_t\Big((1-\omega)(\bar{x}(z_t+a_t^*)-\chi)$$
$$+\beta(1-\delta)E_t[W(z_{t+1},a_t^*,a_{t+1}^*)] + \beta E_t[v_s(n_t,A_t,z_{t+1},s_t)]\Big) - \kappa\Big]$$
$$+\beta(1-\delta)E_t[W(z_{t+1},a_t^*,a_{t+1}^*)] + \beta E_t[v_s(n_t,A_t,z_{t+1},s_t)]. \quad \text{(OE.13)}$$

Define $J_t(a)$ as

$$J_t(a) = (1-\omega)(\bar{x}(z_t+a)-\chi) + \beta(1-\delta)E_t[J_{t+1}(a)] + \beta\delta E_t[\hat{J}_{t+1}^U], \quad \text{(OE.14)}$$

J_t^U as

$$J_t^U = J_t(a) - W(z,a,a').$$

and $\hat{J}_t^U$ (implicitly) as

$$v_s(n_{t-1},A_{t-1},z_t,\Upsilon_t) = \delta(\hat{J}_t^U - J_t^U). \quad \text{(OE.15)}$$

Using these relations we can rewrite equation (OE.13) as

$$J_t(a_t^*) - J_t^U = (1-\omega)(\bar{x}(z_t + a_t^*) - \chi) - (1 - F(\hat{a}_t))\Big[h_t\Big((1-\omega)(\bar{x}(z_t + a_t^*) - \chi)$$
$$+ \beta(1-\delta)E_t[J_{t+1}(a_t^*) - J_{t+1}^U] + \beta\delta E_t[\hat{J}_{t+1}^U - J_{t+1}^U]\Big) - \kappa\Big]$$
$$+ \beta(1-\delta)E_t[J_{t+1}(a_t^*) - J_{t+1}^U] + \beta\delta E_t[\hat{J}_{t+1}^U - J_{t+1}^U],$$

or simply

$$J_t^U = (1 - F(\hat{a}_t))\Big[h_t((1-\omega)(J_t - \beta E_t[J_{t+1}^U]) - \kappa\Big] + \beta E_t[J_{t+1}^U]. \tag{OE.16}$$

Moreover equation (OE.10) can be rewritten as

$$v_s(n_{t-1}, A_{t-1}, z_t, s_{t-1}) = (-\delta\gamma)(1 - F(\hat{a}_t))\Big[h_t((1-\omega)(J_t - \beta E_t[J_{t+1}^U]) - \kappa\Big]$$
$$+ \beta E_t[v_s(n_t, A_t, z_{t+1}, s_t)]\gamma,$$

or

$$v_s(n_{t-1}, A_{t-1}, z_t, s_{t-1}) = -\delta\gamma J_t^U + \beta\delta\gamma E_t[J_{t+1}^U] + \beta E_t[v_s(n_t, A_t, z_{t+1}, s_t)]\gamma.$$

Thus

$$\hat{J}_t^U = (1-\gamma)J_t^U + \gamma\beta E_t[\hat{J}_{t+1}^U]. \tag{OE.17}$$

Lastly, using the same definitions, we can rewrite the first order condition as

$$\left[(1-\omega)(\bar{x}(z_t + \hat{a}_t - \chi) + \beta(1-\delta)E_t[W(z_{t+1}, \hat{a}_t, a_{t+1}^*)] + \beta E_t[v_s(n_t, A_t, z_{t+1}, s_t)]\right] h_t - \kappa = 0,$$

or

$$
\begin{aligned}
\kappa &= h_t \left[(1-\omega)(\bar{x}(z_t+\hat{a}_t-\chi)+\beta(1-\delta)E_t[W(z_{t+1},\hat{a}_t,a^*_{t+1})]+\beta E_t[v_s(n_t,A_t,z_{t+1},s_t)]\right], \\
&= h_t \left[(1-\omega)(\bar{x}(z_t+\hat{a}_t-\chi)+\beta(1-\delta)E_t[J_{t+1}(\hat{a}_t)-J^U_{t+1}]+\beta\delta E_t[\hat{J}^U_{t+1}-J^U_{t+1}]\right], \\
&= h_t(J_t(\hat{a}_t)-\beta E_t[J^U_{t+1}]). \qquad \text{(OE.18)}
\end{aligned}
$$

Equations (OE.14), (OE.16), (OE.17), and (OE.18) corresponds to the main equations in section OD.1. Thus, the framework can be interpreted either as an infinitely-lived representative firm or as firm-worker pairs.

As indicated already in the opening paragraph of this section, we have shown that a representative, multi-worker firm representation is admissable for the model analyzed in section OD.1 of this online appendix. Relative to the model considered in the main text, equation (OE.14) includes a continuation term $\beta\delta E_t[\hat{J}^U_{t+1}]$ that is absent in the main text (cf. equation (15)) and which reflects the fact that projects continue to have a positive value upon separation. Section OD.1 demonstrated that for $\gamma \to 1$, the two models imply qualitatively and quantitatively similar impulse responses for an uncertainty shock. An exact equivalence to equation (15) can be derived by supposing that a fraction of filled projects, upon exogenous destruction, becomes obsolete and is lost to the operating firm forever; a different, new firm then has the opportunity to launch an equal measure of projects.[OE.3]

[OE.3] As already mentioned in footnote OE.2, the proof is quite lengthy but is available upon request.

Appendix OF Robustness exercises

This appendix section reports the results of several robustness checks regarding the novel search-and-matching (SaM) model with a finite mass of entrepreneurs and firm-specific productivity.

OF.1 Stochastic vs. non-stochastic hiring specification

The model in section 4.1 assumes that in any period t, an unmatched entrepreneur can post a single vacancy, which costs κ units of final goods. The vacancy turns into a match with probability h_t. Otherwise the vacancy remains unfilled and the entrepreneur faces a new decision about whether to remain on the sidelines or post a vacancy at the beginning of the following period. We will refer to this specification as "stochastic hiring." As it is probably the most common way of formulating the SaM model, used in particular also by Leduc and Liu (2016, esp. their eqn. 22), we adopt it as our baseline. An alternative specification supposes that the entrepreneur posts $1/h_t$ vacancies, each costing κ units of the final good, and then creates one job with certainty. We will refer to this case as "non-stochastic hiring."

In the canonical SaM model with risk-neutral entrepreneurs, the two specifications yield isomorphic equilibrium conditions; only the suggested interpretations potentially differ. Either way, in equilibrium, labor market tightness — and, hence, h_t — adjusts such that the expected profit from a new job is equal to the expected cost of hiring a worker. The value of waiting is zero by construction. Under stochastic hiring, it is most natural to write $h_t J_t = \kappa$, meaning that in equilibrium the value of a match discounted by the probability of a vacancy being filled is equal to the fixed cost of posting that vacancy. Under non-stochastic hiring, writing $J_t = \kappa(1/h_t)$ is more natural, in which case equilibrium requires the value of a match to be equal to the expected cost of posting a vacancy until it is filled.

In our modified framework, entrepreneurs are also risk neutral. Nevertheless, the quantitative model outcomes are somewhat different for these two different hiring specifications. In brief, the reason is that under stochastic hiring, expected profits from waiting now and posting a vacancy later

are affected by the hiring probability covarying negatively with the match value; the same is not true under non-stochastic hiring. We next discuss these properties in some more detail.

The intuition is most easily described with reference to the two-period model. Recall that in our baseline, the indifference condition is (cf. equation (OC.6))

$$-\kappa + h_1(1+\widehat{a}_1) = E_1\left[p_2(-\kappa + h_2(z_2 + a_2^*))\right].$$

In contrast, when entrepreneur can post multiple vacancies, the condition is

$$-\kappa/h_1 + (1+\widehat{a}_1) = E_1\left[p_2(-\kappa/h_2 + (z_2 + a_2^*))\right].$$

Now, the level of idiosyncratic productivity above which an entrepreneur would rather enter than wait (i.e., not produce at all in the two-period setting), $\widehat{a}_2$, and, hence, the conditional expected value of a draw, a_2^*, are the same across the alternative hiring cost specifications. Instead, the key difference between these two equations is that in the former case, the *expectation* on the right-hand side, conditional on period-1 information, includes a product term involving h_2 and z_2, so that their negative co-movement lowers the value of waiting. By contrast, since under non-stochastic hiring $1/h_2$ multiplies a term that is invariant to uncertainty regarding z_2 (viz., the fixed costs κ), this same mechanism is not operative.[OF.1]

The upper row of panels in Figure OF.1 uses the graphical apparatus set out in section OC to describe the value of waiting due to aggregate uncertainty under the two alternative specifications. As is clearly visible, the expected value of waiting – the difference between the increase in profits in an expansion and the decrease in profits in a recession when integrating over all possible productivity draws above the respective cutoffs – is greater under the alternative, non-stochastic hiring specification. The reason is that the benefit from a higher idiosyncratic productivity draw in an expansion is not scaled down by greater congestion that lowers the probability of that draw

[OF.1] For completeness, notice that the division of κ by h_2 under non-stochastic hiring affects the expected value of hiring costs in the presence of aggregate uncertainty. We consider a quantitative assessment of the two specifications below.

Figure OF.1: Stochastic vs. non-stochastic hiring specification

(a) 2-period model: stochastic hiring

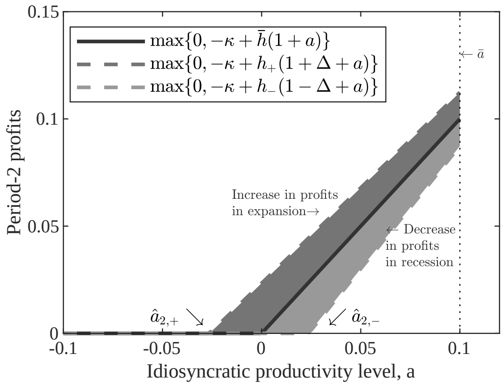

(b) 2-period model: non-stochastic hiring

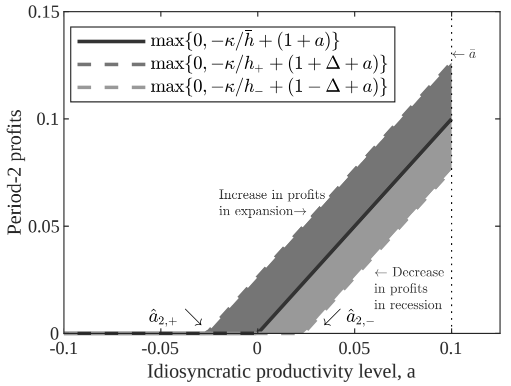

(c) Pure uncertainty IRFs in the full model, $\sigma_a = 0.001$

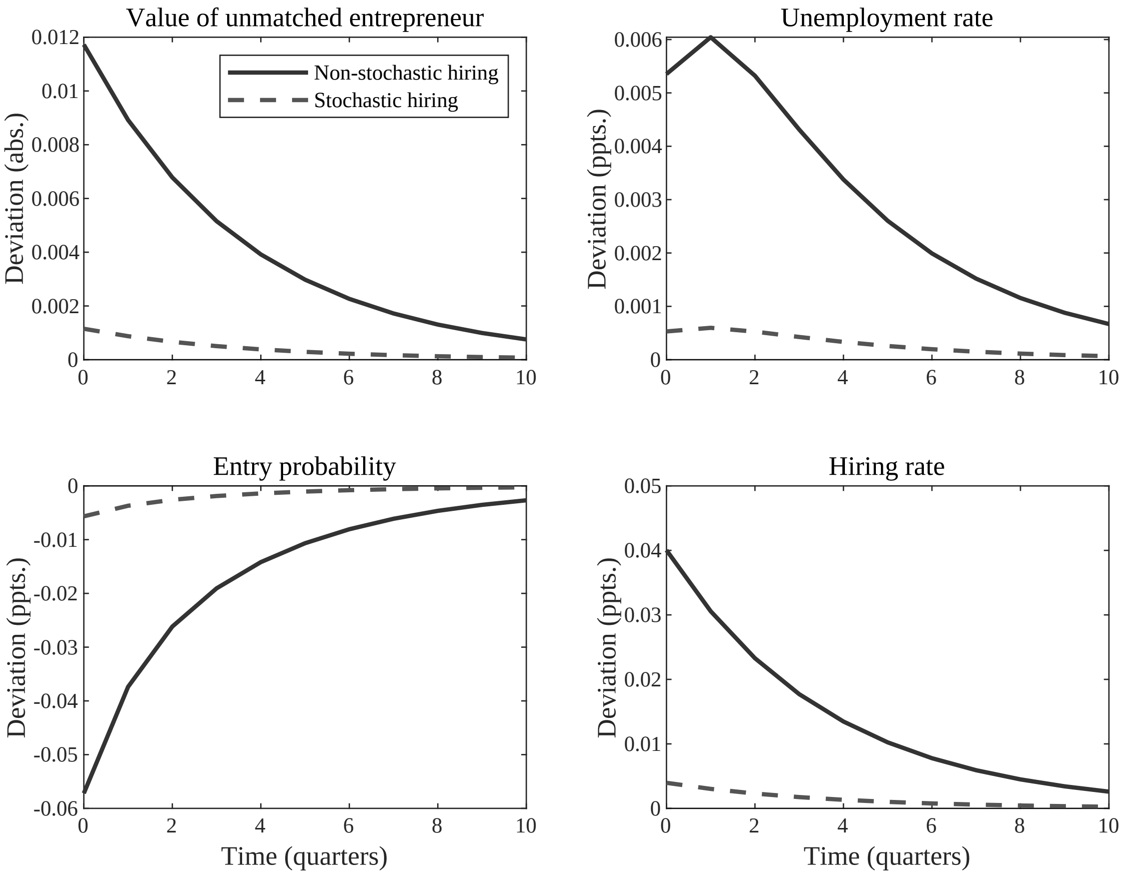

Notes: Panels **??** and OF.1b show how the value of waiting is determined in a stylized, two-period setting. The left panel corresponds to Figure OC.1c and relies on the "stochastic hiring" assumption. The right panel instead captures the "non-stochastic" specification. To make the visual comparison straightforward, the steady-state hiring rate, h, is set equal to one; the no-aggregate-uncertainty line is then the same for both specifications. For the full, infinite-horizon model, Panel OF.1c plots the usual pure uncertainty IRFs to a unit-increase in $\varepsilon_{\sigma,t}$.

actually yielding profits in a filled match. The opposite holds in a recession, but then these relatively higher probability of hiring boosts expected profits only over a smaller range of productivity draws. (This is the flipside of the argument presented in section OC.2, of course.) Thus, the comparison of alternative hiring specifications informs us that firm size potentially matters for the transmission of uncertainty shocks even under constant returns to scale in production, insofar as the option-value effect will be stronger when an entrepreneur can post several vacancies instead of just one.

How do these different specifications shape the predictions of the full, infinite-horizon model for the effects on economic activity of an anticipated increase in volatility?[OF.2] The solid line in Figure OF.1c graphs the pure uncertainty IRFs for the value of an unmatched entrepreneur, unemployment, the entry probability and the hiring rate, when $\sigma_a = 0.001$ for the non-stochastic hiring specification. The dashed line represents the stochastic hiring benchmark. In either model, the recalibration procedure described in section 4.2 is applied, which facilitates comparison. Consistent with the reasoning developed in the context of the two-period model, the uncertainty effects are several magnitudes larger given the non-stochastic hiring specification than in the baseline. The analysis thus indicates that the results in the main text, which are based on the stochastic hiring assumption, represent a conservative, lower bound for the effects of uncertainty shocks.

[OF.2] To be explicit, under either specification the equation pinning down the cutoff level $\widehat{a}$ for which an entrepreneur is indifferent between going to the matching market is

$$-\kappa/h_t = J_t(\widehat{a}_t) - \beta E_t[J^U_{t+1}].$$

But under the non-stochastic hiring specification the beginning-of-period t of an unmatched entrepreneur is given by

$$J^U_t = \beta E_t[J^U_{t+1}] + p_t\left(-\frac{\kappa}{h_t} + (J_t(a^*_t) - \beta E_t[J^U_{t+1}])\right),$$

with steady-state value $J^U = \frac{p(-\kappa/h+J(a^*))}{1-\beta(1-p)}$. Previously, we had

$$J^U_t = \beta E_t[J^U_{t+1}] + p_t\left(-\kappa + h_t(J_t(a^*_t) - \beta E_t[J^U_{t+1}])\right),$$

and $J^U = \frac{p(-\kappa+h*J(a^*))}{1-\beta(1-ph)}$.

OF.2 Normal distribution

Our baseline specification of the model with heterogeneity in entrepreneurial productivity assumes that firm-level productivity has a uniform distribution in the interval $[-\sqrt{3}\sigma_a, \sqrt{3}\sigma_a]$. Methodologically, our baseline assumption has the advantage that in the recalibration step (cf. section 4.2) we can analytically solve for the elasticity of labor market tightness with respect to productivity. In addition, the uniform distribution ensured consistency between the full model and the analytical, two-period model presented in section OC. This appendix section shows that the results are highly comparable when, instead, firm-level productivity draws comes from a normal distribution, that is, when $a \sim \mathcal{N}(0, \sigma_a^2)$.

Focusing on the implications of firm-level dispersion in the absence of aggregate uncertainty first, the solid lines in the upper panels of Figure OF.2 report the steady-state value of an unmatched entrepreneur, J^U, as well as the unemployment rate as a function of σ_a. To ease comparison, the dashed line repeats the results under a uniform distribution. Unsurprisingly, the qualitative patterns are the same. The only model equations that are directly affected by functional choices for $F(a)$ are the expressions for $p = \text{prob}[a \geq \widehat{a}]$ and $a^* = E[a \geq \widehat{a}]$. In either case, greater values of σ_a increase the value of remaining unmatched, as doing so preserves the option of getting another, better draw in the future. Without recalibrating the model parameters, this effect lowers lower vacancy posting and, hence, increases steady-state unemployment.[OF.3]

Next, Figure OF.2b reports the IRFs for an increase in anticipated future aggregate volatility under the two alternative assumptions. Again the results are highly comparable; the effect sizes are just slightly more pronounced under the assumption of a uniform distribution. These quantitative differences further illustrate the point made in section 4.1 that aggregate uncertainty shocks have a smaller impact for larger values of σ_a when the hiring rate is endogenous.[OF.4] With a 'tighter' distribution, here in the sense of the normal distribution having greater kurtosis than the uniform,

[OF.3] Quantitatively, the strength of this mechanism increases slightly more with σ_a when assuming a normal distribution. For even though idiosyncratic uncertainty raises the conditional expected value of a draw by more under a uniform distribution, the mass of marginal entrepreneurs (i.e., near the indifference point) is greater under a normal distribution.

[OF.4] This result assumes, of course, that in steady state the cutoff is at or near the center of the normal distribution.

entrepreneurs are more similar. Accordingly, we are closer to the benchmark case where profits are completely eroded due to variations in the hiring rate due to (expected) movements in aggregate productivity.

Figure OF.2: Uncertainty effects with normal distribution for firm-level productivity

(a) Steady-state effects of greater idiosyncratic dispersion

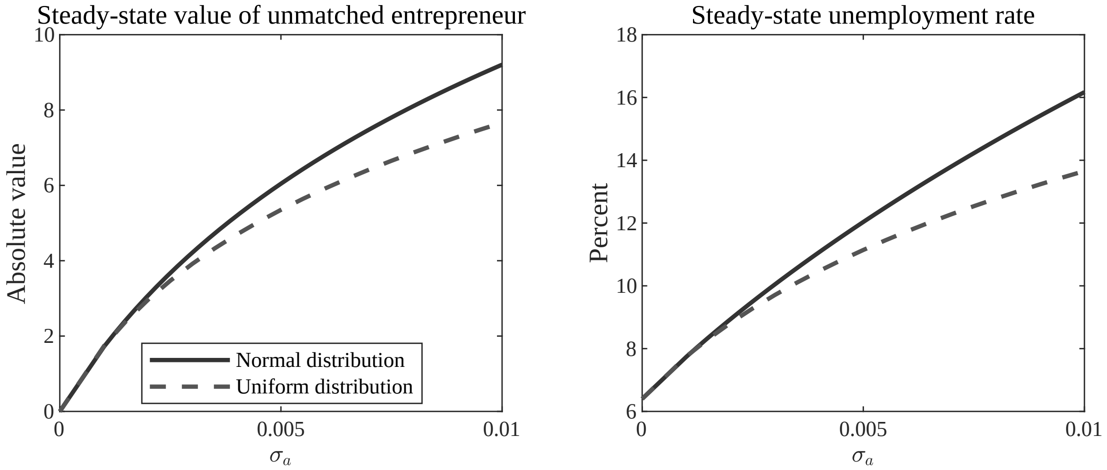

(b) Pure uncertainty IRFs ($\sigma_a = 0.001$)

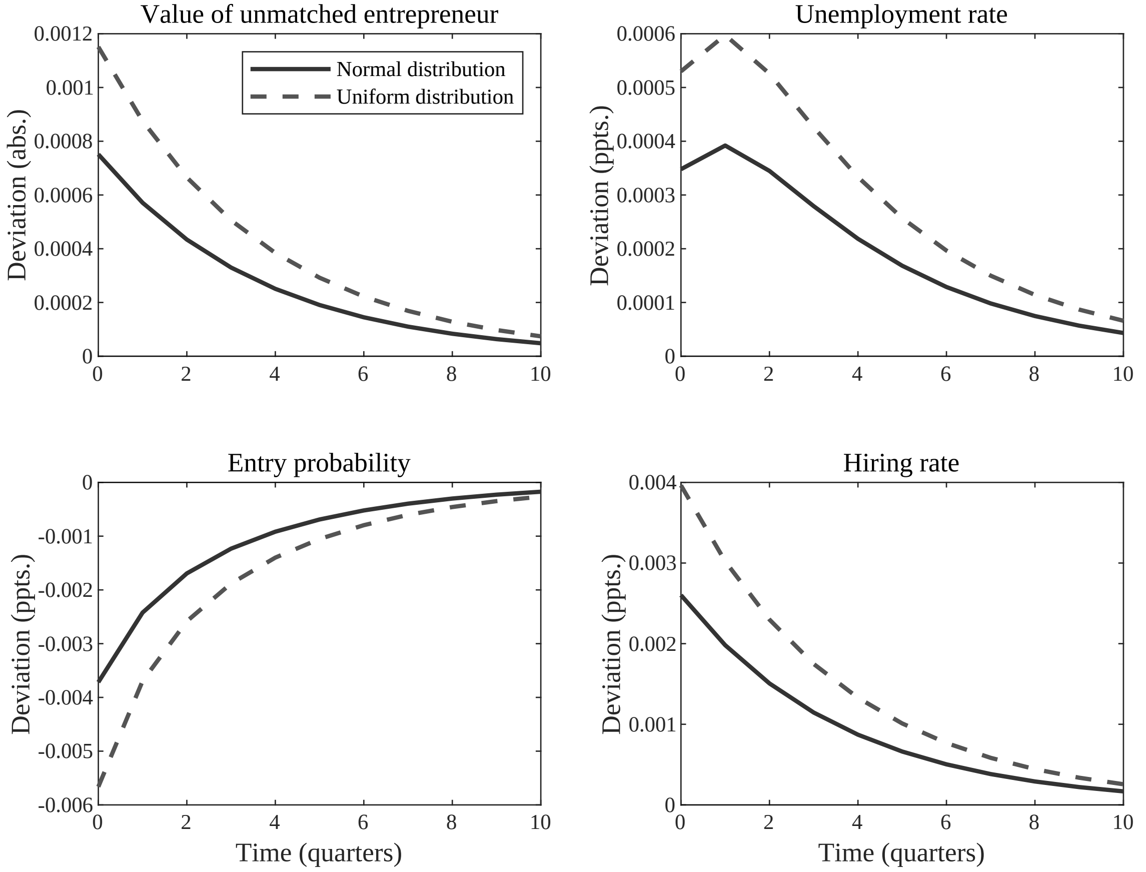

Notes: The two panels in Figure OF.2a describe the steady-state effects of increasing firm-level dispersion, as measured by σ_a, assuming a normal (solid line) or uniform (dashed line) respectively. The panels in Figure OF.2b reports pure uncertainty IRFs to a unit-increase in $\varepsilon_{\sigma,t}$ under these two assumptions, imposing $\sigma_a = 0.001$ and $P = 0.5$. In the latter case, the model parameters are recalibrated following the procedure described in section 4.2.

OF.3 Different degree of cross-sectional dispersion

Figure 5 indicated pure uncertainty and total volatility IRFs conditional on the standard deviation of idiosyncratic productivity shocks, σ_a, being equal to 0.003. Figure OF.3 reports the same IRFs when $\sigma_a = 0.001$ instead. A comparison of the two figures reveals that the magnitude of uncertainty effects is generally larger for the higher value of σ_a, in line with what is shown in figure 5 for the unemployment rate, specifically. Interestingly, the composition effect – following a volatility shock the average productivity of active firms initially rises – is now sufficiently strong for the total volatility effect on output to reach positive territory, albeit only very briefly, before the negative impact of rising unemployment kicks in.

Figure OF.3: Uncertainty effects in SaM model with cross-sectional dispersion; $\sigma_a = 0.001$

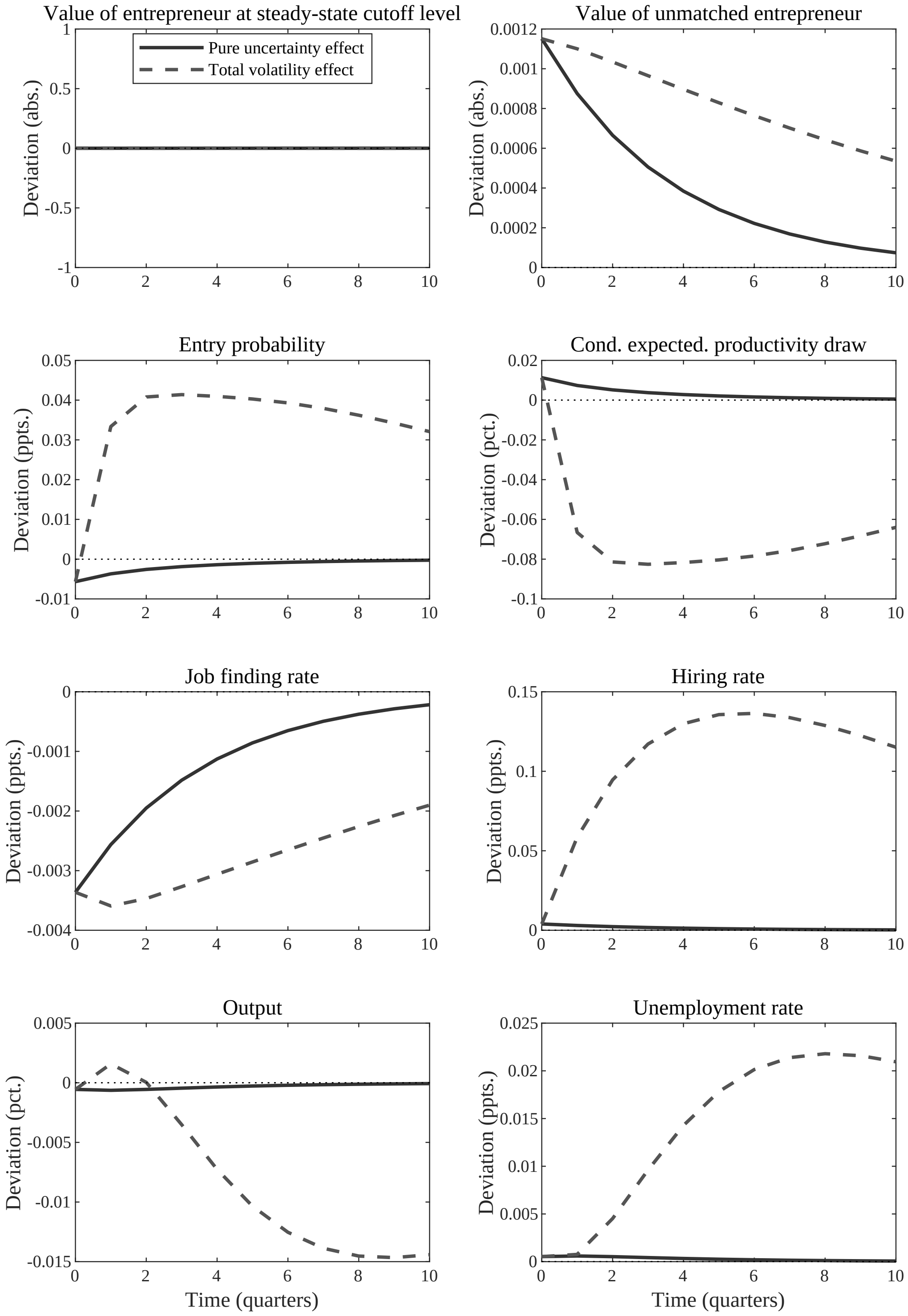

Notes: The "total volatility" IRFs plot the change in the period-0 expected values of the indicated variables in response to a unit-increase in $\varepsilon_{\sigma,t}$. The model parameters are recalibrated following the procedure described in section 4.2. In particular, $\chi = 0.682$, $\omega = 0.885$, and $\bar{z} = 0.999$.

OF.4 Different steady-state entry probability

In the baseline analysis, we calibrated the model such that in steady state half of the available entrepreneurs actually post a vacancy. As noted in the main text, this choice has a number of practical advantages. For completeness, we report the results of a robustness exercise in which the steady-state value for p is 0.2 instead of 0.5.

Figure OF.4 compares the pure uncertainty IRFs for the value of an unmatched entrepreneur and unemployment, as well as the entry probability and the hiring rate, when $\sigma_a = 0.001$ for both $p = 0.2$ (solid line) and $p = 0.5$ (dashed line; as in figure OF.3).[OF.5] Clearly, the value of waiting due to greater anticipated uncertainty rises by less in the latter case, and accordingly unemployment worsens by less also. The reason is that a lower steady-state entry probability is associated with a larger mass of potential entrepreneurs, Υ. As such, for any given degree of firm-level dispersion we are closer to the benchmark SaM model with an infinite mass of potential entrepreneurs. In that latter model, the option value of waiting, and thus also pure uncertainty effects given risk neutrality and linear wages, are zero. In more economic terms, with more potential entrepreneurs, adjustments in the hiring rate in both good and bad productivity states are sufficient to leave expected profits nearly invariant to changed in anticipated volatility.

Imposing a lower steady state probability of entry means that the admissable range of σ_a is wider when recalibrating the model as described in section 4.2 (as the worker bargaining power parameter ω hits the lower limit of zero for higher values of σ_a when p is lower, that is, when the mass of potential entrepreneurs is greater). In this spirit, the solid line in Figure OF.4b indicates the impact effect of an uncertainty shock as a function of σ_a when $p = 0.2$. The dashed line refers to the same statistic for a fixed value $\sigma_a = 0.001$ but with $p = 0.5$. This analysis makes clear that for a lower steady-state entry probability, the option-value effect due to anticipated greater future volatility matches that obtained with a higher steady-state entry probability for a greater degree of firm-level dispersion.

[OF.5] Given our recalibration procedure, steady-state unemployment is the same across the different specifications.

Figure OF.4: Uncertainty effects with lower steady-state entry probability

(a) Pure uncertainty IRFs ($\sigma_a = 0.001$)

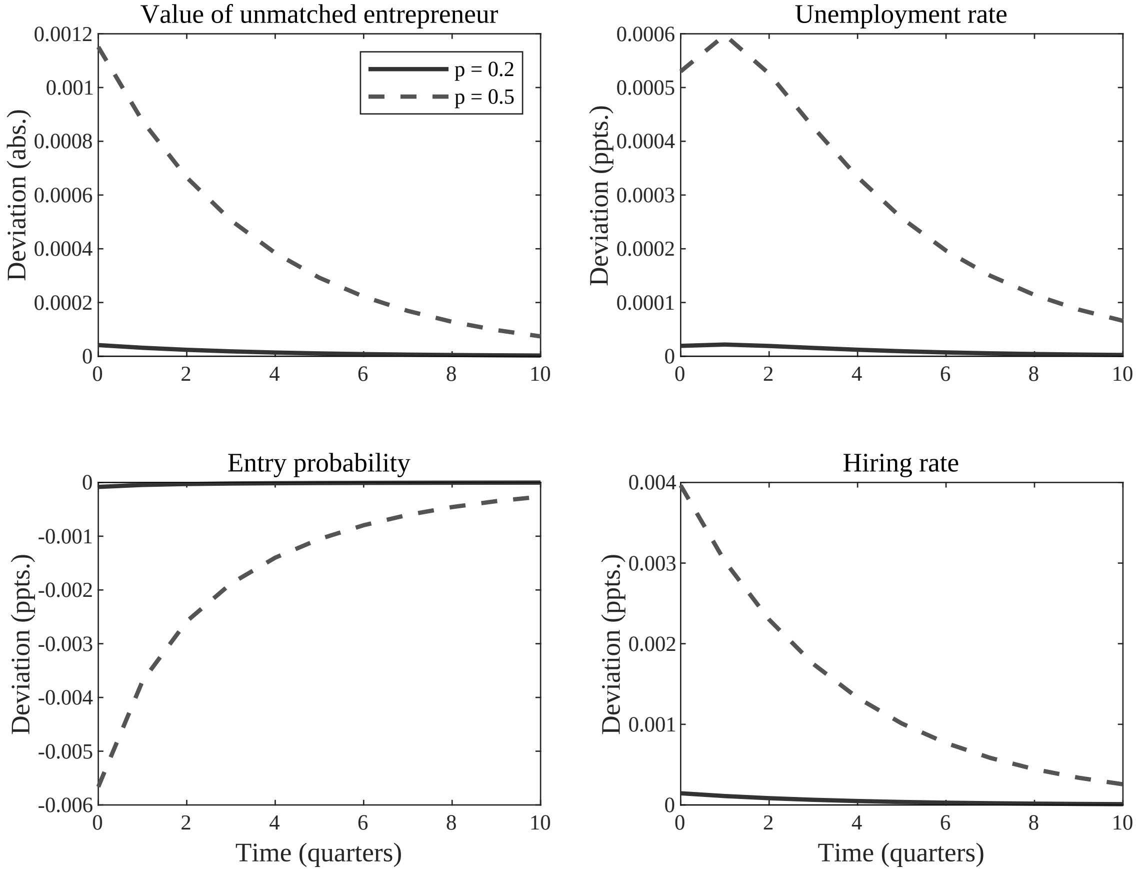

(b) Impact effects of uncertainty shock for different σ_a values ($p = 0.2$)

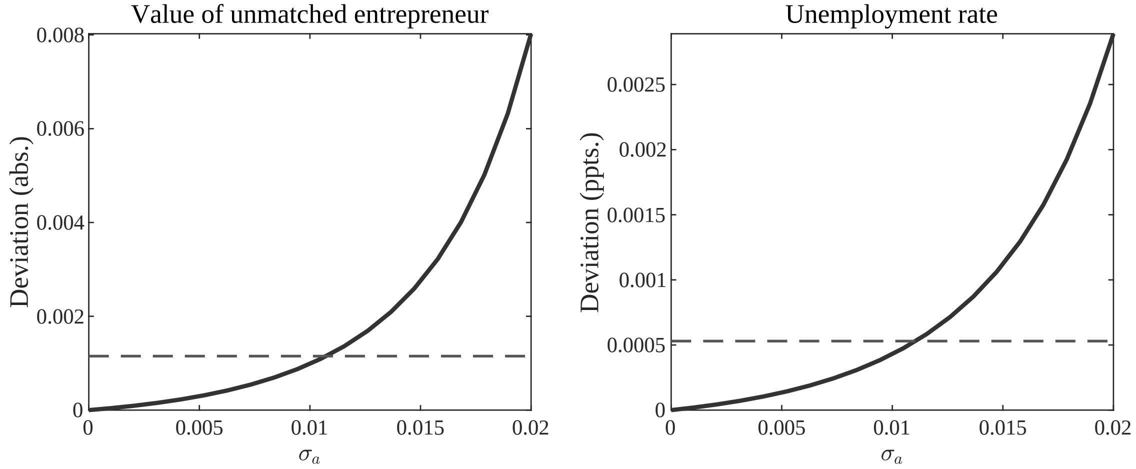

Notes: The upper panel reports pure uncertainty IRFs to a unit-increase in $\varepsilon_{\sigma,t}$, assuming that $\sigma_a = 0.001$ and that the steady-state entry probability is either $p = 0.2$ (solid line) or $p = 0.5$ (dashed line). The bottom panel plots the impact effect in the case of $p = 0.2$ as a function of σ_a (solid line). Here, the dashed line describes the impact effect for a fixed value of $\sigma_a = 0.001$ and with $p = 0.5$. The model parameters are recalibrated following the procedure described in section 4.2.

References for online appendix

Freund, L. B. and Rendahl, P. (2020). Unexpected Effects: Uncertainty, Unemployment, and Inflation. *CEPR Discussion Paper DP14690*.

Leduc, S. and Liu, Z. (2016). Uncertainty Shocks are Aggregate Demand Shocks. *Journal of Monetary Economics*, **82**, 20–35.

Petrosky-Nadeau, N., Zhang, L., and Kuehn, L.-A. (2018). Endogenous Disasters. *American Economic Review*, **108**(8), 2212–2245.
