# Supplementary material for: Volatile hiring: uncertainty in search and matching models
Source: J Monet Econ. 2021 Oct;123:1–18. doi: 10.1016/j.jmoneco.2021.07.008 (PMC8547261; doi:10.1016/j.jmoneco.2021.07.008)
Supplement: Supplementary file 2 [file mmc2.zip › ReplicationKit/Appendices/Appendix_EndogenousMassEntrepreneurs/Output/fig_App_SaM_HetFirm_EndoMass_Isoelastic_Comparison.pdf]

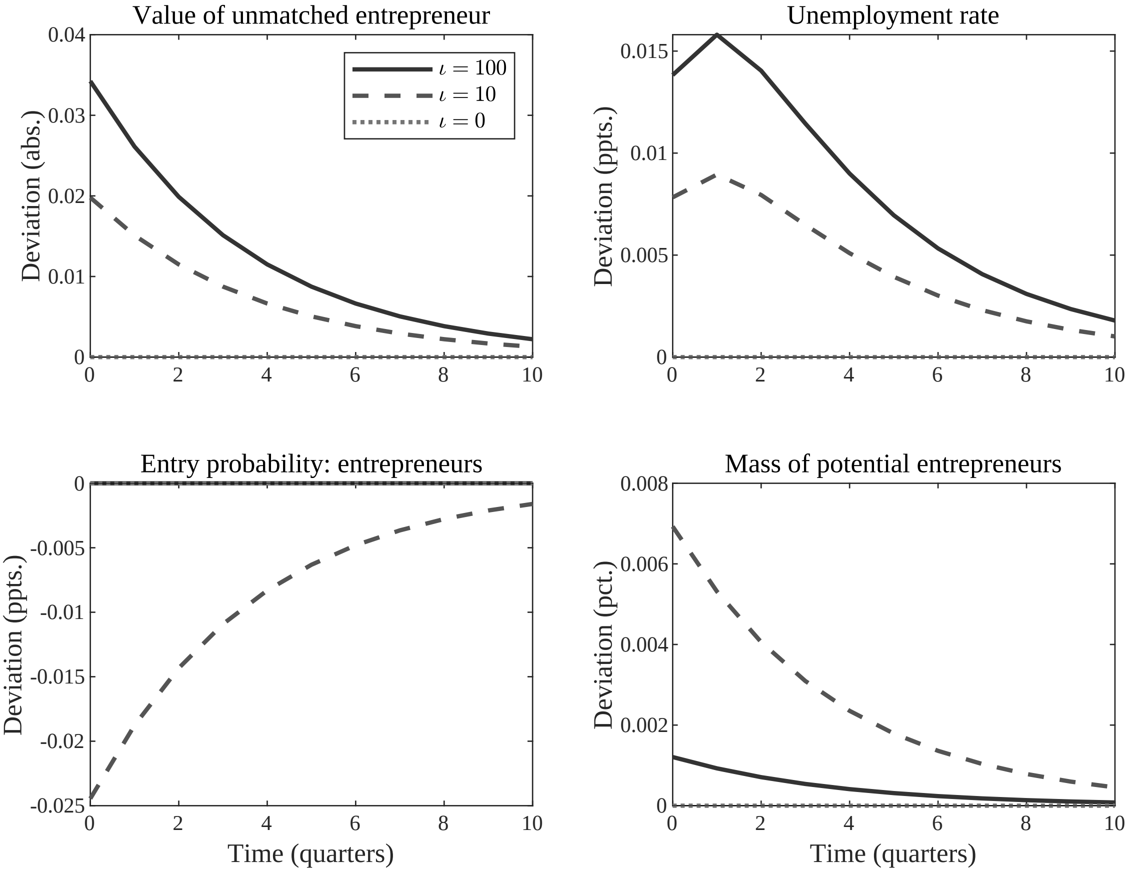

Value of unmatched entrepreneur
Unemployment rate
Entry probability: entrepreneurs
Mass of potential entrepreneurs
$\iota = 100$
$\iota = 10$
$\iota = 0$
Deviation (abs.)
Deviation (ppts.)
Deviation (ppts.)
Deviation (pct.)
Time (quarters)
Time (quarters)
0.04
0.03
0.02
0.01
0
0.015
0.01
0.005
0
-0.005
-0.01
-0.015
-0.02
-0.025
0.008
0.006
0.004
0.002
0
2
4
6
8
10
