# Supplementary material for: Volatile hiring: uncertainty in search and matching models
Source: J Monet Econ. 2021 Oct;123:1–18. doi: 10.1016/j.jmoneco.2021.07.008 (PMC8547261; doi:10.1016/j.jmoneco.2021.07.008)
Supplement: Supplementary file 2 [file mmc2.zip › ReplicationKit/Appendices/Appendix_NormalDistribution/Output/Figures/fig_SaMOptionValue_NormalDist_p05_sigmaa001_Recalib.pdf]

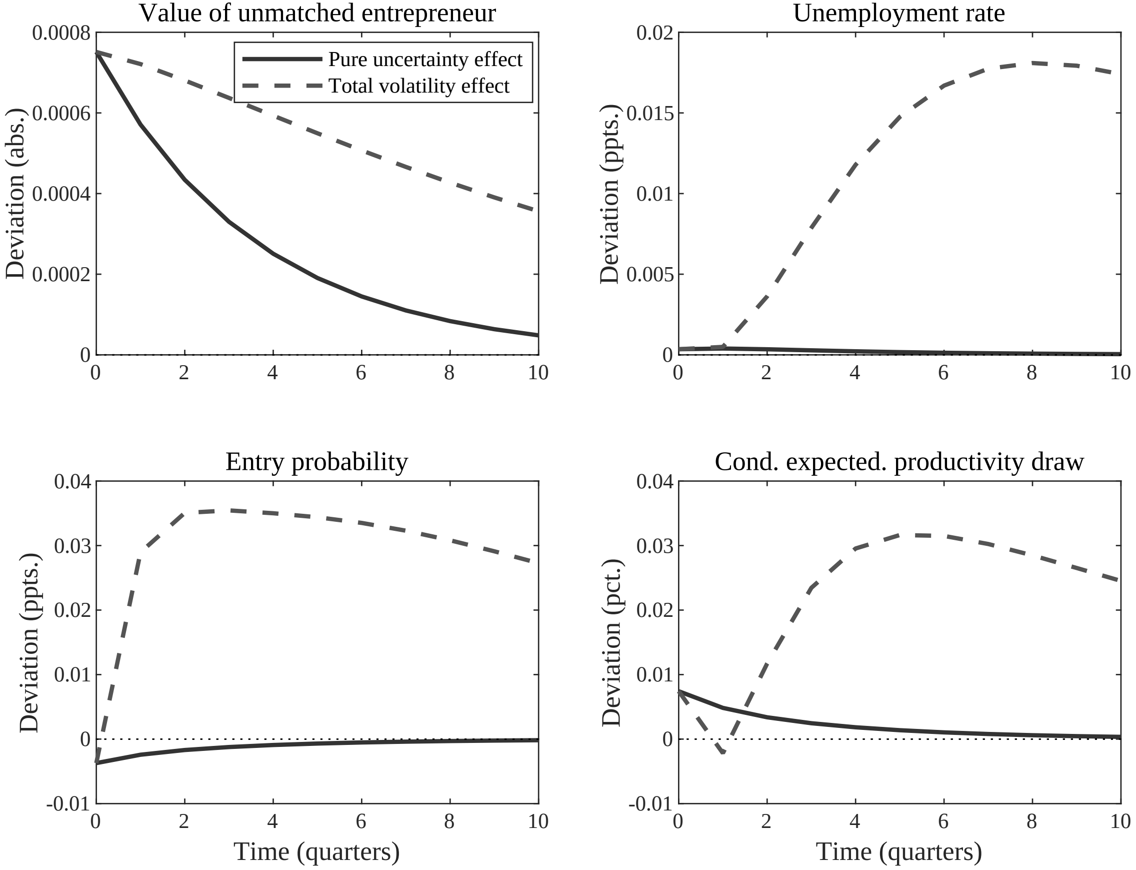

Value of unmatched entrepreneur
Pure uncertainty effect
Total volatility effect
Deviation (abs.)
0.0008
0.0006
0.0004
0.0002
0
0 2 4 6 8 10
Unemployment rate
Deviation (ppts.)
0.02
0.015
0.01
0.005
0
0 2 4 6 8 10
Entry probability
Deviation (ppts.)
0.04
0.03
0.02
0.01
0
-0.01
0 2 4 6 8 10
Time (quarters)
Cond. expected. productivity draw
Deviation (pct.)
0.04
0.03
0.02
0.01
0
-0.01
0 2 4 6 8 10
Time (quarters)
