# Supplementary material for: Volatile hiring: uncertainty in search and matching models
Source: J Monet Econ. 2021 Oct;123:1–18. doi: 10.1016/j.jmoneco.2021.07.008 (PMC8547261; doi:10.1016/j.jmoneco.2021.07.008)
Supplement: Supplementary file 2 [file mmc2.zip › ReplicationKit/DHFR_VolatileHiring_Replication_Documentation.pdf]

Replication files for: "Volatile Hiring: Uncertainty in Search and Matching Models"*

Wouter Den Haan
London School of Economics, CEPR and CFM

Lukas B. Freund
University of Cambridge

Pontus Rendahl
Copenhagen Business School, CEPR and CFM

July 2021

This document describes how to replicate the numerical results in the article "Volatile Hiring: Uncertainty in Search and Matching Models." The accompanying replication codes can be downloaded under this link.[1] The folder structure there and the sections in this document match in an obvious way, hence we do not always indicate the full path for every file we refer to.

Any references to figures or sections that are not preceded by a capital letter refer to the main article, whereas those that are refer to the online appendix.

* *E-mail*: Den Haan: w.denhaan@lse.ac.uk; Freund: lukas.beat.freund@gmail.com; Rendahl: pontus.rendahl@gmail.com. If you find any mistakes or have questions, please contact Lukas Freund.

[1] Please note that these replication codes were written for Matlab 2019b and Dynare 4.4.3. Compatibility with different versions, notably Dynare 4.6 and above, is not guaranteed.

I Benchmark search-and-matching model (Section 3)

As described in the main text, the policy functions of the benchmark search-and-matching model with stochastic volatility are approximated using higher-order perturbation methods, relying on the Dynare package for Matlab (Adjemian *et al.* (2018)). The master file is `main_SaMBaseline.m`. In this file, the user specifies the main settings, including structural parameter values. This file calls the Dynare mod file `dynareSaMBaseline.mod` and plots the relevant IRFs.[2] Here and elsewhere, structural parameters and steady-state values are stored in the structure `sPar`. Generic, design-related settings are stored in the structure `sSettings`.

To elaborate, the model equations are in `dynareSaMBaseline.mod`, with an exact solution for the deterministic steady-state passed on through `dynareSaMBaseline_steadystate.m`. The IRF computations are likewise performed in `dynareSaMBaseline.mod`. In particular, pure uncertainty shocks are computed using a manual, simulations-based approach that involves the following three main steps: (i.) compute the ergodic mean in the absence of shocks (EMAS) by simulating the economy using the policy functions provides by Dynare, starting at the deterministic steady-state, and taking the first point after the burn-in period (during which the economy transitions to the EMAS); (ii.) simulate with an added innovation to the volatility of productivity shocks after the burn-in period; (iii.) compute the IRF as the deviation of variables in that simulation with added innovation from the EMAS.[3] On the other hand, 'traditional' GIRFs are computed using the package provided by Andreasen *et al.* (2018). Note that the resulting "total volatility" IRFs – which represent the agents' rational expectations for the path of variables in the period in which the shock hits – could *also* be obtained by explicitly including in the mod file the expectations for the variables in question.[4]

<u>Exact replication of figures in the paper.</u>

Figure 1. In `dynareSaMBaseline.mod`, set `@#defineOptionWNash=1` (line 20). In `main_SaMBaseline.m`, set `sPar.alpha=0.5` (line 37). Then run `main_SaMBaseline.m`. To also get the formatting exactly right, adjust the vertical axis limits for subplots 4 and 6, respectively, by commenting in and out the prepared specifications in lines 210-212 and 237-238.

Figure 2. In `dynareSaMBaseline.mod`, set `@#defineOptionWNash=0` (line 20). In `main_SaMBaseline.m`, set `sPar.alpha=0.5`. Then run `main_SaMBaseline.m`. To also get the formatting exactly right, adjust the vertical axis limits for subplots 4 and 6, respectively, by commenting in

[2]Notice that section `Manualplotting` of the code pre-selects the variables to be plotted. Commented out at the bottom is an alternative approach that allows the user to select which variables they want to be shown.

[3]For further descriptions, also see Born and Pfeifer (2014) and this blog post. I have benefited from Johannes Pfeifer's extensive and excellent collection of replication codes, notably his implementation of Basu and Bundick (2017).

[4]Please email for further details.

and out the prepared specifications in lines 210-212 and 237-238.

Figure 3. In `dynareSaMBaseline.mod`, set `@#defineOptionWNash=0` (line 20). In `main_SaMBaseline.m`, set `sPar.alpha=0.2`. Then run `main_SaMBaseline.m`. To also get the formatting exactly right, adjust the vertical axis limits for subplots 4 and 6, respectively, by commenting in and out the prepared specifications in lines 210-212 and 237-238.

II Alternative search-and-matching model (Section 4)

Conveniently, our proposed search-and-matching model with a finite mass of entrepreneurs that are heterogeneous in terms of their idiosyncratic productivity levels can still be solved using perturbation methods. The folder `Master_Baseline` provides all codes needed to solve the model and plot the IRFs for an uncertainty shock. The master file is `main_SaMOptionValue.m` and the Dynare file is `dynareSAMOptionValue.mod`.

The Dynare implementation is a straightforward extension of the code for the standard search-and-matching model, the primary modifications being as follows: The free-entry condition is replaced by an indifference condition; the value of an unmatched entrepreneur is not necessarily zero but positive in steady state and time-varying in the presence of aggregate uncertainty; the match value (i.e., the value of an entrepreneur) is affected by idiosyncratic productivity; and we have an additional equation for vacancy creation as a function of the entry probability of unmatched entrepreneurs. Notice, furthermore, that given our assumption of a uniform distribution, the entry probability, p, and the conditional expected value of a productivity draw, a^*, are straightforward to characterize analytically.[5]

The computation of the steady state now required solving for the cutoff productivity level and the hiring rate using the indifference equation. This step is implemented in the auxiliary function `fn_Cutoff`. The master file includes an option, `optionRecalibration`, for the user to specify whether or not the recommended recalibration procedure described in Section 4.2 is implemented. If it is not, then the steady state will not match the targeted calibration moments, such as the unemployment rate. The recalibration procedure ensures not only that this is the case, but also picks the strike value χ and worker bargaining power ω such that the steady-state elasticity of labor market tightness matches a targeted value (i.e., the same one used to calibrate the standard model). The auxiliary function `fn_omega_chi.m` serves this function, and it in turn calls the function `fn_Elasticity`, which solves for that elasticity using Matlab's symbolic toolbox.

To characterize some of the model properties – both in steady state and after hitting the economy with a shock – it proved interesting to loop over a range of vales for the standard deviation of idiosyncratic productivity, σ_a. The folder `Master_VersionWithLoops`, and in particular the file `main_SaMOptionValue_WithLoop.m`, therefore provides an extension of the baseline code which makes it easy to store and plot outcomes as a function of σ_a. For the user, the primary choices relate to the parameters `sigma_a_num`, `sigma_a_low` and `sigma_a_high`, which together specify the range of σ_a over which the loop is run. The code automatically plots the impact effect of an uncertainty shock on selected variables, but additional results (such as steady-state values or the steady-state elasticity of labor market tightness with respect to aggregate productivity) can be

[5] As described below, it is also possible without much difficulty to use a normal distribution instead.

accessed via the structure sResults.

Exact replication of figures in the paper.

Figure 4. In folder Figure_Loop_ComparisonToNash, run plot_SaM_OptionValue_CrossSectionalDispersionProperties.m. The file uses pre-computed inputs resultsOptionValue_p05_NoRecalib_sigmaa0To001_Fine.mat and resultsOptionValue_p05_NoRecalib_sigmaa00005To001_FixedH_Fine.mat (again, these are provided already), which were obtained using the master version allowing for loops. Notice that to keep the hiring rate fixed, comment out line 123 and comment in line 124 in the Dynare file dynareSAMOptionValue.mod.

Figure 5. In folder Master_Baseline, run main_SaMOptionValue.m for optionRecalibration='yes' and sPar.sigma_a=0.003.

Figure 6. In folder Figure_Loop_ComparisonToNash, run plot_SaMOptionValue_LoopFigureWithNash.m. Again, we draw on pre-computed inputs using the Dynare implementations contained in the folders Main_Section4_NewModel\Master_VersionWithLoops and Main_Section3_BaselineModel.

III Online appendices

Risk aversion

There are two different main codes relevant to this section. The file main_SaM_RiskAversion.m is structured similar to the ones already discussed in the context of the canonical SaM model; it sets various options (e.g., structural parameter values), calls a Dynare file (dynareSaM_riskAversion.mod), and plots the IRFs for a single model. The user can choose which variables to show.

The figures used in the paper often display IRFs from multiple different models. The file plot_SaM_RiskAversion.m may be used to this end. The code relies on having previously run main_SaM_RiskAversion.m several times; this has already been done for the reader, so that the replication package comes with all the relevant inputs (stored in Output\IRFs).

Exact replication of figures in the paper.

Figure OB.1. In plot_SaM_RiskAversion.m, set figureName as 'fig_SaM_RiskAversion_WageLin' and run. The two input IRFs are generated by running main_SaM_RiskAversion.m given a linear wage – which the user can select in dynareSaM_riskAversion.mod by setting @#defineOptionWNash=1 – with either a linear equity pricing equation, equivalent to setting sPar.xi=0 (line 44) or log utility, using sPar.xi=1.

Figure OB.2. In plot_SaM_RiskAversion.m, set figureName as 'fig_SaM_RiskAversion_WageComparison' and run. There are four input IRFs (see lines 57-72), each generated using main_SaM_RiskAversion.m and assuming log utility. IRFs_LogU_WageLin_Baseline is case with a linear wage rule; IRFs_LogU_WageNash_Baseline is the case with a standard Nash-bargained wage; IRFs_LogU_WageNash_RiskNeutralWage requires setting both @#defineOptionNashSDFConstant=1 and @#defineOptionNashMUConstant=1 in the Dynare file dynareSaM_riskAversion.mod; finally, IRFs_LogU_WageNash_MUConstantInNash only has @#defineOptionNashMUConstant=1 but @#defineOptionNashSDFConstant=0.

A two-period model with heterogeneity

All figures relating to the two-period model are created using straightforward computations, hence no further documentation is provided beyond the comments in the Matlab codes. All the figures essentially rely on the same steps, but separate codes are provided, because the positioning of various annotations is specific to each figure.

Exact replication of figures in the paper.

Figure OC.1a. Run main_2Period_ValueOfWaiting_FixedHiring.m

Figure OC.1b. Run `main_2Period_Cutoff_FixedHiring.m`

Figure OC.1c. Run `main_2Period_ValueOfWaiting_VariableHiring.m`

Figure OC.1d. Run `main_2Period_ValueOfWaiting_VariableHiring.m`

Variations to the heterogeneous-firm model

Infinitely-lived entrepreneurs

Replacing the assumption that entrepreneurs "die" upon separation and are replaced with infinitely-lived entrepreneurs entails three changes in the code files. First, the match value equation in the Dynare implementation includes an additional continuation value term; and when $\gamma > 0$, the vacancy-posting equation is likewise augmented. Second, the steady-state computation becomes slightly more involved, because the values of a matched and an unmatched entrepreneur need to be solved for jointly. And, finally, the recalibration files need to be tweaked to account for the adjusted match value equation. Two further notes: Firstly, and for completeness, we provide a separate code for the model with $\gamma = 0$, which is easier and faster to solve; and, secondly, in

Exact replication of figures in the paper.

Figure OD.1. `plot_SaMWithOptionValue_InfinitelyLived.m` calls on results created using the baseline code as well as, for the model with infinitely-lived entrepreneurs, the file `main_SaMOptionValue_gamma.m` in the folder `gamma_model`. They are stored in the folder `Output`, so that the reader can simply run `plot_SaMWithOptionValue_InfinitelyLived.m`. Alternatively, to manually create the IRFs for the extended model, run `main_SaMOptionValue_gamma.m` once with `sPar.gamma=0`, `optionRecalibration_type='standard'` and `sPar.sigma_a=0.025` (or simply run `main_SaMOptionValue_infinitelylived_gamma0` in the corresponding folder); and then with `sPar.gamma=0.999`, `optionRecalibration_type='fixed'` and `sPar.sigma_a=0.003`

Endogenous measure of entrepreneurs

Endogenizing the measure of entrepreneurs as proposed in the online appendix is straightforward. The Dynare file is extended to include one additional, static equation (see `dynareSAMOptionValue.mod`); and the steady-state calibration follows immediately (see `main_SaMOptionValue_extension_upsilon.m`)

Exact replication of figures in the paper.

Figure OD.2. `plot_SaMOptionValue_EndoUpsilon.m` calls on results created using the (extended) master code. In particular, that code is run for three different values of ι, fixing the remainder of the parameters at their baseline values.

Robustness exercises

Stochastic vs. non-stochastic hiring specification

As described in Footnote OF.2, implementing the non-stochastic instead of the baseline, stochastic hiring specification requires adjustments to both the dynamic and the steady state equations. This replication code folder therefore includes suitably adjusted versions of the Dynare files, functions used in the recalibration, etc. The main file, `mainSaMOptionValue.m`, is structured analogous to the master code and can be used to solve the model once and compute IRFs or, instead, run a loop over different values of σ_a.

Exact replication of figures in the paper.

Figures OF.1a and OF.1b. The code for this figure is stored together with the other codes for the two-period version. The relevant file is `main_2Period_ValueOfWaiting_VariableHiring.m`.

Figure OF.1c. `plotting_SaMWithOptionValue_NonStochasticHiring.m` calls on results created using both the standard/master code and the adjusted version. They are stored in the folder `Inputs`.

Normal distribution

Assuming a normal instead of a uniform distribution for idiosyncratic productivity implies two main changes in terms of the numerical implementation. First, we draw on Matlab's internal `normcdf` function to compute the entry probability instead of being able to rely on analytical expressions. This affects both the steady state computation and the expressions used in the Dynare file. Second, in our recalibration procedure, we now need to compute the elasticity of labor market tightness with respect to aggregate productivity numerically (see file `fnElasticity.m`). This replication code folder therefore includes suitably adjusted versions of the Dynare files, functions used in the recalibration, etc. The master code is `main_SaMOptionValue_Normal.m`.

Exact replication of figures in the paper.

Figure OF.2. `plot_SaMOptionValue_Normal.m` calls on results created using the master code, and stored in the folder `Inputs`. In particular, the standard IRFs are computed given $\sigma_a = 0.001$

and using either a normal distribution for a (using the master code in this folder) or the uniform specification (using the baseline code).

Different degree of cross-sectional dispersion

This figure is straightforward to create, just as Figure 5, that is, using the master code.

Exact replication of figures in the paper.
Figure OF.3. In folder `Master_Baseline`, run `main_SaMOptionValue.m` for `optionRecalibration='yes'` and `sPar.sigma_a=0.001`.

Different steady-state entry probability

Changing the targeted steady-state entry probability can simply be done in the master code.

Exact replication of figures in the paper.
Figure OF.4. `plot_SaMWithOptionValue_LowerEntryProbability.m` calls on results created using the master code, and stored in the folder `Inputs`. In particular, the standard IRFs are computed given $\sigma_a = 0.001$ and targeting a steady-state entry probability of either 0.2 or 0.5. Throughout, the recalibration procedure is employed.

References for documentation

Adjemian, S., Bastani, H., Juillard, M., Karamé, F., Maih, J., Mihoubi, F., Perendia, G., Pfeifer, J., Ratto, M., and Villemot, S. (2018). Dynare: Reference Manual Version 4. Technical Report 1, CEPREMAP.

Andreasen, M. M., Fernández-Villaverde, J., and Rubio-Ramírez, J. F. (2018). The Pruned State-Space System for Non-Linear DSGE Models: Theory and Empirical Applications. *The Review of Economic Studies*, **85**(1), 1–49.

Basu, S. and Bundick, B. (2017). Uncertainty Shocks in a Model of Effective Demand. *Econometrica*, **85**(3), 937–958.

Born, B. and Pfeifer, J. (2014). Risk Matters: The Real Effects of Volatility Shocks: Comment. *American Economic Review*, **104**(12), 4231–4239.
