# Supplementary figures and images for: Volatile hiring: uncertainty in search and matching models
Source: J Monet Econ. 2021 Oct;123:1–18. doi: 10.1016/j.jmoneco.2021.07.008 (PMC8547261; doi:10.1016/j.jmoneco.2021.07.008)

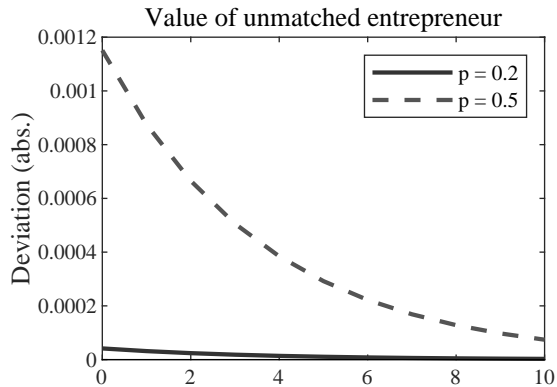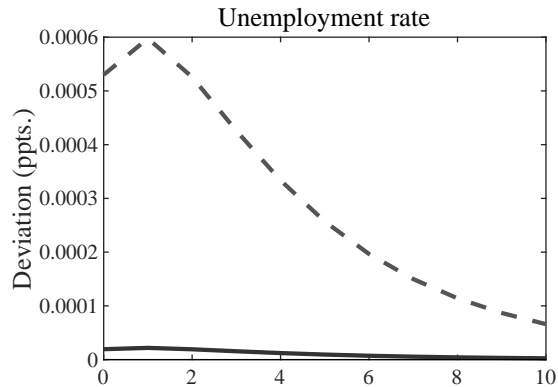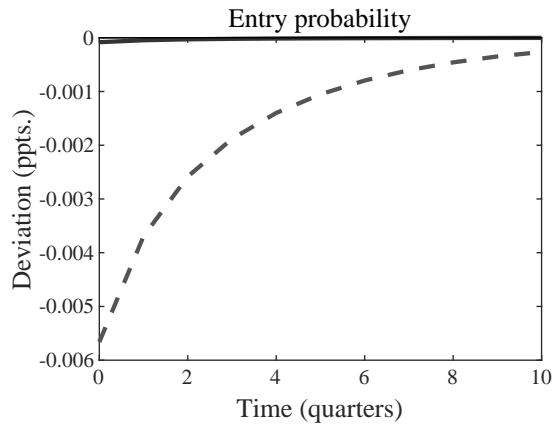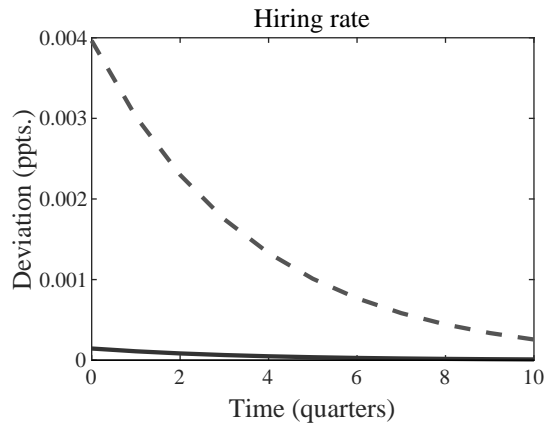

Supplement: Supplementary file 2 [file mmc2.zip › ReplicationKit/Appendices/Appendix_DifferentSSEntryProb/Output/fig_App_P02vsP05_Recalib_sigmaa001.pdf]

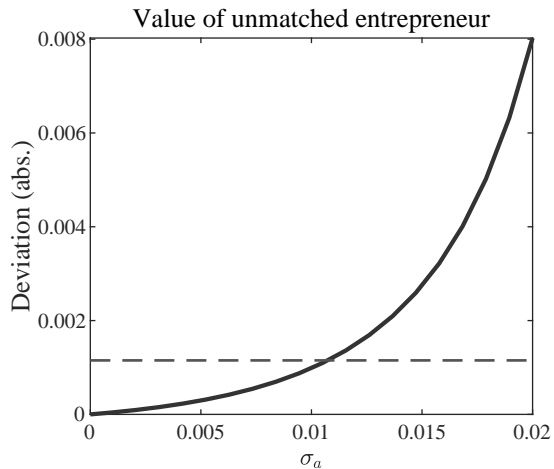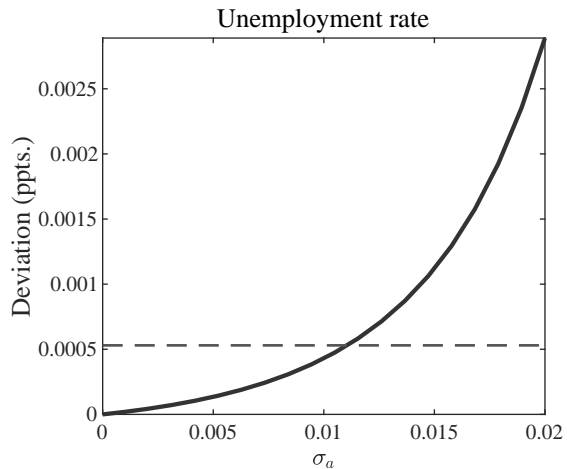

Supplement: Supplementary file 2 [file mmc2.zip › ReplicationKit/Appendices/Appendix_DifferentSSEntryProb/Output/fig_App_Recalib_Loop0to002_WithComparison.pdf]

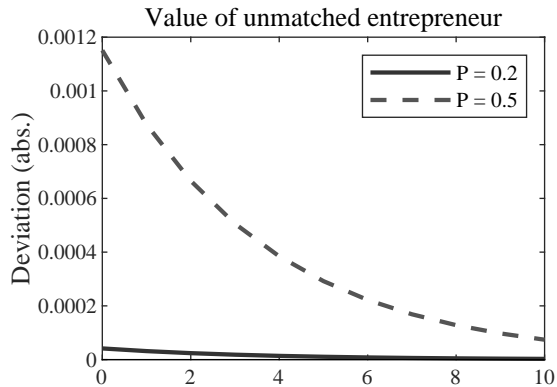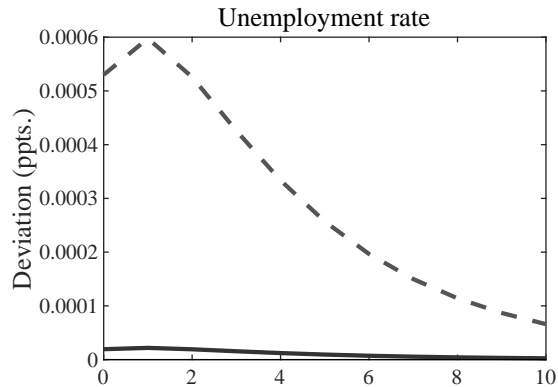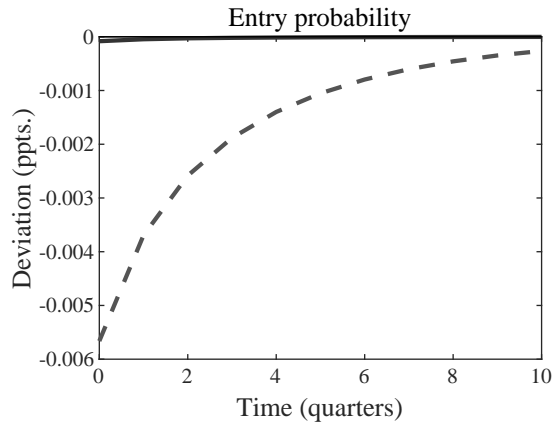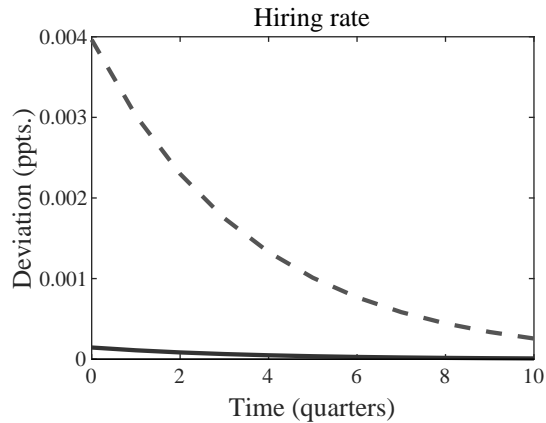

Supplement: Supplementary file 2 [file mmc2.zip › ReplicationKit/Appendices/Appendix_DifferentSSEntryProb/Output/fig_SaMOptionValue_P02vsP05_Recalib_sigmaa001_2x2.pdf]

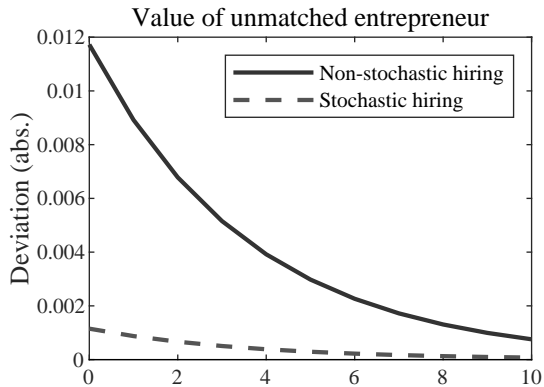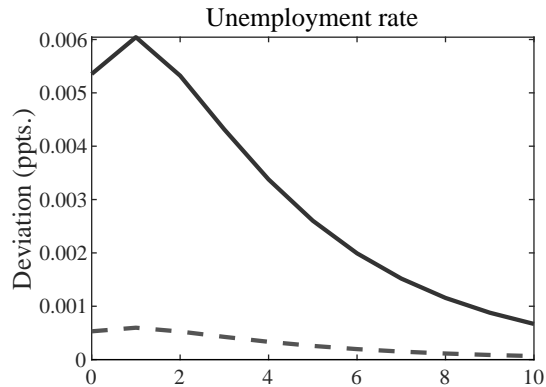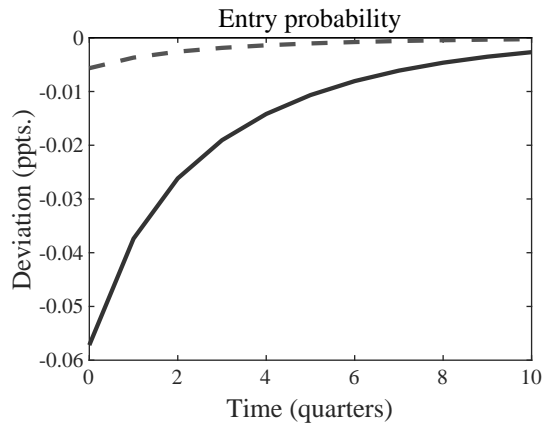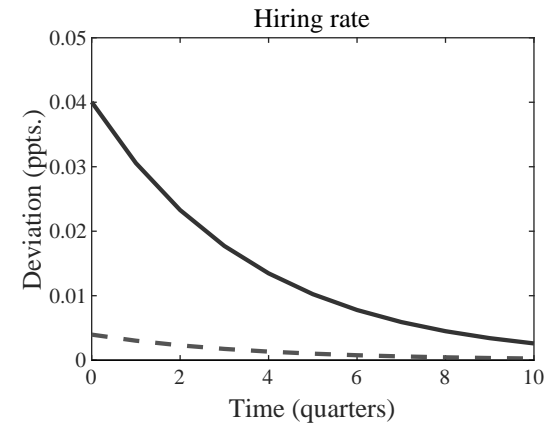

Supplement: Supplementary file 2 [file mmc2.zip › ReplicationKit/Appendices/Appendix_HiringSpecifications/Output/Figures/fig_App_sigmaa001_Recalib_NonStochasticHiring.pdf]

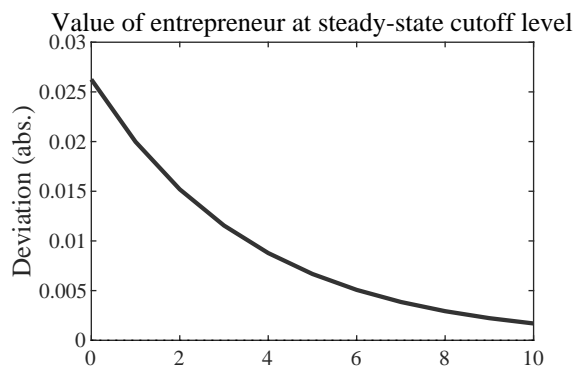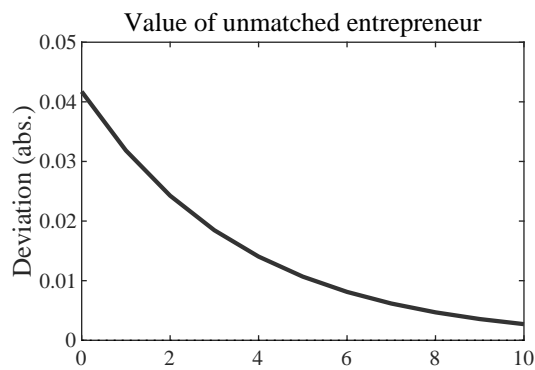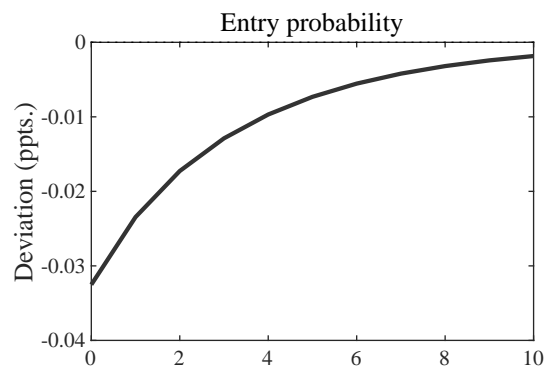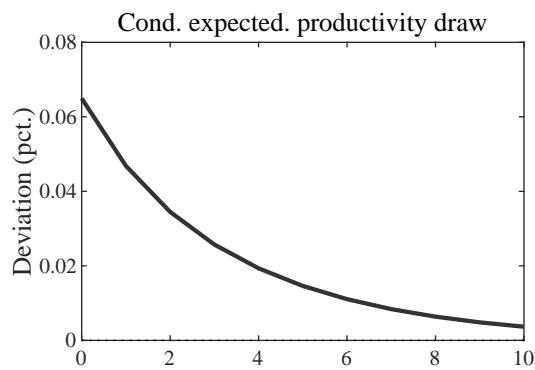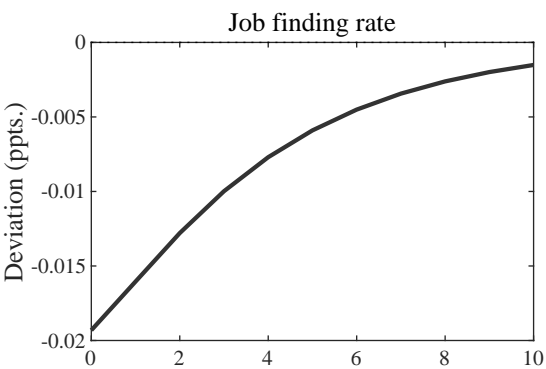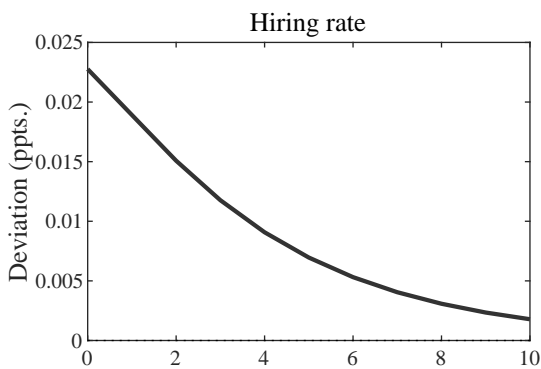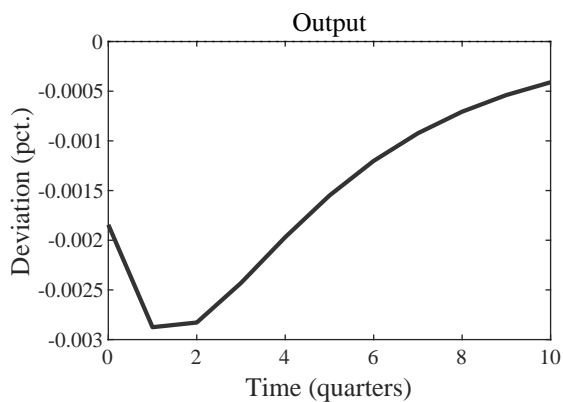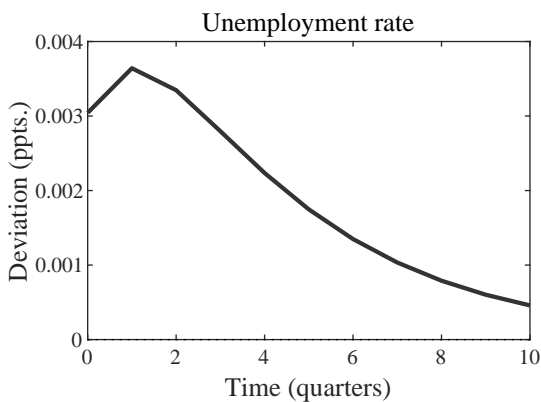

Supplement: Supplementary file 2 [file mmc2.zip › ReplicationKit/Appendices/Appendix_InfinitelyLived/gamma_model/Output/Figures/fig_HetFirm_GammaModel_sigma025_gamma0_recalib.pdf]

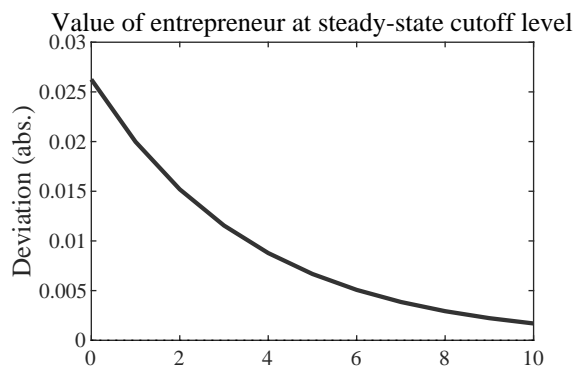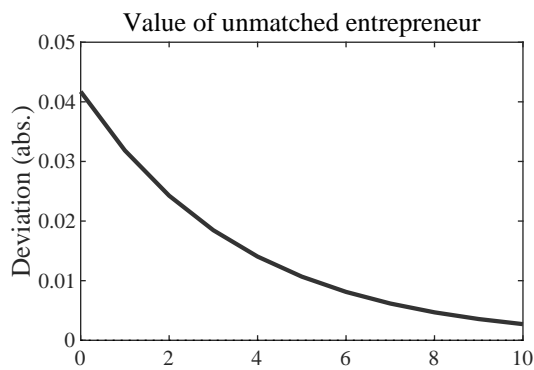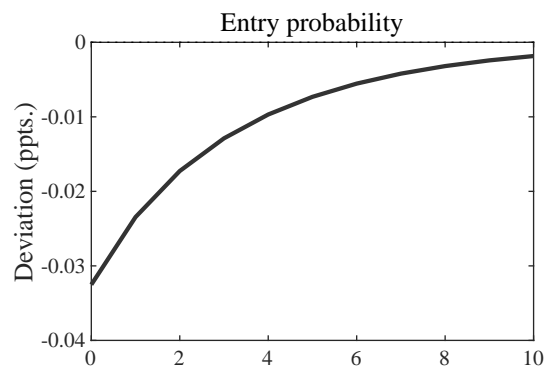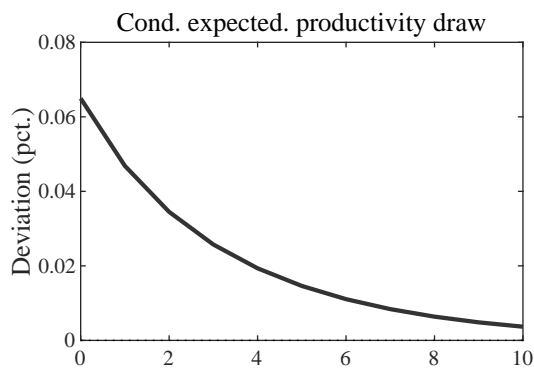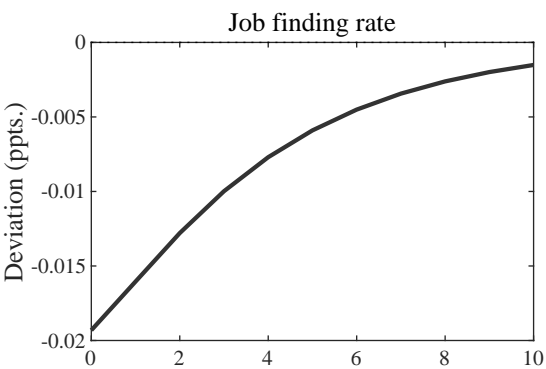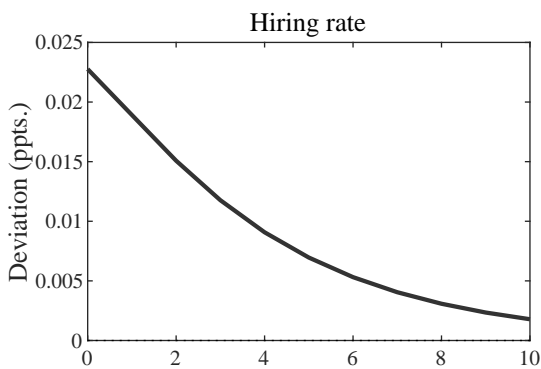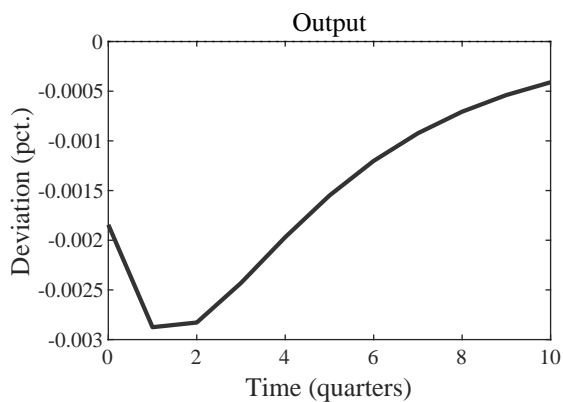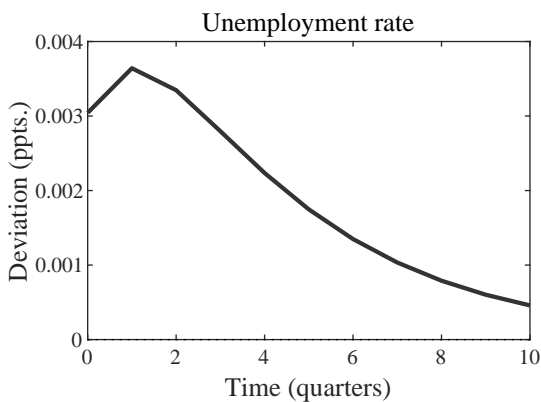

Supplement: Supplementary file 2 [file mmc2.zip › ReplicationKit/Appendices/Appendix_InfinitelyLived/gamma0_model/Output/Figures/fig_SaM_HetFirm_p05_NoDeath_sigmaa0025_Recalib.pdf]

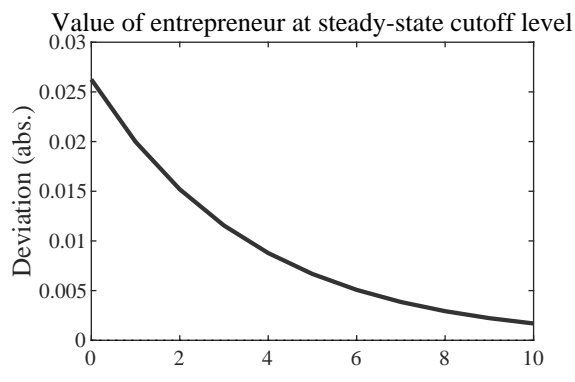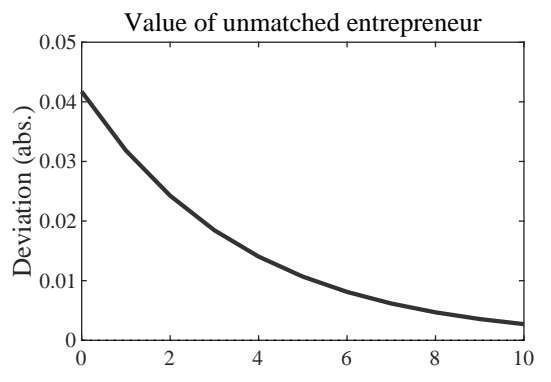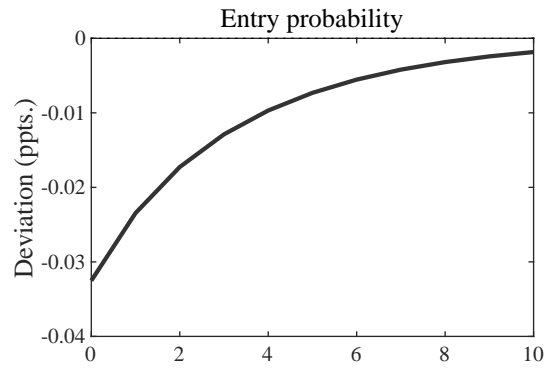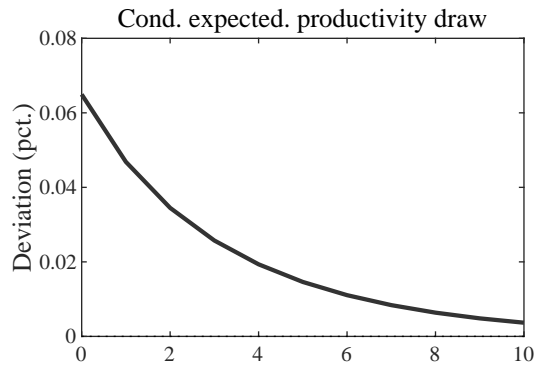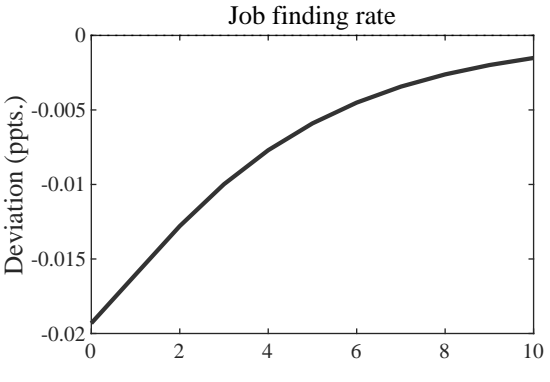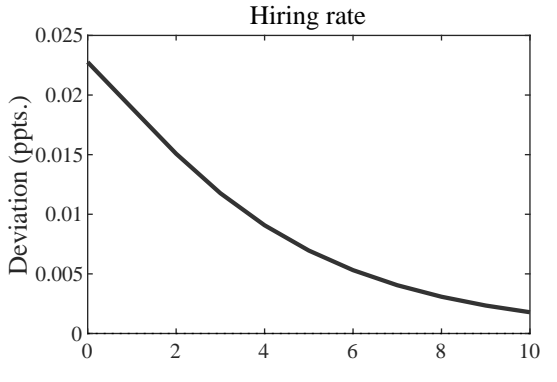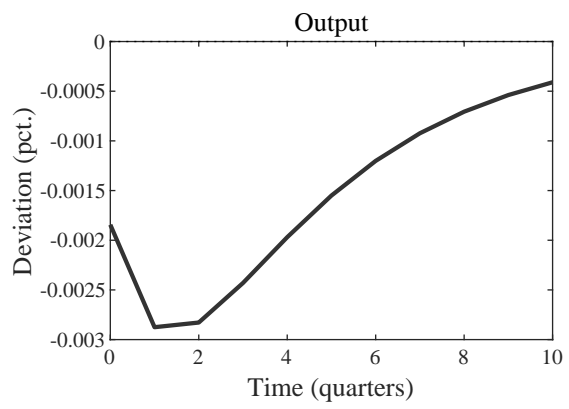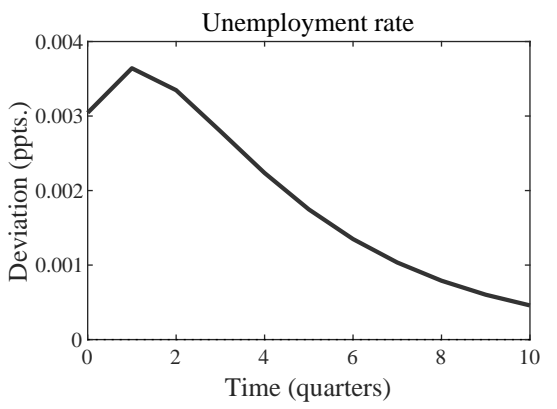

Supplement: Supplementary file 2 [file mmc2.zip › ReplicationKit/Appendices/Appendix_InfinitelyLived/gamma0_model/Output/Figures/fig_SaM_OptionValue_p05_NoDeath_sigmaa0025_Recalib.pdf]

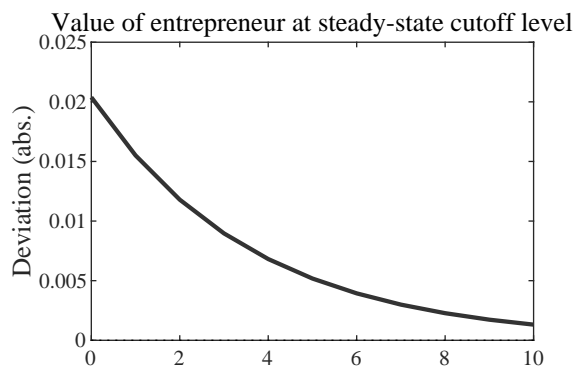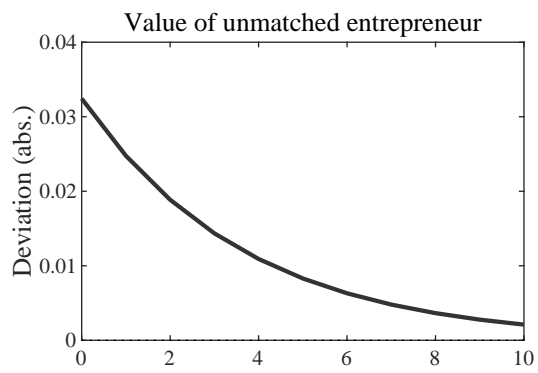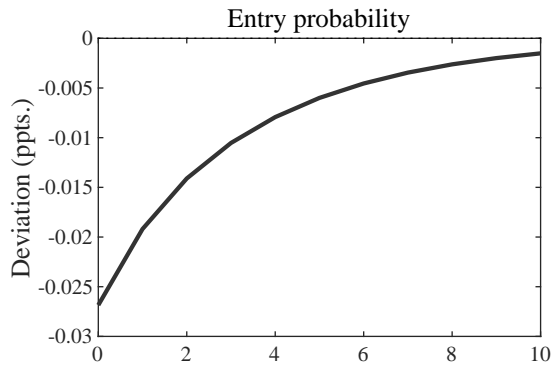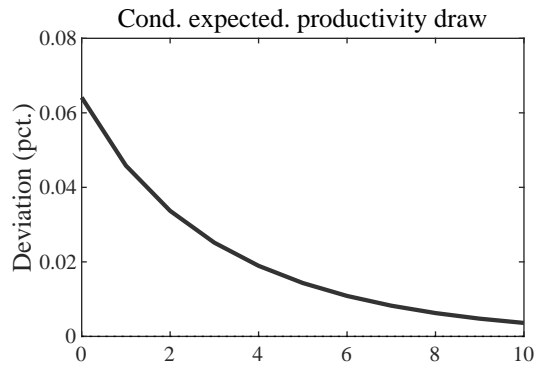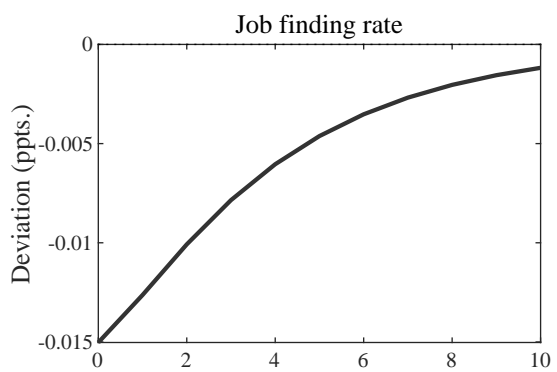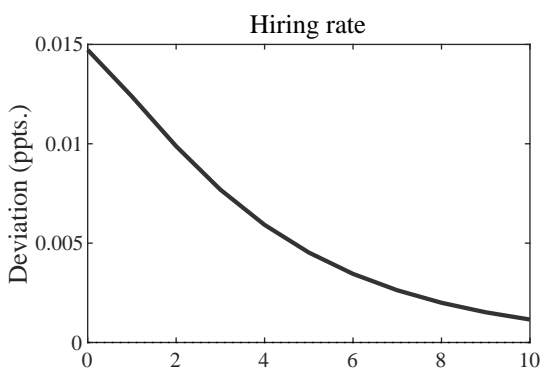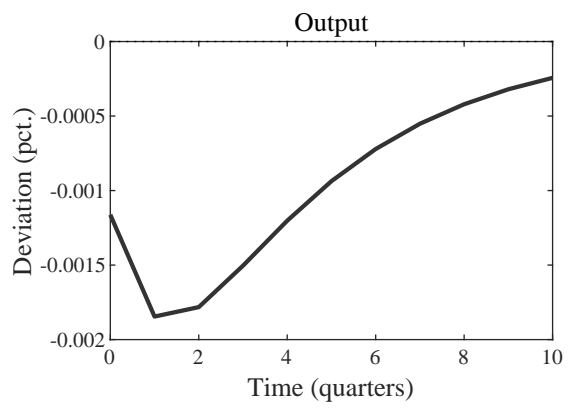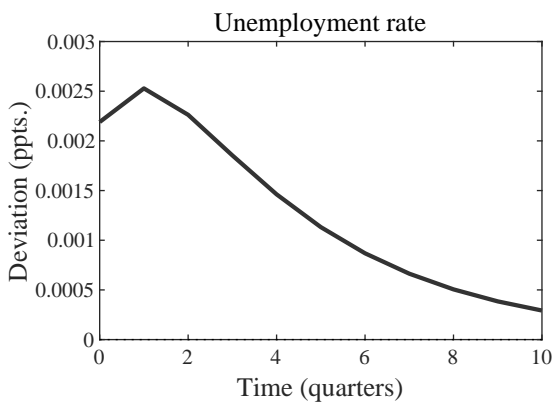

Supplement: Supplementary file 2 [file mmc2.zip › ReplicationKit/Appendices/Appendix_InfinitelyLived/gamma0_model/Output/Figures/IRFs_SaMOptionValue_Uniform_NoDeath_p05_sigmaa025_RecalibAsInBaselineWith003.pdf]

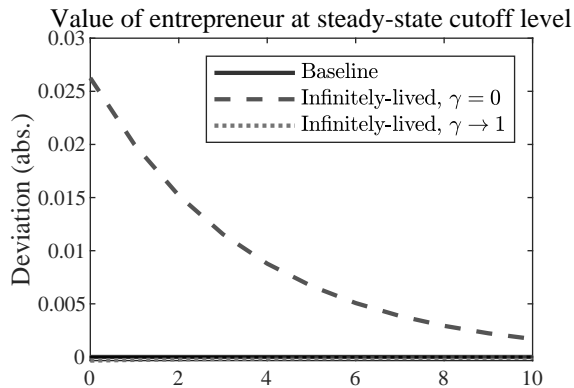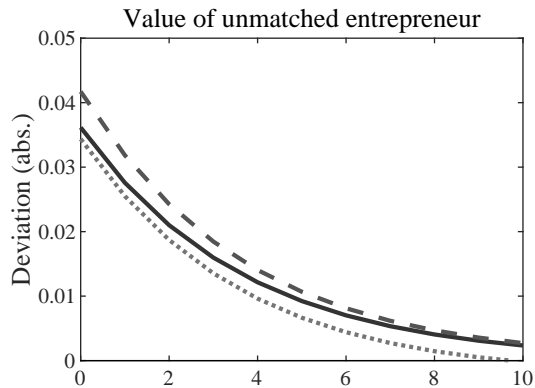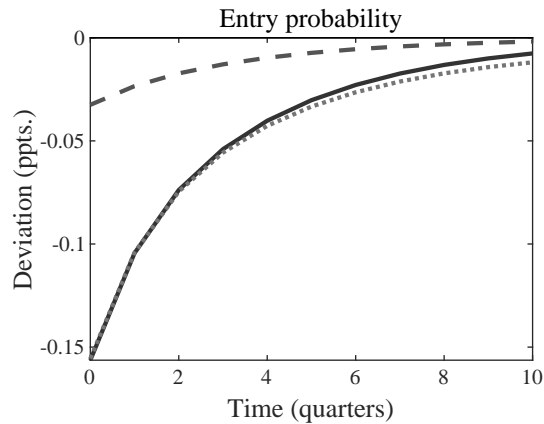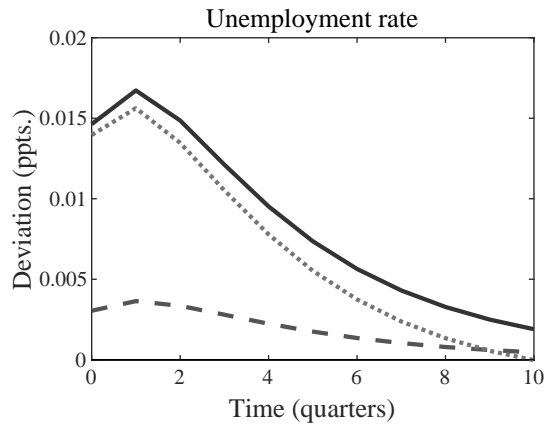

Supplement: Supplementary file 2 [file mmc2.zip › ReplicationKit/Appendices/Appendix_InfinitelyLived/Output/Fig_App_HetFirm_Comparison_InfinitelyLived.pdf]

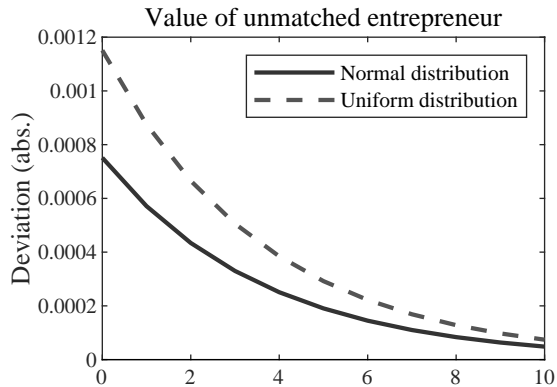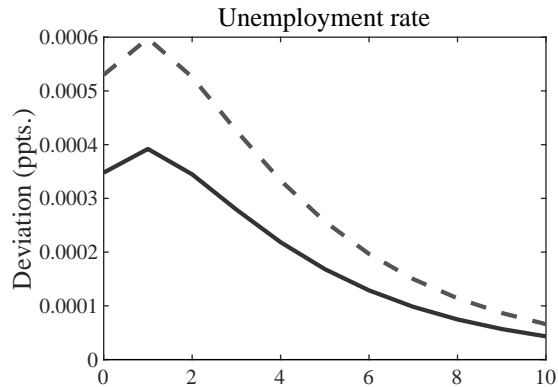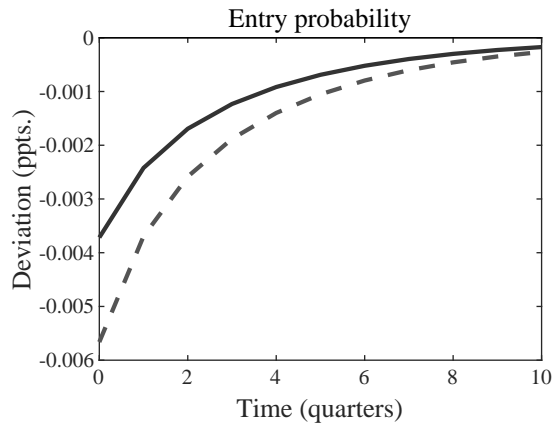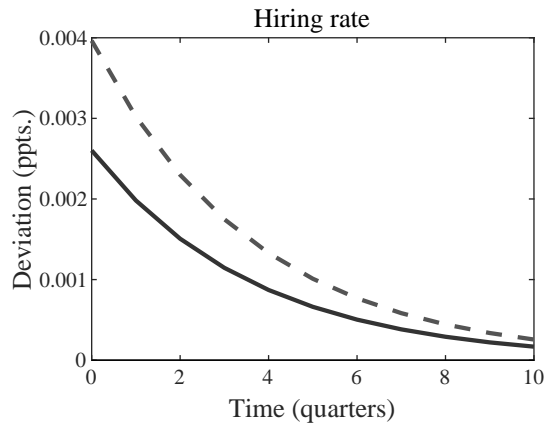

Supplement: Supplementary file 2 [file mmc2.zip › ReplicationKit/Appendices/Appendix_NormalDistribution/Output/Figures/fig_App_Normal_NoRecalib_Loopto01.pdf]

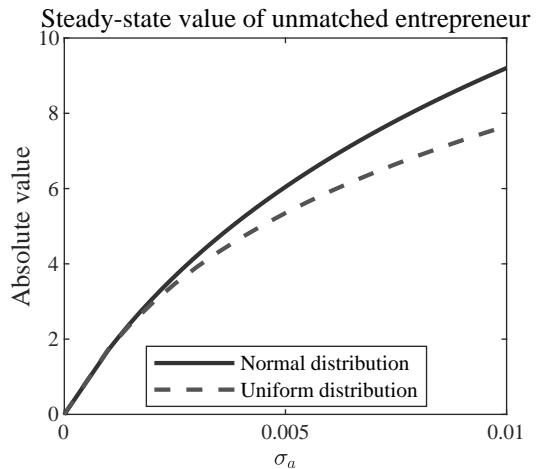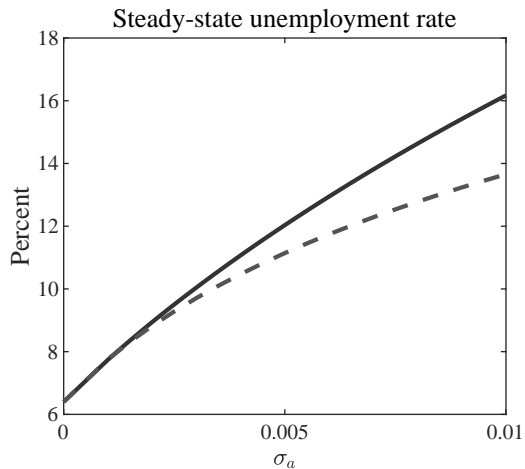

Supplement: Supplementary file 2 [file mmc2.zip › ReplicationKit/Appendices/Appendix_NormalDistribution/Output/Figures/fig_App_Normal_Recalib_sigma001.pdf]

Match value

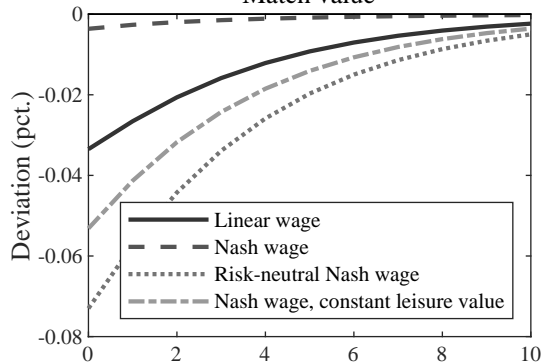

Wage

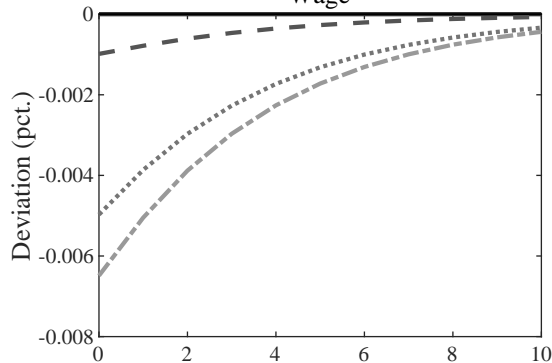

Unemployment rate

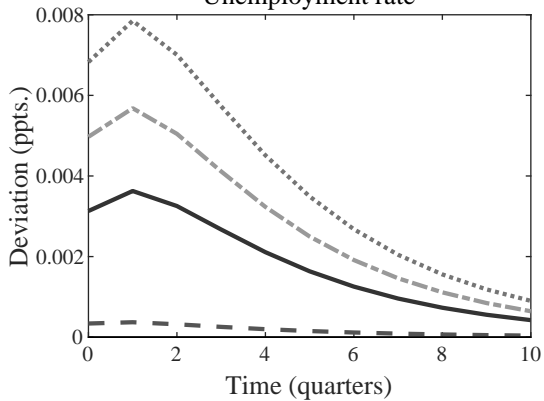

Consumption

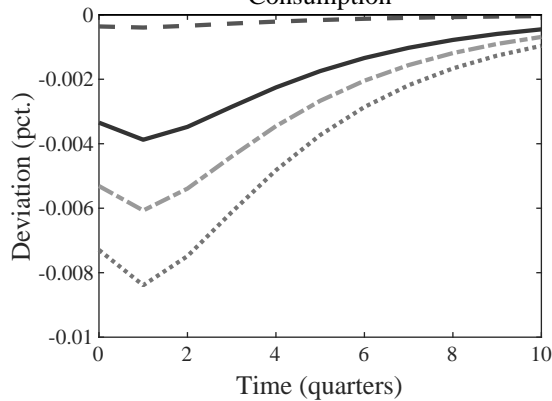

Supplement: Supplementary file 2 [file mmc2.zip › ReplicationKit/Appendices/Appendix_RiskAversion/Output/Figures/fig_SaM_RiskAversion_WageComparison.pdf]

Match value

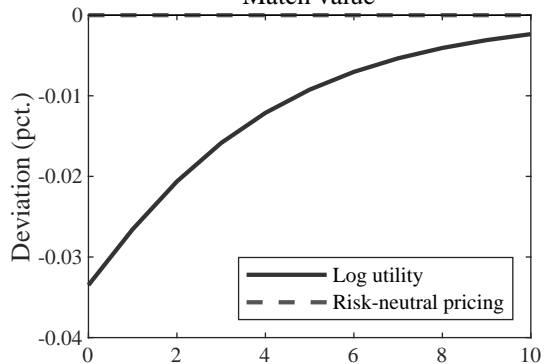

Wage

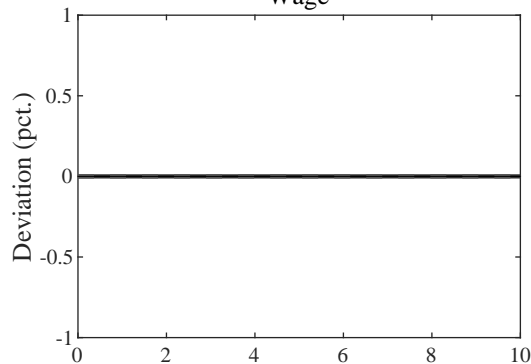

Unemployment rate

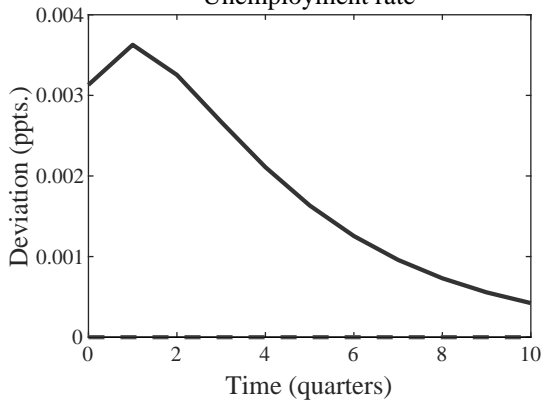

Consumption

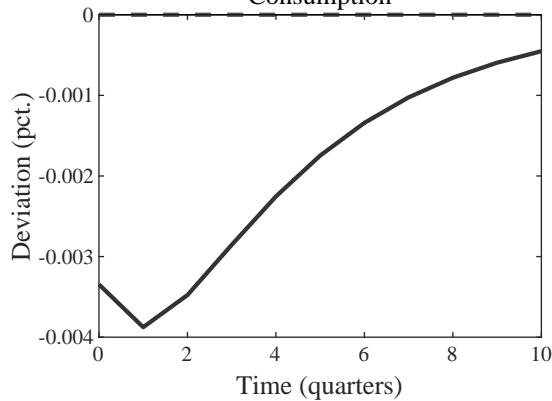

Supplement: Supplementary file 2 [file mmc2.zip › ReplicationKit/Appendices/Appendix_RiskAversion/Output/Figures/fig_SaM_RiskAversion_WageLin.pdf]

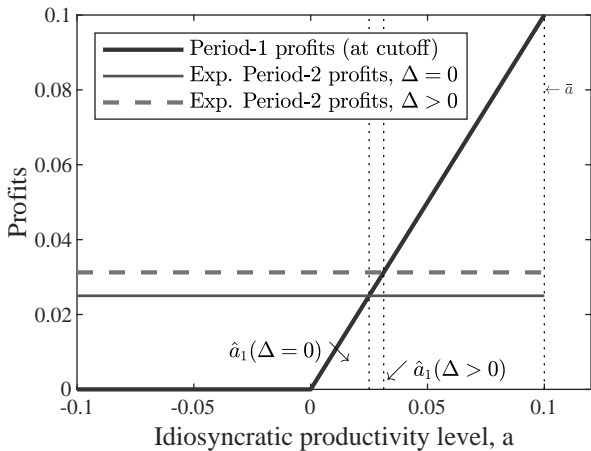

Supplement: Supplementary file 2 [file mmc2.zip › ReplicationKit/Appendices/Appendix_TwoPeriodModel/Output/fig_App_2P_CutoffDetermination_hFixed_hBar1_aBar01_Delta005.pdf]

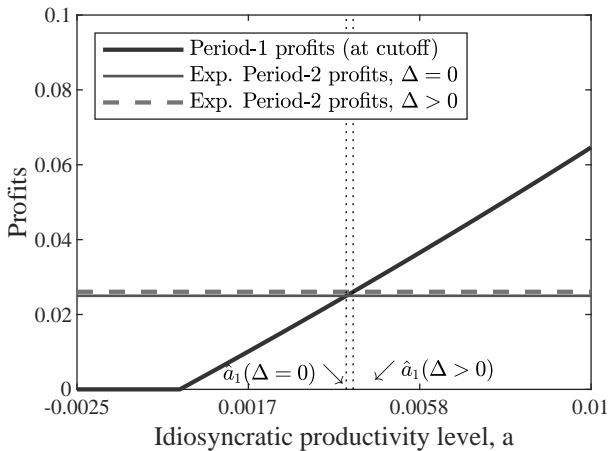

Supplement: Supplementary file 2 [file mmc2.zip › ReplicationKit/Appendices/Appendix_TwoPeriodModel/Output/fig_App_2P_CutoffDetermination_hVariable_alpha05_hBar1_aBar01_Delta015.pdf]

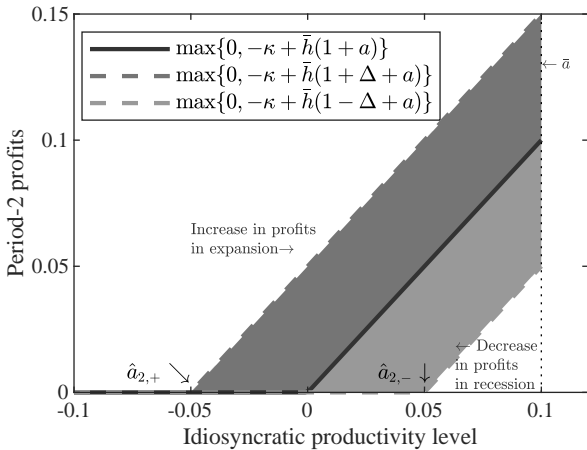

Supplement: Supplementary file 2 [file mmc2.zip › ReplicationKit/Appendices/Appendix_TwoPeriodModel/Output/fig_App_2P_Period2Profits_hFixed_hBar1.pdf]

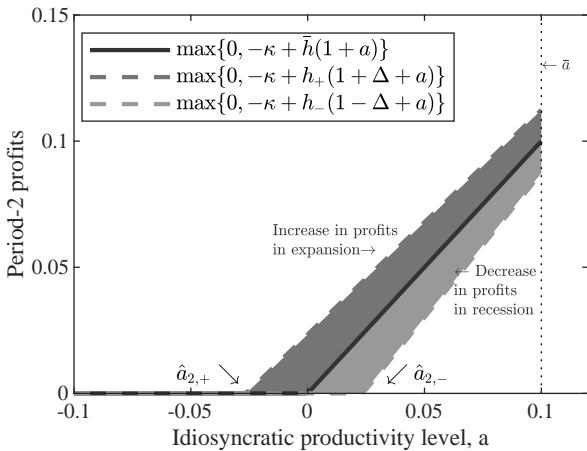

Supplement: Supplementary file 2 [file mmc2.zip › ReplicationKit/Appendices/Appendix_TwoPeriodModel/Output/fig_App_2P_Period2Profits_hVariable_alpha05_hBar1_aBar01_Delta015.pdf]

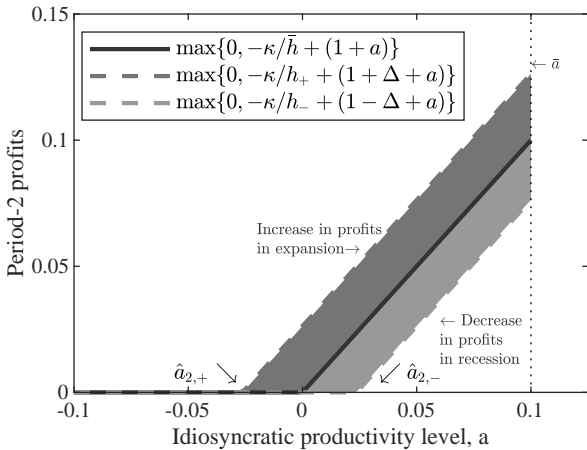

Supplement: Supplementary file 2 [file mmc2.zip › ReplicationKit/Appendices/Appendix_TwoPeriodModel/Output/fig_App_2P_Period2Profits_hVariable_alpha05_hBar1_aBar01_Delta015_NonStochasticHiring.pdf]

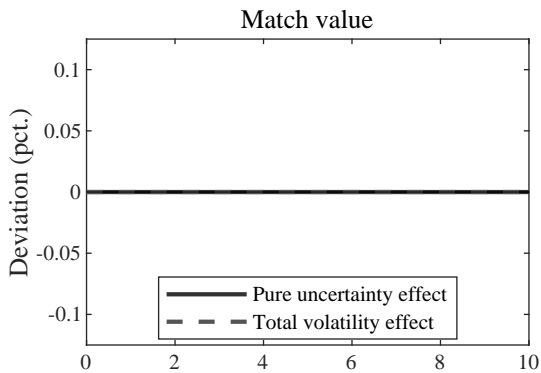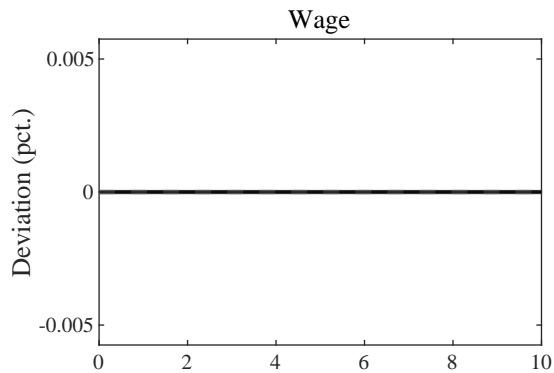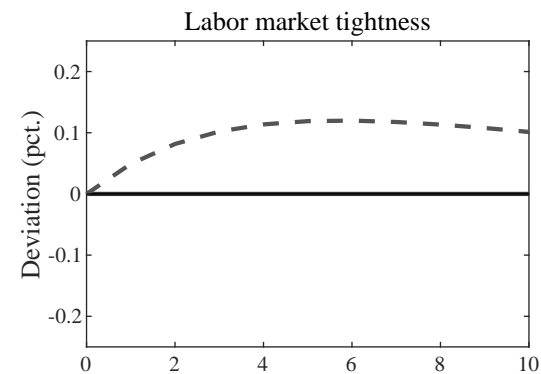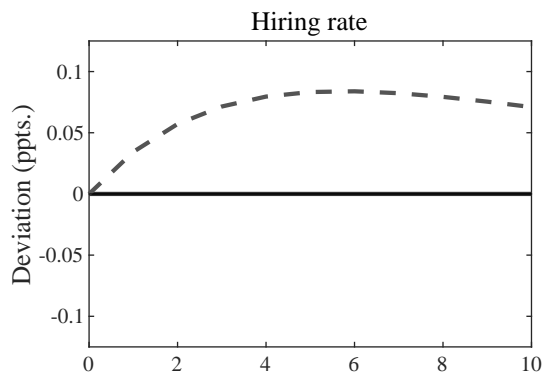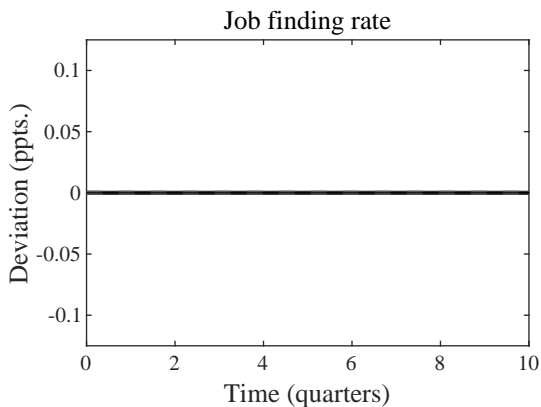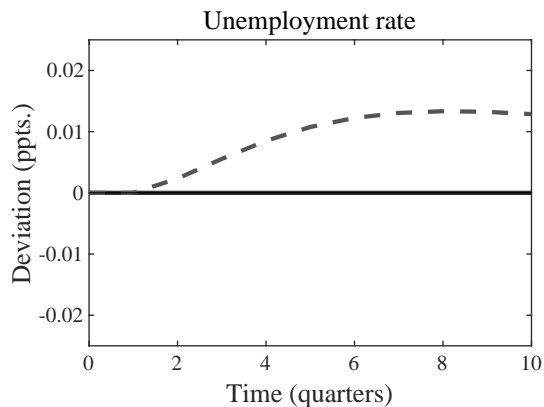

Supplement: Supplementary file 2 [file mmc2.zip › ReplicationKit/Main_Section3_BaselineModel/Output/Figures/fig_Baseline_WLin.pdf]

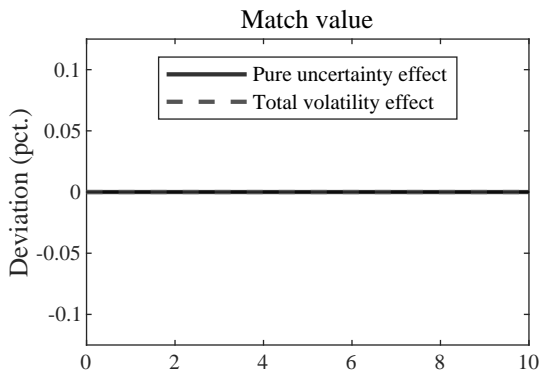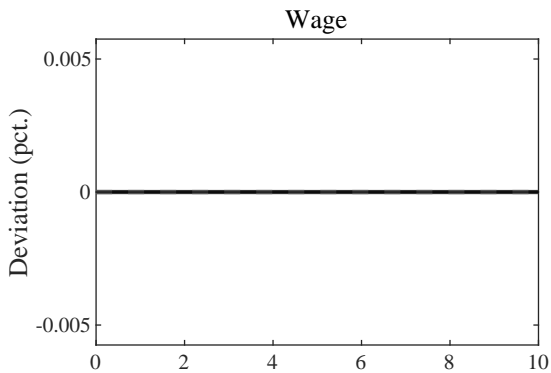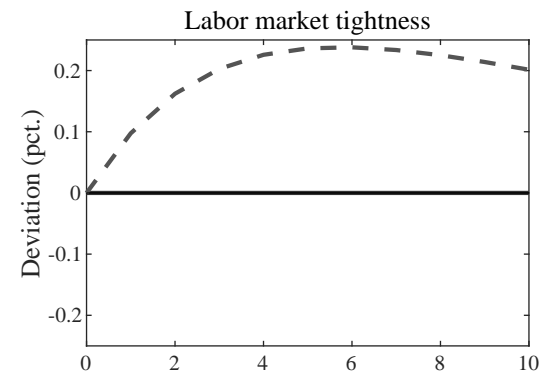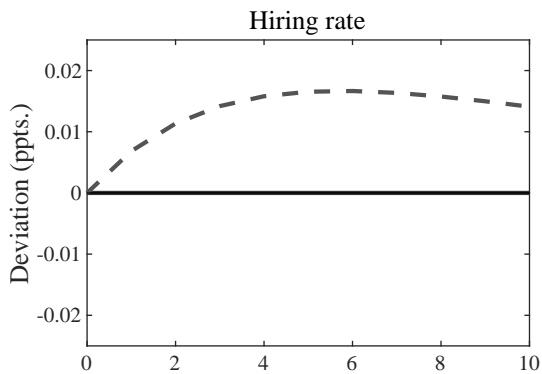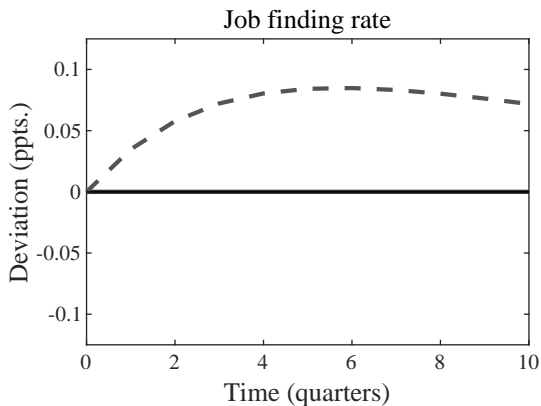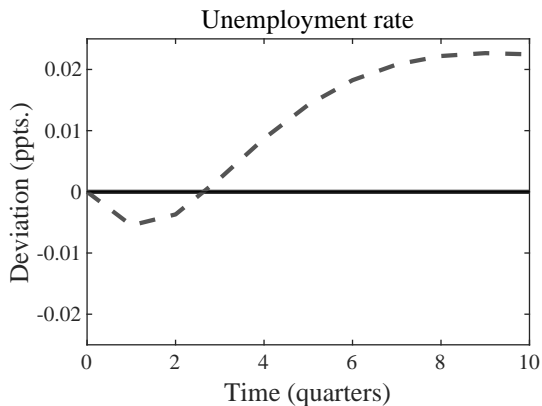

Supplement: Supplementary file 2 [file mmc2.zip › ReplicationKit/Main_Section3_BaselineModel/Output/Figures/fig_Baseline_WLin_alpha02.pdf]

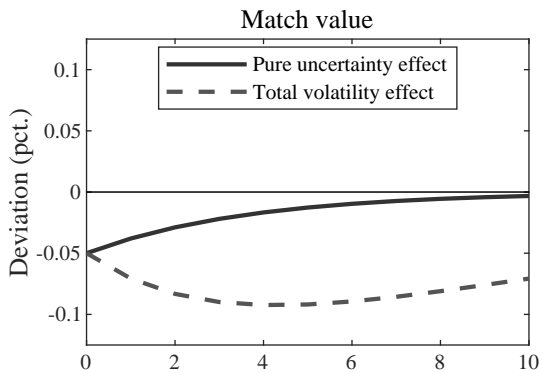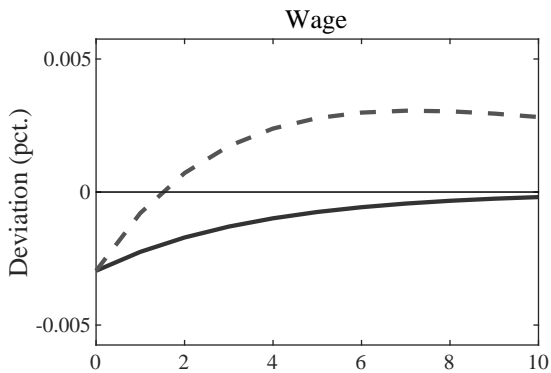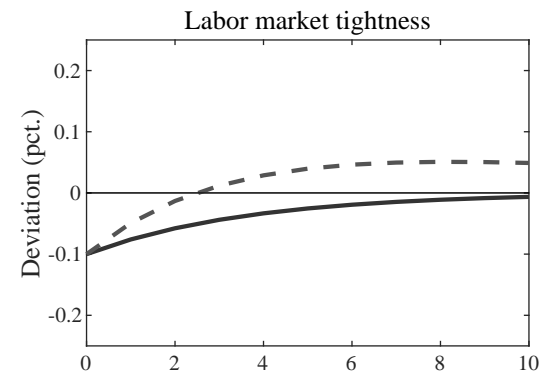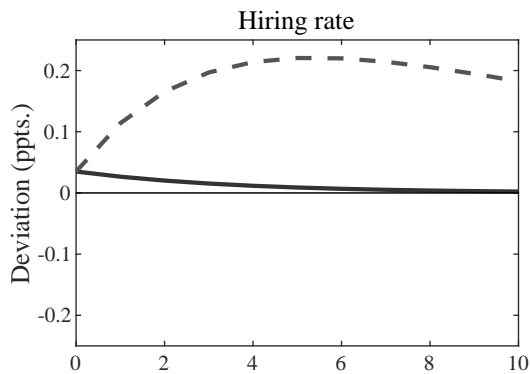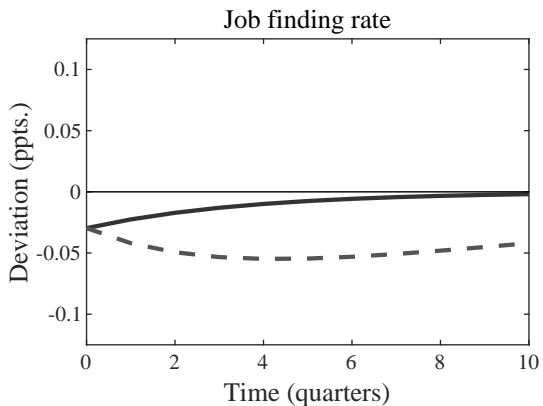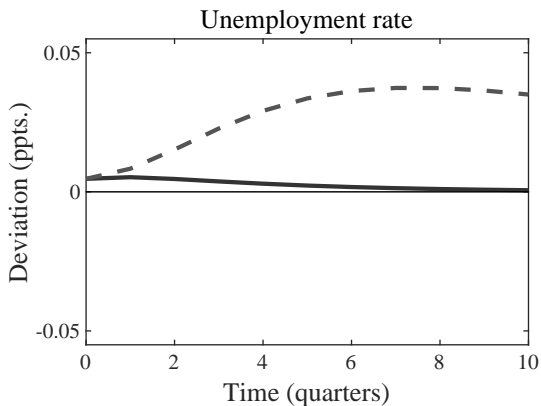

Supplement: Supplementary file 2 [file mmc2.zip › ReplicationKit/Main_Section3_BaselineModel/Output/Figures/fig_Baseline_WNash.pdf]
