# Supplementary material for: Volatile hiring: uncertainty in search and matching models
Source: J Monet Econ. 2021 Oct;123:1–18. doi: 10.1016/j.jmoneco.2021.07.008 (PMC8547261; doi:10.1016/j.jmoneco.2021.07.008)
Supplement: Supplementary file 2 [file mmc2.zip › ReplicationKit/Main_Section4_NewModel/Figure_Loop_ComparisonToNash/Output/fig_SaM_OptionValue_ComparisonToNash.pdf]

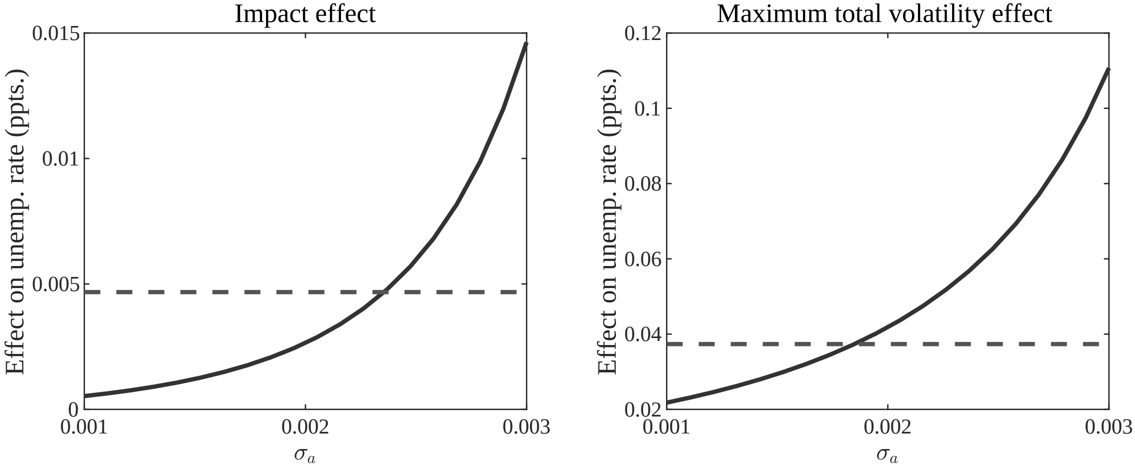

Impact effect
Effect on unemp. rate (ppts.)
0.015
0.01
0.005
0
0.001
0.002
0.003
σ_a
Maximum total volatility effect
Effect on unemp. rate (ppts.)
0.12
0.1
0.08
0.06
0.04
0.02
0.001
0.002
0.003
σ_a
