# Supplementary material for: Volatile hiring: uncertainty in search and matching models
Source: J Monet Econ. 2021 Oct;123:1–18. doi: 10.1016/j.jmoneco.2021.07.008 (PMC8547261; doi:10.1016/j.jmoneco.2021.07.008)
Supplement: Supplementary file 2 [file mmc2.zip › ReplicationKit/Main_Section4_NewModel/Figure_Loop_CrossSectionalDispersion/Output/fig_SaMOptionValue_NoRecalib_Properties_1x2.pdf]

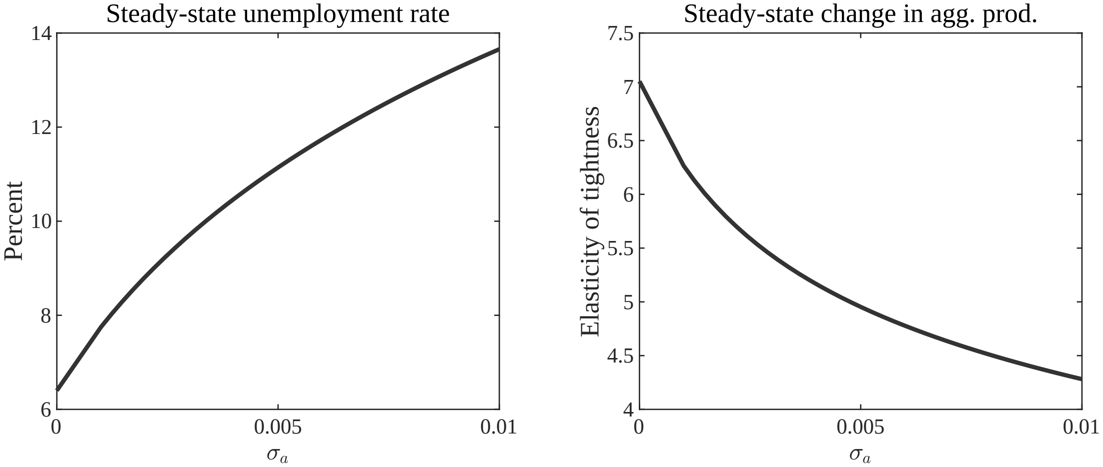
Steady-state unemployment rate
Percent
14
12
10
8
6
0
0.005
0.01
σ_a
Steady-state change in agg. prod.
Elasticity of tightness
7.5
7
6.5
6
5.5
5
4.5
4
0
0.005
0.01
σ_a
