# Supplementary material for: Volatile hiring: uncertainty in search and matching models
Source: J Monet Econ. 2021 Oct;123:1–18. doi: 10.1016/j.jmoneco.2021.07.008 (PMC8547261; doi:10.1016/j.jmoneco.2021.07.008)
Supplement: Supplementary file 2 [file mmc2.zip › ReplicationKit/Main_Section4_NewModel/Master_Baseline/Output/Figures/fig_SaM_OptionValue_p05_sigmaa0003_Recalib.pdf]

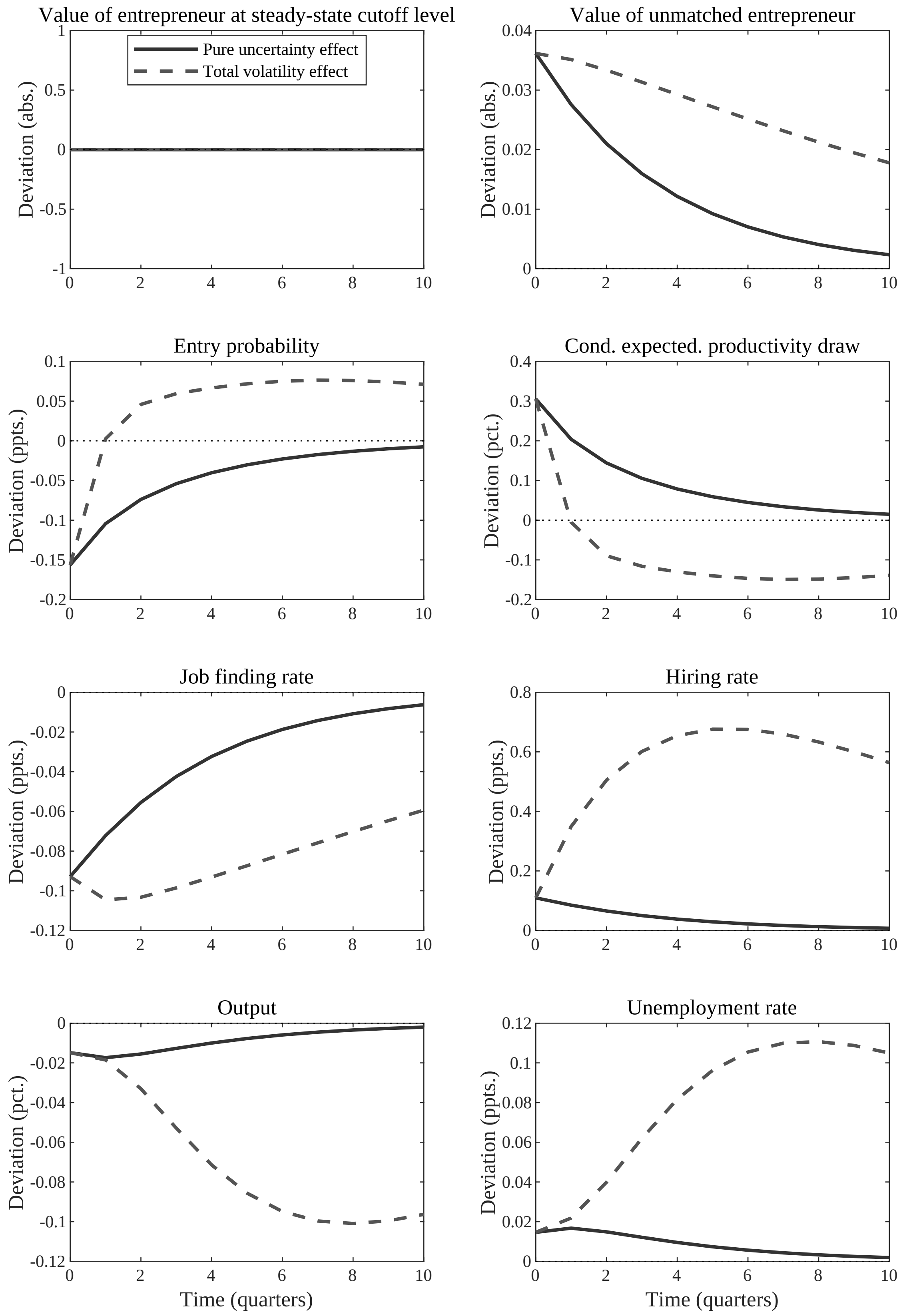

Value of entrepreneur at steady-state cutoff level
Pure uncertainty effect
Total volatility effect
Deviation (abs.)
Value of unmatched entrepreneur
Deviation (abs.)
Entry probability
Deviation (ppts.)
Cond. expected. productivity draw
Deviation (pct.)
Job finding rate
Deviation (ppts.)
Hiring rate
Deviation (ppts.)
Output
Deviation (pct.)
Time (quarters)
Unemployment rate
Deviation (ppts.)
Time (quarters)
